# Cryo-EM structure of the plant 26S proteasome

**DOI:** 10.1016/j.xplc.2022.100310

**Published:** 2022-03-11

**Authors:** Susanne Kandolf, Irina Grishkovskaya, Katarina Belačić, Derek L. Bolhuis, Sascha Amann, Brent Foster, Richard Imre, Karl Mechtler, Alexander Schleiffer, Hemant D. Tagare, Ellen D. Zhong, Anton Meinhart, Nicholas G. Brown, David Haselbach

**Affiliations:** 1 Research Institute of Molecular Pathology (IMP), Vienna BioCenter (VBC), Campus-Vienna-BioCenter 1, 1030 Vienna, Austria; 2 Department of Radiology and Biomedical Imaging, Yale University, New Haven, CT 06510, USA; 3 Department of Pharmacology and Lineberger Comprehensive Cancer Center, University of North Carolina School of Medicine, Chapel Hill, NC 27599, USA; 4 Computer Science and Artificial Intelligence Laboratory, Massachusetts Institute of Technology, Cambridge, MA 02139, USA; 5 Vienna BioCenter PhD Program, Doctoral School of the University at Vienna and Medical University of Vienna, Vienna BioCenter (VBC), Vienna, Austria; 6 Institute of Physical Chemistry, University of Freiburg, Albertstraße 21, Freiburg 79104, Germany; 7 CIBSS Centre for Integrative Biological Signalling Studies, University of Freiburg, Freiburg, Germany

**Keywords:** 26S proteasome, spinach, UPS, cryo-EM, conformational landscape

## Abstract

Targeted proteolysis is a hallmark of life. It is especially important in long-lived cells that can be found in higher eukaryotes, like plants. This task is mainly fulfilled by the ubiquitin–proteasome system. Thus, proteolysis by the 26S proteasome is vital to development, immunity, and cell division. Although the yeast and animal proteasomes are well characterized, there is only limited information on the plant proteasome. We determined the first plant 26S proteasome structure from *Spinacia oleracea* by single-particle electron cryogenic microscopy at an overall resolution of 3.3 Å. We found an almost identical overall architecture of the spinach proteasome compared with the known structures from mammals and yeast. Nevertheless, we noticed a structural difference in the proteolytic active β1 subunit. Furthermore, we uncovered an unseen compression state by characterizing the proteasome’s conformational landscape. We suspect that this new conformation of the 20S core protease, in correlation with a partial opening of the unoccupied gate, may contribute to peptide release after proteolysis. Our data provide a structural basis for the plant proteasome, which is crucial for further studies.

## Teaser

This study presents the first high-resolution structure of the plant 26S proteasome. The overall architecture appears conserved between higher plants and humans, although some small but significant differences can be observed. Among them is a new conformational mode.

## Introduction

The ubiquitin–proteasome system (UPS) is the most critical pathway for non-lysosomal protein degradation in all eukaryotes. With the help of a three-step conjugation cascade involving E1, E2, and E3 enzymes, protein substrates are labeled with a ubiquitin chain, which serves as a reusable recognition signal for selective protein turnover by the 26S proteasome (Ozaki et al., 1992; Vierstra, 1993). Most components of this system are well conserved in all branches of eukaryotes (Fujinami et al., 1994), with ubiquitin itself varying by only two or three amino acids from plants to humans. Interestingly, ∼5% of the *Arabidopsis* genome encodes components of this pathway, more than 1300 of which are E3s (Vierstra, 2003), whereas there are “only” about 600 E3 genes known in humans (Li et al., 2008). The increased complexity of plants may stem from their sessile nature, which needs to cope with a considerable number of environmental stresses, such as temperature, weather, radiation, and chemical substances (Xu and Xue, 2019). In addition, the long plant cell cycle strongly requires targeted protein degradation, whereas fast-growing organisms like yeast can downregulate their protein levels via dilution through cell division.

Apart from cell homeostasis, the UPS is also involved in several systemic processes, especially plant immunity. Plants rely on a highly developed innate immune system to recognize pathogens and defend against pathogenic attacks. Therefore, the organism must be capable of mounting a strong and effective defense response while avoiding autoimmunity. The UPS is critical for the regulation of these processes through its involvement in oxidative bursts, hormone signaling, gene induction, and apoptosis (Trujillo and Shirasu, 2010).

Arguably, the most important component of the UPS is the 26S proteasome, which makes the final decision on protein fate and catalyzes protein degradation. It is a 1.7 MDa protein complex that acts as a multicatalytic ATP-dependent protease. The 20S core particle (CP) is given the proteolytic function and consists of four stacked heptameric rings. Thus, it has a barrel-like shape with a central cavity. The two inner rings are made up of seven different β subunits (termed PBA–PBG; see also Supplemental Table 1) (Fu et al., 1999) with three different protease active sites (PBA/β1, PBB/β2, PBE/β5) hidden within the inner chamber. The two outer rings are each composed of seven diverse α subunits (PAA–PAG) and control substrate entry via a gate. The gate opening is most commonly regulated by the 19S regulatory particle (RP) (Köhler et al., 2001), which can be bound to either one (26S proteasome) or both ends (30S proteasome) of the CP complex (Lander et al., 2012). It provides functionality for recognizing ubiquitinated proteins, unfolding and deubiquitination of the substrate, and threading of the unfolded polypeptides into the center of the CP for degradation. The RP can be further subdivided into a hexameric ring of RP AAA-ATPase subunits (AAA stands for ATPase associated with various cellular activities) (RPT1–6) and 13 non-ATPase subunits (RPN1–3, RPN5–13, and RPN15) (Finley et al., 1998). RPN1, RPN10, and RPN13 recognize polyubiquitin chains (Shi et al., 2016). RPN11 has a deubiquitination activity that can remove ubiquitin moieties bound to target proteins during their breakdown. Unfolding and threading of the substrate are catalyzed by the AAA-ATPase (Bard et al., 2018).

To fulfill all these functions, the RP samples a complex conformational landscape. Two conformations were initially identified, a substrate-free (S_A_; resting state) and a substrate-processing (S_C_) state, including a lid rotation of 30° relative to the base (Bard et al., 2018). Other intermediate states (S_B_ and S_D_) have been identified that differ mainly in the degree of lid rotation (Dong et al., 2019). With progress in cryoelectron microscopy (cryo-EM) technology, further subclassifications of proteasome states have been identified that provide the work cycle of the ATPase (Raab et al., 2009; Eisele et al., 2018; Ding et al., 2019).

## Results

**Figure 1:**
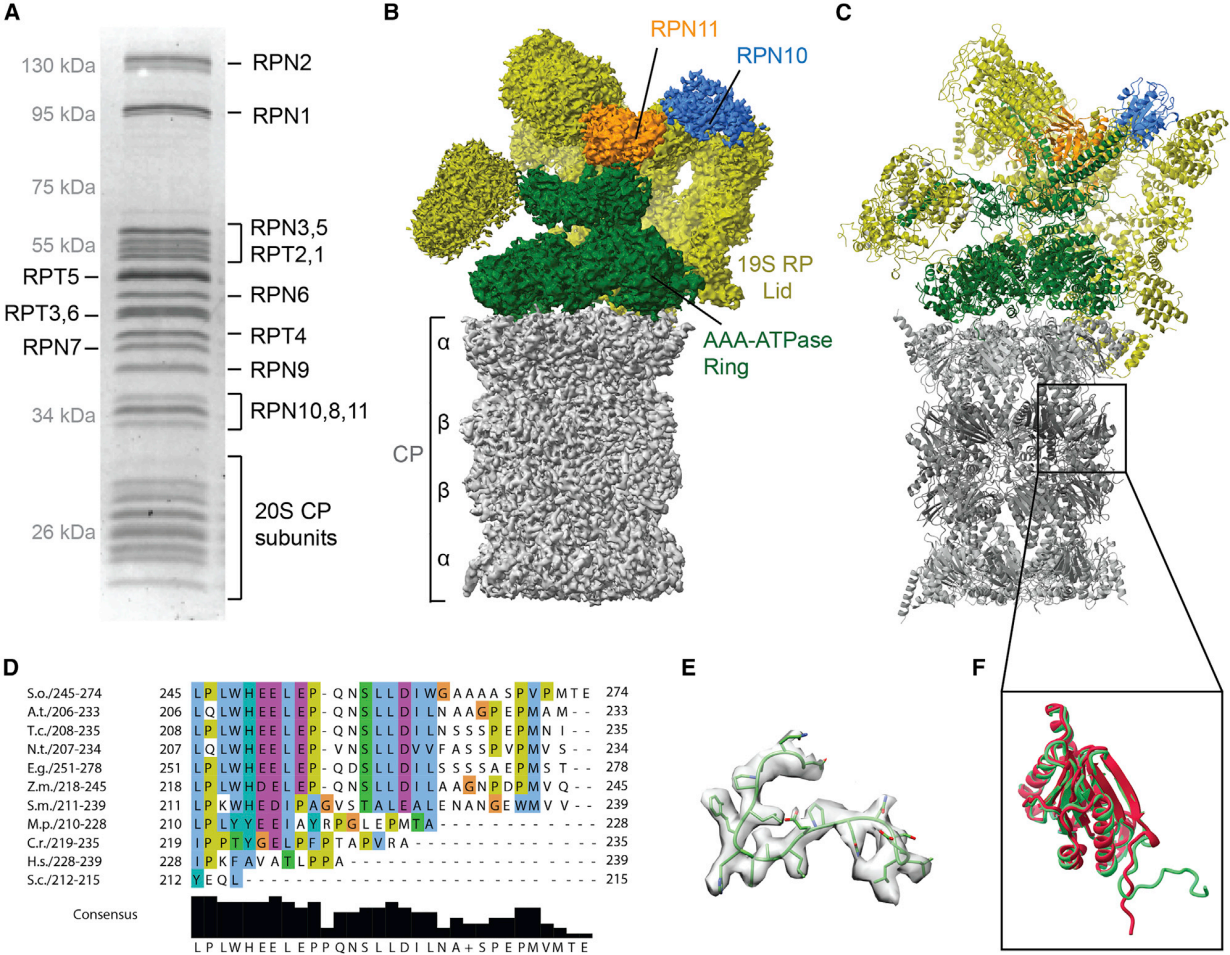
SDS–PAGE, cryo-EM map, and structure of the 26S proteasome from *Spinacia oleracea*. **(A)** SDS–PAGE analysis of the spinach 26S proteasome. All subunits from the CP and the RP are visible as bands. **(B)** The cryo-EM density of the 26S spinach proteasome is shown with its 19S RP lid subcomplex displayed in yellow, the RP base subcomplex in green, and the 20S CP in gray. The ubiquitin receptor RPN10 is colored blue, and the deubiquitinating enzyme subunit RPN11 is colored orange. **(C)** For the atomic model, the same color code as in **(B)** was used. **(D)** Alignment of the β1 C-termini of different organisms showing the divergence of the tail in higher plants, budding yeast, and human. S.o., *S. oleracea*; A.t., *Arabidopsis thaliana*; T.c., *Theobroma cacao*; N.t., *Nicotiana tabacum*; E.g., *Elais guineensis*; Z.m., *Zea mays*; S.m., *Selaginella moellendorffii*; M.p., *Marchantia polymorpha*; C.r., *Chlamydomonas reinhardtii*; H.s., *Homo sapiens*; S.c., *Saccharomyces cerevisiae*. **(E)** β1 extension of the spinach proteasome from residue 205 overlaid with the density of the cryo-EM map. **(F)** Overlay of the spinach (light green) and human (cherry red) β1 subunits. The black box points to the position of the subunit in the structure in **(C)**.

Although we have acquired substantial knowledge of the yeast and human proteasomes (Bard et al., 2018), we have only a limited understanding of the plant enzyme. Given the known differences in proteasome regulation between yeast and human, we also expected to find diversity in the proteasome of plants. We thus set out to investigate the structure of the plant proteasome. We adapted an affinity purification protocol for the human proteasome (Besche and Goldberg, 2012) that uses the ubiquitin-like (Ubl) domain of Rad23b as a bait to obtain comparable results. In the plant system, the Ubl domain of Rad23a led to better binding and similar yields. Our preparations contained all 33 integral subunits (Figure 1A) and at least two proteasomal interacting proteins (ECM29 and PSMD5) (Table 1; see also the complete mass spectrometry data).

**Table 1:** List of identified proteasome-associated proteins.

Accession no. (UniProt)	Protein	Peptides	Function
A0A0K9QNC1	CDC48/p97/VCP	19	chaperone
A0A0K9RJU4	ECM29	35	adaptor and scaffolding protein
A0A0K9QH09	PI31/PSMF1	5	proteasome inhibitor
A0A0K9R7D3	PSMD10	5	chaperone
A0A0K9RBE5	PSMD5	22	chaperone
A0A0K9R5W5	ubiquitin	3	–
A0A0K9RZ84	UBP6/USP14/TGT	12	deubiquitinating enzyme (DUB)
A0A0K9RL07	UCH2/UCH37/UCHL5	9	deubiquitinating enzyme (DUB)
A0A0K9RY40	UPL1-like/HUWE1/TOM1	26	E3 ligase
A0A0K9QUV5	UPL2-like/HUWE1	93	E3 ligase

In the next step, we assessed the activity of spinach 26S proteasomes alongside the activity of human 26S proteasomes using polyubiquitinated human securin as a model substrate. Both spinach and human proteases efficiently deubiquitinated and degraded the model substrate, despite sequence differences in ubiquitin and securin (Supplemental Figure 1). Estimating from the band intensity, we obtained an overall turnover of ub_n_-securin∗ of about 1 min at 25°C, which is in line with published results (Peth et al., 2013; Bard et al., 2019).

After these quality-evaluation steps, we subjected our preparation to single-particle cryo-EM for structural investigation. We determined a 3.3 Å resolution map that enabled us to model 80% of the fragmented density (Figure 1B and 1C). Comparing their overall architectures, we found high similarity to the mammalian and budding yeast proteasomes (Unverdorben et al., 2014). By contrast, we found a significant difference in the functionally relevant β1 subunit (Figure 1E and 1F). Unlike previously solved proteasome structures, the C terminus of the β1 subunit has a 20 amino acid long tail that forms a new contact to the surface of β7. A similar C-terminal extension of β1 evolved independently in diverse taxonomic divisions, such as Euglenozoa, Nematoda, Chlorophyta, and Streptophyta. In higher plants, we even found a highly conserved tail (Figure 1), which may indicate a motif for an additional binding partner or act as a signal for modification.

### Structure analysis

**Figure 2:**
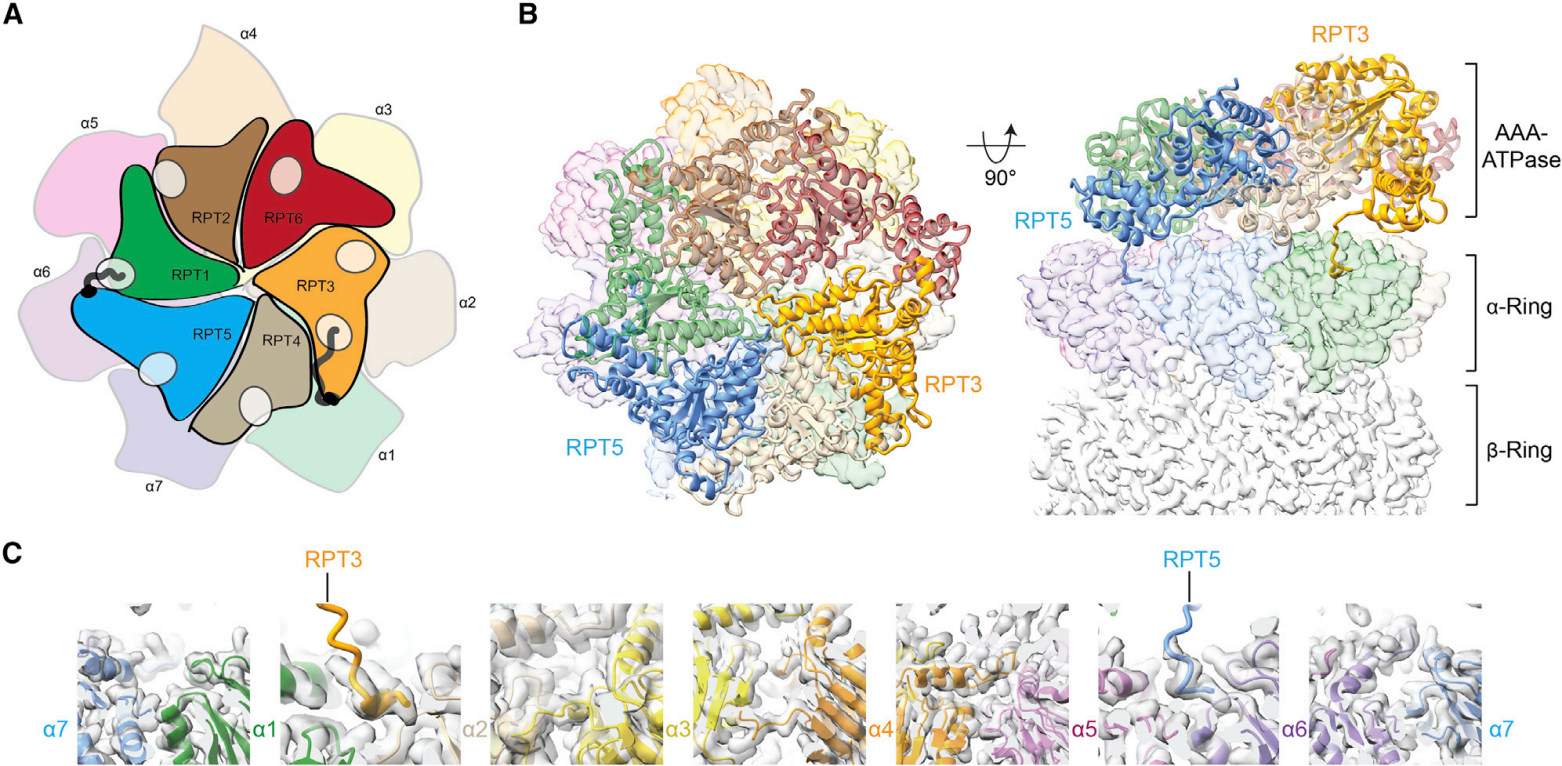
Interaction of two RPT N termini and their cognate α pockets in the resting state. **(A)** Cartoon depicting the interaction of two subunits of the ATPase ring (solid colors) and the α ring (opaque colors). The pockets of the α ring are shown as opaque ovals. The N terminus of RPT5 (solid blue) is associated with the α5–α6 pocket, and the HbYX motif of RPT3 (solid orange) is interacting with the α1–α2 pocket. **(B)** Tail–pocket interactions as determined by cryo-EM. UCSF Chimera software was used to visualize the interaction between the RPT3 and the RPT5 tails docked into their corresponding α pockets. For better orientation, the left shows the top view of the ATPase model and the α-ring density. The same color code was used as in **(A)**. The side view (right) shows the density of one β and α ring and the model of the AAA-ATPase, displaying the interaction of the N termini of RPT3 (solid orange) and RPT5 (solid blue) with the α ring. **(C)** Closer views of the α pockets. Although there is a clear density for the N terminus of RPT5 (light blue) in the α5–α6 pocket and the N terminus of RPT3 (dark orange) in the α1–α2 pocket, there is no density visible in the other α pockets.

Despite considerable sequence differences in a few functionally important subunits, we cannot detect further significant changes in the folds and conformations of the RP. Interestingly, the enzyme seems to resemble the human proteasome more than the yeast complex. For instance, RPN13 appears to be fully flexible and invisible in our structure while being present in our sample, similar to human preparations. The α-ring and the AAA-ATPase are well conserved, as shown in Supplemental Figure 2. Specifically, the ATPase is almost identical except for one significant difference in the C-terminal region of the RPT subunits, which contain the CP-interacting hydrophobic-tyrosine-X (HbYX) motifs that control the gate. Although all proteasomes in eukaryotes have clear HbYX motifs on the RPT2, RPT3, and RPT5 subunits, an additional HbYX motif is conserved in plants. In higher plants, the three C-terminal residues of RPT1 are consistent with an HbYX motif (VYN in spinach), whereas in other organisms the degree of hydrophobicity in the third-to-last amino acid varies. In humans, this position is occupied by threonine, which is at least partially hydrophobic; in yeast, this position is taken by the hydrophilic glutamine (Supplemental Figure 3). This may indicate different activation mechanisms for the proteasomes of diverse organisms, as the interaction with this motif is important for 20S gate regulation (Opoku-Nsiah et al., 2021). Our refined map shows clear density inside the α1–α2 and α5–α6 pockets, suggesting the insertion of the C-termini of RPT3 and RPT5 (Figure 2). It has been observed that, while in the resting state, the HbYX tails of RPT3 and RPT5 are inserted into their cognate pockets in the CP α-ring. The insertion of the C-terminus of RPT2 in the α3–α4 pocket follows during the lid rotation and deubiquitination procedure. During the ATPase cycle, additional insertion of the C-termini of the pseudo HbYX RPT1 and RPT6 into the lysine pockets at the interface of α2–α3 and α4–α5 completes gate opening and facilitates translocation. RPT3 does not promote gate opening but is instead more important for assembly of the 26S proteasome (Dong et al., 2019).

### Conformational variability

To further compare the plant proteasome architecture to known structures, we analyzed the dataset for conformational variability of the 26S proteasome (Supplemental Figures 4 and 5). The main conformation of the protein is a presumably inactive conformational state, which is characterized by a misalignment of the AAA-subcomplex pores and the 20S gate (Bard et al., 2018). This so-called S_A_ state is also found in our high-resolution structure. We also found all previously described non-S_A_ states. These have varying degrees of rotation between the lid and the base of the RP and different tilt angles between the two subcomplexes. Because of the continuous nature of these non-S_A_ states, we could not classify them into discrete states and instead described them as a continuum and classified the different conformational modes.

**Figure 3:**
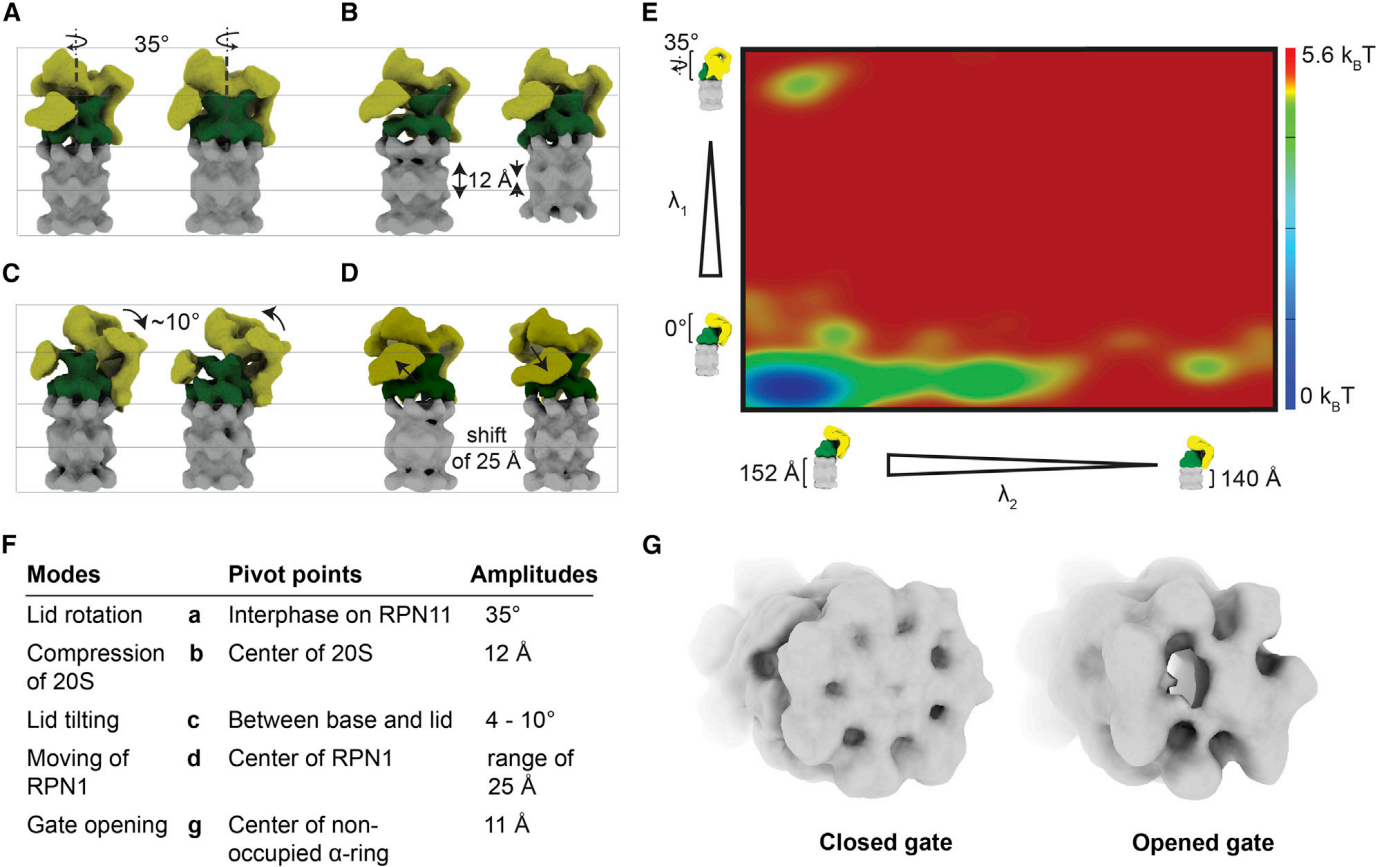
Most abundant trajectories of the spinach proteasome and energy landscape of the two most common conformations. **(A–D)** Trajectories of the 26S proteasome generated via PCA (CowSuite). **(A)** shows the well-characterized lid rotation, **(B)** the newly observed compression movement. Tilting of the lid can be observed in **(C)**, and **(D)** shows the movement of RPN1, which can range between 1 and 25 Å. **(E)** Conformational landscape of the spinach proteasome. The two most significant conformational modes are plotted as an energy landscape. The *y* axis denotes the rotation of the lid against the CP. The *xy* axis shows the newly described compression movement of the CP. Particle populations have been converted to energies by the Boltzman equation. **(F)** List of modes found in the dataset, their pivot points, and amplitudes. **(G)** Display of the opened gate on the unoccupied α-ring.

Our analysis found 58% of the particles in the resting state and 42% in non-S_A_ states. As in all other species, the most significant movement is the clockwise rotation of the lid in relation to the ATPase with a pivot point close to RPN11. Consequently, at a full rotation of 35°, RPN1 touches the coiled-coil formed by RPT4 and RPT5, and the entire ATPase is shifted to align the CP gate and the ATPase central channel. Second, we found a tilting (range 4°–10°) of the entire lid toward the ATPase with a pivot point in the free space between the ATPase and the lid, bringing the catalytic center of the deubiquitinating subunit RPN11 close to the entry point of the ATPase. Independent of the significant movements, we observed the free movement of RPN1, which can swing by 25 Å and rotate (Figure 3A–3D), similar to previously described movements of this subunit (Ding et al., 2019).

To our surprise, apart from the well-described conformations, we found an additional state that changes the CP rather than the RP (Supplemental Video 1A). A small yet significant subset of particles (7%) showed a 20S particle compressed by 12 Å that has less density on the non-RP-occupied gate (Figure 3). This flexibility of the free α-ring is reflected even in our high-resolution structure, where this α-ring is less resolved than the other parts of the structure (Supplemental Figure 5). We then constructed a conformational landscape using our previously published method (Haselbach et al., 2017, 2018). Analyzing the established landscape (Chen et al., 2016), we found the compression movement part of a pronounced continuous movement in which only the final fully compressed state is populated (Figure 3E). Surprisingly, in contrast to reports on the human and yeast conformational landscapes (Wu et al., 2020), the lid rotation is a less pronounced and sampled movement, showing only a few intermediate states. We found a considerable energy barrier of at least 5.6 k_B_T between the extreme states in both cases.

Supplemental Video 1. Movies of all mentioned states (A) Different modes from all used tools (Relion, CowSuite, cryoSPARC, PCA, cryoDRGN).

To confirm that this new movement is not an artifact of our analysis method, we turned to two additional tools to validate our observation. These tools use very distinct approaches, either a common-line-based principal-component analysis (PCA) or a variational autoencoder-based method. All procedures found the sparsely populated states that showed an extra opening of the unoccupied side but with a less pronounced compression. This finding is unexpected, as the gate is thought to be closed at all times (Groll et al., 2000; Smith et al., 2007) to protect from unwanted degradation. One possible explanation could be that this is a quick burst movement to open the gate, leading to the release of the peptide product.

To understand the significance of this movement, we analyzed the proteasomes of other organisms. A similar movement was described previously for the archaeal proteasome of *Thermoplasma acidophilum* (T20S) (Punjani and Fleet, 2021). Examining published datasets of the human proteasome, we could not identify similar movements, which may reflect the low occupancy of this state. Conversely, analyzing bovine proteasomes with negative stain, we found a low population of class averages showing compressed 20S particles (Supplemental Video 1B), indicating that this movement may also exist in mammalian proteasomes. We speculate that this motion may be required for the release of peptide products. This function has been loosely attributed to the pores at the surface of the 20S. However, no study has performed further analysis to support this claim.

Supplemental Video 1. Movies of all mentioned states (B) 2D classes of the bovine 26S proteasome combined in a video to show the compression.

### Proteasomal pores

**Figure 4:**
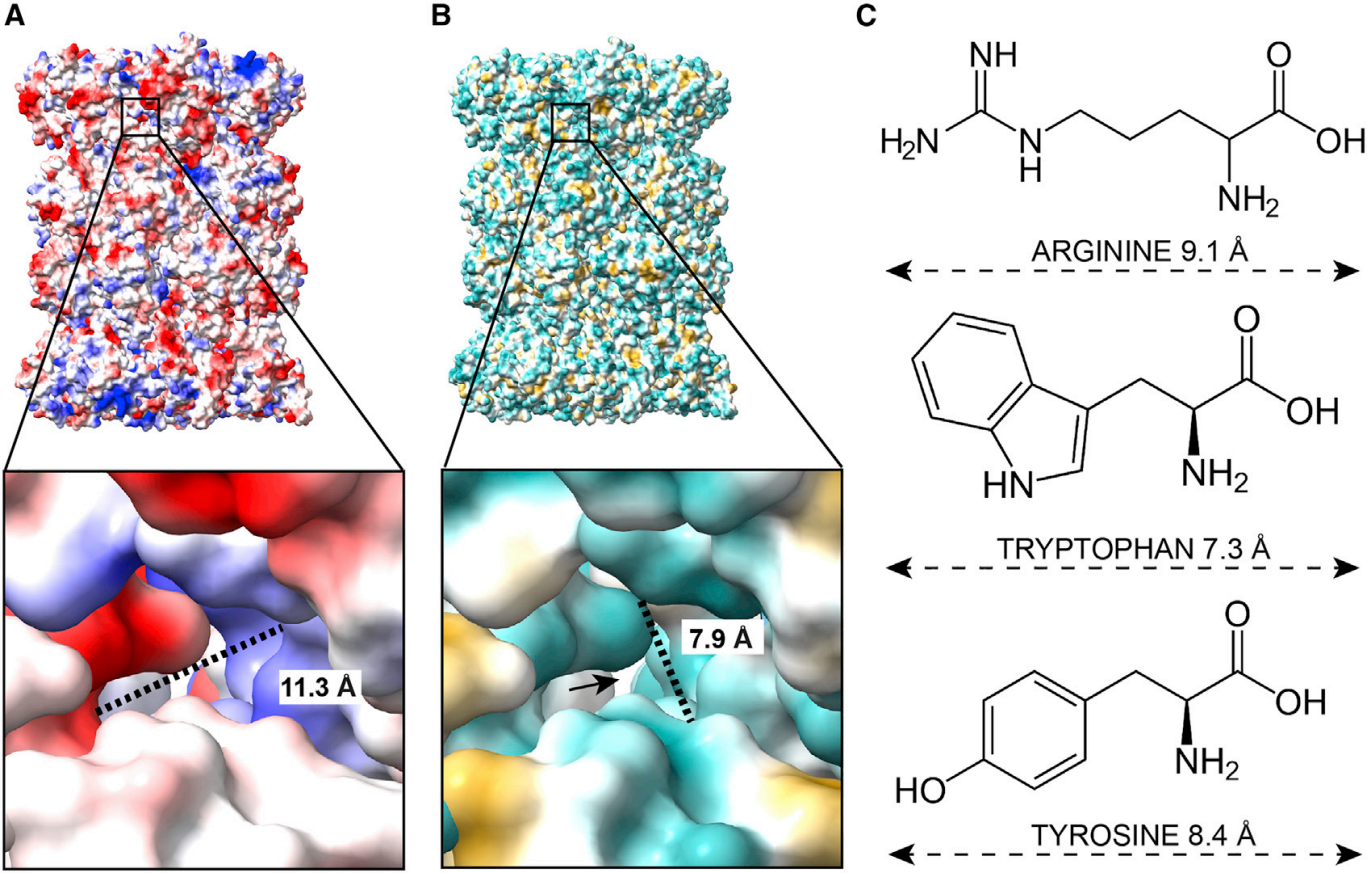
20S proteasome pores. **(A)** Electrostatic surface view of the 20S proteasome from *S. oleracea*. The pore shown measures 11.3 Å at its longest axis. Blue represents the positive and red the negative potential. **(B)** Hydrophobic surface view of the same position as in **(A)**. Pore size in the smallest axis was measured to 7.9 Å. The arrow points to another pore on the opposite side of the holoenzyme. Here, the pore shown is a hydrophilic (green) rather than a hydrophobic (yellow) pore. **(C)** Structural formula and size of the amino acids arginine, tryptophan, and tyrosine.

We measured the diameters of potential pores of the plant, human, and yeast proteasomes (Figure 4). We found a total of 30 pores in spinach, 25 in human, and 12 in yeast, ranging in diameter from 4 to 17 Å (Supplemental Figure 6). By contrast, the opened gate in these species had a diameter of 14 Å. The largest transverse diameter of a peptide is given by the bulky amino acids (arginine, tryptophan, and tyrosine), suggesting that pores smaller than this will not be sufficient to release the products of a substrate. Only larger pores (>7 Å) that can let the bulky amino acids through can be considered for peptide release. Judging from the size of the pores alone, in principle, there are adequately sized pores in all proteasomes that would allow for the release of products. However, the question remains whether this is sufficient for the kinetics of the proteasome. The protease would need to release its products at least as fast as it translocates the substrate into the chamber. As these pores are only twice as big as the product diameter in the largest case, it is not clear that they would be sufficient for a fast release of peptides.

To estimate this, we followed a model calculation for diffusion through narrow pores (Sung and Park, 1996). Assuming that the pores are inert, peptide diffusion occurs as described by the formula $∼\frac{L^{2}}{2D}$, where *L* is the length of the peptide and *D* is its diffusivity. This analysis demonstrated that short peptides (*L* = 2) would diffuse through the pore four times slower, whereas long ones (*L* = 10) would move 100 times slower than their free diffusion in solution. The diffusion constant of a typical proteasome product has been estimated to be about 3 μm^2^/s (Reits et al., 2003), meaning that it would diffuse 1 nm (thickness of the pore) in 160 ns in solution and in 16 μs through the pore. To put this in context, the proteasome degrades a protein at a speed of 40 amino acids per second (Luciani et al., 2005; Sha et al., 2018). This means that the translocation speed is still four orders of magnitude slower than the possible release through the pores; thus, it is possible that these pores suffice. However, if the pore is not inert, meaning that the peptide is attracted to the inside of the pore, a massive slowdown would occur. Given the distribution of charge and hydrophobicity in the identified pores, we must assume that there is at least some interaction potential, which, however, we cannot quantify. In summary, the stochastic opening of the gate may facilitate peptide release but may not be strictly required.

## Discussion

The plant proteasome has been intensely characterized biochemically; however, structural insights have been lacking (Ozaki et al., 1992; Fujinami et al., 1994; Yang et al., 2004; Book et al., 2010).

Here, we present the first 3D structure of the plant 26S proteasome. Despite billions of years of evolutionary divergence, we could find only two significant differences between the proteasomes of higher plants and higher animals, highlighting the importance of this machine for all life. The main difference between plants and animals occurs at the C-terminus of the β1 subunit. Its high conservation in higher plants, as well as its evolutionary reinvention in several other organism groups, indicates its significance. As we could not find a change in the basal activity of the proteasome, we can only hypothesize that this well-structured extension may serve as a new binding or modification site.

Although we could resolve the 20S CP well, the resolution was limited in the 19S region. We speculate that this limitation is due to isoforms present in the preparation. Whereas yeast and human assemble a single 26S proteasome complex from a unigene set of RP and CP genes, most subunits of the plant protein are encoded by two genes. Mass spectrometry data (Supplemental Figure 7 and Supplemental Table 1) suggest that the proteasome is not a single particle, but a heterogeneous collection assembled using both paralogs of duplicated CP and RP subunits. Some of these pairs are nearly identical; others display enough divergence (e.g., RPN12) to suggest that different activities are possible (Yang et al., 2004; Book et al., 2010; Gemperline et al., 2019).

Our investigation of the complex dynamics showed the already known human and yeast conformations, such as the rotation and tilting of the lid and the movements of RPN1. We found these to be highly conserved; however, despite similar preparations, we did not successfully define individual sets in our dataset but rather a continuum of movements, indicating different energetics.

In addition, we could identify a compression movement of the CP, in combination with a partially opened gate on the RP-distal α-ring. The gate opening and the overall more flexible RP may be consequences of a complex mechanism that plants require to cope with extreme environmental conditions, like temperature fluctuations.

We speculate that the observed stochastic gate opening may serve as an additional peptide release mechanism. How degradation products are released by the proteasome is still a mystery that has barely been investigated. A general suggestion in the field is the existence of pores in the 20S CP that suffice for peptide exit. Our findings suggest that pores do exist and are theoretically capable of releasing peptides. Assuming that this mechanism is the only way for peptides to exit the holoenzyme, the chemical nature of the pores suggests that this release step may be rate limiting, at least for some peptides. Therefore, the stochastic gate opening would be an elegant way to solve the issue and overcome the rate-limiting factor of the pores. Future studies are needed to characterize the phenomenon of peptide release from proteasome pores, which could be a detrimental part of the proteolytic capacities of the proteasome in general.

## Methods

### Purification of 26S proteasomes from *Spinacia oleracea*

The original protocol described by Besche and Goldberg (2012) and Marshall et al. (2017) was slightly modified for the purification of 26S proteasomes from *S. oleracea*. Fresh spinach leaves ("Simply good" young spinach from the supermarket "Billa" in Austria) were frozen in liquid nitrogen and ground using a freezer/mill (SPEX SamplePrep). The following steps for the purification procedure were performed at 4°C on ice or in a cold room. The powder was resuspended at 2 g (fresh weight)/ml extraction buffer (EB; 25 mM Bis–Tris [pH 6.5], 50 mM KCl, 5 mM MgCl_2_) freshly supplemented with 10% (w/v) glycerol, 20 mM ATP, 5 mM DTT, 5% (w/v) polyvinylpolypyrrolidone, and 2 mM phenylmethylsulfonyl fluoride. The debris was filtered through a stack of four layers of cheesecloth (Regency Naturals, Dallas, TX, USA) and two layers of Miracloth (Merck Millipore) and clarified by two ultracentrifugation steps, initially for 10 min at 30 000 *g* followed by 30 min at 100 000 *g* (Optima XE-90 ultracentrifuge, rotor 45TI, Beckman Coulter). The 26S proteasome was further purified by fractionated precipitation. Contaminating proteins were removed by gentle addition of polyethylene glycol 8000 (50% [w/v] stock [in EB] supplemented with 10 mM ATP) to a final concentration of 5% (v/v) under constant stirring and incubation for 15 min. Precipitated proteins were removed by centrifugation at 16 000 *g* for 15 min (Sorvall Lynx 6000 centrifuge, Thermo Scientific), and the desired protein was precipitated by increasing the polyethylene glycol concentration to 20% (v/v). Precipitated 26S proteasome was pelleted by centrifugation at 12 000 *g* for 15 min and dissolved in EB supplemented with 5% (w/v) glycerol, 10 mM ATP, and 5 mM DTT.

Approximately 3 mg of Ubl domain from Rad23a fused to GST (GST-Ubl, see below) was immobilized on 1 ml magnetic GST beads (MagneGST particles, Promega) equilibrated with EB. After overnight incubation at 4°C with dissolved proteasomes on a rotary wheel, the proteasome–Rad23a–Ubl–GST complex was eluted from the beads by the addition of 25 mM reduced glutathione (Roth). Finally, GST-Ubl was removed from the pure 26S proteasomes by sucrose gradient ultracentrifugation (10%–30% [w/v] sucrose in EB supplemented with 10 mM ATP and 5 mM DTT) in SW60 tubes (Seton) using a swinging bucket rotor (SW 60 Ti, Beckman Coulter). After centrifugation at 88 000 *g* (28 000 rpm) for 16 h, the gradient was manually fractionated to 200-μl aliquots, and final protein quality was monitored using SDS–PAGE and negative staining EM.

If not otherwise specified, the chemicals were provided by Sigma Aldrich.

### Purification of HBTH-tagged Rpn11 26S proteasomes from HEK293 GP cells

Frozen cells in resuspension buffer (25 mM Bis–Tris [pH 6.5], 50 mM KCl, 5 mM MgCl_2_, 0.1% NP-40, 10% glycerol) were thawed in a water bath at 37°C after addition of 4 mM ATP, 1 mM DTT, 0.1 mM phenylmethylsulfonyl fluoride, and a 2500× dilution of benzonase. After the cells were lysed with a Dounce homogenizer, they were centrifuged at 19 000 rpm for 45 min at 4°C (Sorvall Lynx 6000 centrifuge, Thermo Scientific). The supernatant was then ultracentrifuged for 1 h at 100 000 *g* and 4°C (Optima XE-90 ultracentrifuge, Beckman Coulter). After filtration through a double layer of Miracloth (Merck Millipore) followed by a 0.45-μm filter, a HiTrap streptavidin 1-ml column (GE Healthcare) and a GST Trap FF 1-ml column (GE Healthcare) were used for purification. The proteasome complexes were bound to the streptavidin column equilibrated with buffer A (EB with 10% [w/v] glycerol, 1 mM DTT, and 4 mM ATP). The bound proteins were first washed with buffer A supplemented with 150 mM NaCl and cleaved off from the column by application of GST-TEV protease and incubation overnight at 4°C. The proteasomes were then eluted with 1× EB with freshly added 10% (w/v) glycerol, 0.5 mM Tris(2-carboxyl)phosphine (TCEP), and 4 mM ATP. The fractions containing purified 26S proteasomes were identified via SDS–PAGE.

If not otherwise specified, the chemicals were provided by Sigma Aldrich.

### Cloning and purification of Rad23a-Ubl-GST

Cloning and purification of GST-Ubl were performed according to standard procedures. The codon region spanning the Ubl domain of Rad23a (residues 1–88) was amplified from a synthetic gene (Thermo Fisher GeneArt) and cloned into the pGEX vector by Gibson assembly. The plasmid encoding GST-Ubl was transformed into *Escherichia coli* strain BL21(DE3). Overnight cultures were inoculated with single colonies, cells were grown in autoinduction medium (ZY medium [tryptone, yeast extract, and ddH_2_O] with 20× P [100 mM PO_4_, 25 mM (NH_4_)_2_SO_4_], 50× 5052 [0.5% glycerol, 0.05% glucose, 0.2% α-lactose], 0.001 mM MgSO_2_, and antibiotics) at 37°C for 5.5 h and subsequently cooled to 18°C, and growth resumed overnight. Bacteria were harvested by centrifugation, and the pellet was resuspended in 1× phosphate-buffered saline (PBS; KCl, KH_2_PO_4_, NaCl, Na_2_HPO_4_, 10 mM MgCl_2_, 2 mM DTT, and 1 μl benzonase). Cell walls were broken with a cell disrupter (Constant Systems, UK), and cell debris was cleared by centrifugation. The supernatant was loaded onto a 5-ml GST trap column (GE Healthcare) equilibrated with 1× PBS supplemented with 2 mM DTT. Unbound proteins were removed by intensive washing with 1× PBS, and GST-Ubl was eluted with 1× PBS supplemented with 2 mM DTT and 10 mM reduced glutathione. Finally, the protein was polished by size exclusion using HiLoad 16/600 Superdex 75 pg (Sigma Aldrich) equilibrated with 1× PBS. All steps were monitored by SDS–PAGE, and the protein was concentrated to 5 mg/ml, frozen in liquid nitrogen, and stored at −80°C.

If not otherwise specified, the chemicals were provided by Sigma Aldrich.

### Substrate production and purification

Full length securin∗ was expressed in BL21(DE3) Codon Plus (RIL) cells, purified, and fluorescently labeled (denoted by an asterisk) as previously described (Jarvis et al., 2016). Polyubiquitinated securin was generated enzymatically by mixing 10 μM substrate, 0.1 μM APC/C, 1 μM CDH1, 5 μM UBCH10, 1 μM E1 (Uba1), 100 μM ubiquitin, and 10 mM Mg-ATP. After 1.5 h at room temperature, the reactions were quenched with 50 mM EDTA (pH 8.0) and flash frozen.

To purify the substrate from the ubiquitin ligase machinery for the activity assay, 1.6 μl NaAc (pH 4.0) was added and incubated on ice for 30 min. After centrifugation, the supernatant was carefully taken without disrupting the pellet, and the pellet was then dissolved in 1× PBS and dialyzed in 1× EB with 10% glycerol.

### *In vitro* degradation assay

Proteasomal activity was characterized using ub_n_-securin∗. Freshly purified plant proteasomes (30 nM) were incubated with 10-fold (300 nM) substrate protein in 200 μl buffer (25 mM Tris [pH 7.5], 5 mM MgCl_2_, 5% glycerol [w/v]) freshly supplemented with 5 mM ATP and 1 mM DTT at 25°C under constant agitation for 20 h. Aliquots were withdrawn after 0, 10, 30, 60, 90, 120, and 180 min and 20 h of incubation. The reaction was quenched by denaturation using SDS sample buffer (final 1×), separated by stain-free SDS–PAGE (Criterion TGX stain-free precast gel 4%–20%, 26 wells, Bio Rad; running conditions 180 V for 40 min) and imaged using a ChemiDoc MP imaging system (Bio-Rad). Because of the fluorescently labeled substrate securin, deubiquitination and degradation by the proteasome could be visualized using the fluorescein channel of the ChemiDoc system. As a control, 2 mM MG132 (a proteasome inhibitor that blocks the proteolytic activity of the proteasome; MedChemExpress) was used to show the inhibited proteasome. The same reactions were also performed with human proteasomes (purified from HEK293 cells) at 37°C.

Figures showing the degradation assay (Supplemental Figure 1) were edited to remove scratches from the scanner surface in the background using the Spot Healing Brush Tool in Photoshop (2022).

### Mass spectrometry

#### Nano-LC–MS analysis

The nano-high performance liquid chromatography system used was an UltiMate 3000 RSLCnano system (Thermo Fisher Scientific, Amsterdam, the Netherlands) coupled to a Q Exactive HF mass spectrometer (Thermo Fisher Scientific, Bremen, Germany) equipped with a Proxeon nanospray source (Thermo Fisher Scientific, Odense, Denmark). Peptides were loaded onto a trap column (Thermo Fisher Scientific, Amsterdam, the Netherlands; PepMap C18, 5 mm × 300 μm i.d., 5-μm particles, 100-Å pore size) at a flow rate of 25 μl/min using 0.1% Trifluoroacetic acid (TFA) as the mobile phase. After 10 min, the trap column was switched in line with the analytical column (Thermo Fisher Scientific, Amsterdam, the Netherlands; PepMap C18, 500 mm × 75 μm i.d., 2 μm, 100 Å). Peptides were eluted using a flow rate of 230 nl/min and a binary 4-h gradient, respectively 260 min.

The gradient began with the mobile phase 98% A (water/formic acid, 99.9/0.1, v/v) and 2% B (water/acetonitrile/formic acid, 19.92/80/0.08, v/v/v), increased to 35% B over the next 240 min, increased to 90% B over 5 min, remained there for 5 min, and decreased back to 98% A and 2% B in 5 min for equilibration at 30°C.

The Q Exactive HF mass spectrometer was operated in data-dependent mode, using a full scan (*m/z* range 380–1500, nominal resolution of 60 000, target value 1 × 10^6^) followed by tandem mass spectrometry (MS/MS) scans of the 10 most abundant ions. MS/MS spectra were acquired using a normalized collision energy of 27%, isolation width of 1.4 *m/z*, and resolution of 30 000, and the target value was set to 1 × 10^5^. Precursor ions selected for fragmentation (excluding charge states 1, 7, 8, >8) were put on a dynamic exclusion list for 60 s. In addition, the minimum AGC target was set to 5 × 10^3^, and the intensity threshold was calculated to be 4.8 × 10^4^. The peptide match feature was set to preferred, and the exclude isotopes feature was enabled.

#### Data processing protocol

For peptide identification, the RAW files were loaded into Proteome Discoverer (version 2.1.0.81; Thermo Scientific). All created MS/MS spectra were searched using MS Amanda v.2.0.0.9849, Engine v.2.0.0.9849 (Dorfer et al., 2014). The RAW files were searched against the *Arabidopsis* genome database TAIR (33 038 sequences; 14 616 625 residues) and the UniProt database using the taxonomy *S. oleracea* (23 985 sequences; 9 454 539 residues). The following search parameters were used: iodoacetamide derivative on cysteine was set as a fixed modification, and oxidation on methionine, deamidation on asparagine and glutamine, acetylation on lysine, phosphorylation on serine, and threonine and tyrosine were set as variable modifications. Monoisotopic masses were searched within unrestricted protein masses for tryptic enzymatic specificity. The peptide mass tolerance was set to ±5 ppm and the fragment mass tolerance to ±15 ppm. The maximum number of missed cleavages was set to 2. The result was filtered to 1% false discovery rate on protein level using the Percolator algorithm (Käll et al., 2007) as integrated in Proteome Discoverer. The localization of the post-translational modification sites within the peptides was performed with the ptmRS tool based on the phosphoRS tool (Taus et al., 2011). Peptide areas were quantified using the in-house-developed tool apQuant (Doblmann et al., 2018).

### Bioinformatics: Sequence retrieval

To collect spinach orthologs, we performed NCBI blast searches using sequences of the *Arabidopsis thaliana*, *Saccharomyces cerevisiae*, and *Homo sapiens* proteasomes against a set of *S. oleracea* proteins that were downloaded from NCBI (58 427 entries, status 04/2018) (Altschul et al., 1997). Hits were selected for the lowest E value and aligned with MAFFT (Katoh and Toh, 2008). Paralogous gene families, such as the α or β core proteasome subunits or the ATPase regulatory subunits RPT1 to RPT6, were aligned, including all paralogs. The respective orthologs were assigned in an neighbour-joining (NJ) phylogenetic tree with SeaView (Gouy et al., 2010). To study the C-terminal conservation of RPT1 to RPT6, we extracted full-length orthologs from the NCBI or UniProt sequence databases, aligned them with MAFFT, and visualized the alignment with Jalview (Waterhouse et al., 2009). Putative sequence fragments were excluded. For a graphical representation of the alignments, residues were colored using the Clustal X coloring scheme.

### Negative staining

Four microliters of the sample was applied to a carbon-coated grid and incubated for 30 s to 1 min, depending on the concentration of the sample. The grid was blotted, washed two times with ddH_2_O, and stained with uranyl acetate for 1 min (Hoppert, 2003). Samples were imaged on an FEI Technai T20 microscope at a magnification of 60 000×, corresponding to a pixel size of 1.85 Å per pixel with an Eagle 4k HS camera.

### Preparation of cryo grids

A GraFix gradient (Stark, 2010) was used to stabilize the complex for single-particle cryo-EM. A 10%–30% sucrose gradient was prepared as described previously with 0.05% (v/v) glutaraldehyde added to the 30% sucrose buffer and quenched with 5 mM aspartate (pH 7.3) during fractionation. The peak fractions containing the 26S proteasomes were identified by SDS–PAGE and negative staining EM. Sucrose was removed from the sample prior to vitrification by buffer exchange (Zeba Spin Desalting columns, Thermo Scientific). The particles were subsequently absorbed to a continuous carbon film attached to a Quantifoil (3.5/1) 200 mesh grid and plunge frozen with a Leica EM GP.

### Electron cryo microscopy data acquisition

The cryo grid was imaged in a 300-kV FEI Titan Krios transmission electron microscope, and images were taken at a nominal magnification of 75 000, resulting in a pixel size of 1.058 Å per pixel on a Falcon 3D detector. Two datasets were collected with a total dose of 80 electrons/A^2^, and the total dose of the third dataset was fractionated on 50 electrons/A^2^. In total, 22 858 micrographs were collected.

### Data processing

Image frames were aligned and weighted according to electron dose using the software MotionCor2 (Zheng, 2016), followed by contrast transfer function (CTF) determination using gCTF (Zhang, 2016). The micrographs were sorted using CowSuite’s Quality Checker (unpublished results), and bad micrographs (blurred, contaminated, or empty) were discarded. Two thousand three hundred eleven particles were manually picked on the left 10 027 micrographs using Relion (Zivanov et al., 2019). With the class averages from Relion, Gautomatch (http://www.mrc-lmb.cam.ac.uk/kzhang/) was able to pick 1 779 876 particles. Through the 2D classification, the best classes could be chosen to generate an initial 3D model, using it as reference in a 3D classification in Relion. By alignment and comparison of 3D classifications in UCSF Chimera (Pettersen et al., 2004), it was possible to distinguish different conformations within the dataset. PCAs in CowSuite (https://www.cow-em.de), cryoDRGN (Zhong et al., 2021), and CryoSPARC2 (Punjani et al., 2017) were used as additional tools (Supplemental Tables 2 and 3).

### Model building

Homology models for the individual proteasomal chains were generated by CHAINSAW (a program for mutating PDB files used as templates in molecular replacement) (Stein, 2008) using the human 26S proteasome (6msb) model and sequence alignments produced by Clustal X (Jeanmougin et al., 1998). Non-conserved residues were pruned to their Cβ atom. The model for the human 26S proteasome was placed into the EM density using Chimera, followed by real-space rigid-body refinement as a single entity. The individual chains from homology modeling were placed by superposition in Coot (Emsley and Cowtan, 2004) and further corrected by individual rigid-body refinement of single polypeptide chains. Pruned side chains were corrected and placed manually, and an initial model was made by real-space refinement in Coot. The chains were further fitted to the map by real-space refinement using PHENIX (Liebschner et al., 2019), except those for which the map was ambiguous and did not permit further fitting (subunits of the lid).

For those subunits, we generated α fold models (Jumper et al., 2021) truncated to poly(Ala) using the PDB tool in PHENIX and then rigid-body fitted them into the map. Model quality was assessed using MolProbity (Williams et al., 2018). The final model proved to have good stereochemistry, with 93.81% and 94.94% residues (20S and 19S) in the favored region of the Ramachandran plot and 0.13% and 0.23% outliers. Figures were produced using ChimeraX (Pettersen et al., 2021) (Supplemental Tables 2 and 3).

## Funding

D.L.B. and N.G.B. are supported by 10.13039/100000002NIH R35GM128855 and the University Cancer Research Fund (UCRF). H.D.T. and B.F. were supported by 10.13039/100000002NIH grant R01GM125769. The IMP, the whole Haselbach lab, and especially S.K. are supported by 10.13039/100001003Boehringer Ingelheim.

## Author contributions

S.K. performed most of the experiments under the supervision of I.G. and guidance of D.H. I.G. executed the cloning and gave general biochemical support. K.B. helped to establish degradation assays for the plant system. S.A. provided help with the assays and substrates. A.M. supervised and supported model building. N.G.B. and D.L.B. performed ubiquitination assays and provided ubiquitinated substrates. A.S. implemented bioinformatic sequence analysis and annotated spinach subunits. K.M. and R.I. provided proteomic analysis. B.F., H.D.T., and E.D.Z. contributed to the analysis of conformational states. S.K. and D.H. wrote the manuscript with the support of all other authors.

## Accession numbers

The EM density map has been deposited in the EMDB with accession no. EMD-14175. RAW micrographs have been uploaded to the EMPIAR database (EMPIAR-10974). Modeled atomic coordinates have been deposited in the Protein Data Bank with accession nos. 7QVE (20S) and 7QVG (19S). The materials and data that support the findings of this study are available from the corresponding authors upon request.

## Supplemental information

Document S1. Supplemental Figures 1–7 and Supplemental Tables 1–3

Document S2. Article plus supplemental information
