# Supplementary material for: Cryo-EM structure of the plant 26S proteasome
Source: Plant Commun. 2022 Mar 11;3(3):100310. doi: 10.1016/j.xplc.2022.100310 (PMC9251434; doi:10.1016/j.xplc.2022.100310)
Supplement: Document S1. Supplemental Figures 1–7 and Supplemental Tables 1–3 [file mmc1.pdf]

Plant Communications, Volume *3*

Supplemental information

Cryo-EM structure of the plant 26S proteasome

Susanne Kandolf, Irina Grishkovskaya, Katarina Belačić, Derek L. Bolhuis, Sascha Amann, Brent Foster, Richard Imre, Karl Mechtler, Alexander Schleiffer, Hemant D. Tagare, Ellen D. Zhong, Anton Meinhart, Nicholas G. Brown, and David Haselbach

Supplemental information

Cryo-EM structure of the plant 26S proteasome

Susanne Kandolf, Irina Grishkovskaya, Katarina Belačić, Derek L. Bolhuis, Sascha Amann, Brent Foster, Richard Imre, Karl Mechtler, Alexander Schleiffer, Hemant Tagare, Ellen D. Zhong, Anton Meinhart, Nicholas G. Brown & David Haselbach

Supplemental Figures

Figure S1. SDS PAGE analysis of proteasomal degradation with polyubiquitinated human securin.

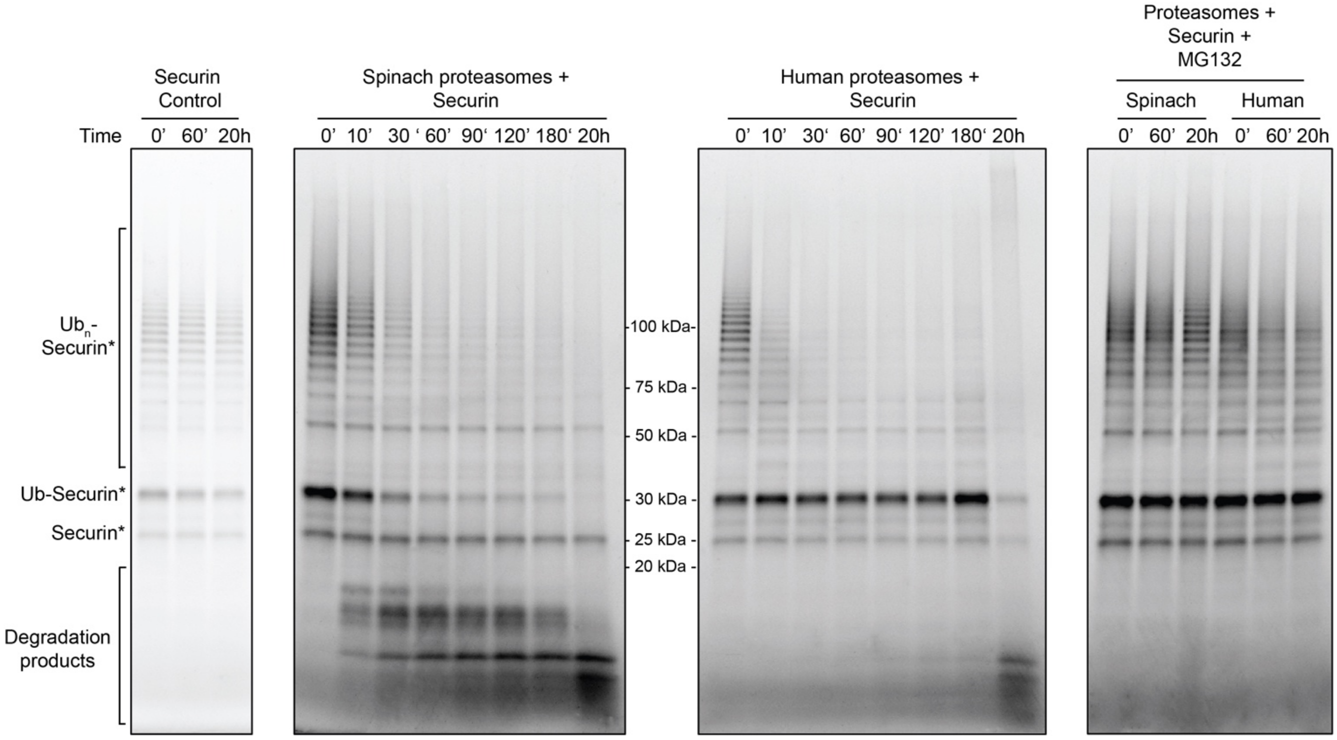

The bands ranging from 50-150 kDa represent securin with varying sizes of polyubiquitin chains (Ub_n-Securin*), the band corresponding to 30 kDa represents monoubiqutinated securin (Ub-Securin*) and the band corresponding to free securin is visualized at 25 kDa. The bands with sizes smaller than 25 kDa represent the degradation products. In reactions with spinach and human proteasomes, the disappearance of the Ub_n-Securin* bands points to the deubiquitination activities, while the appearance of degradation products confirms the degradation activity of the complexes. The presence of MG132 (proteasome inhibitor) abolishes degradation and coupled deubiquitination activities of both, spinach and human 26S proteasomes.

The image shows fluorescent signal derived from fluorescein maleimide labeled securin.

Figure S2. Overview of the subunit homology of *S. oleracea* compared to *A. thaliana*, *H. sapiens* und *S. cerevisiae.*

A

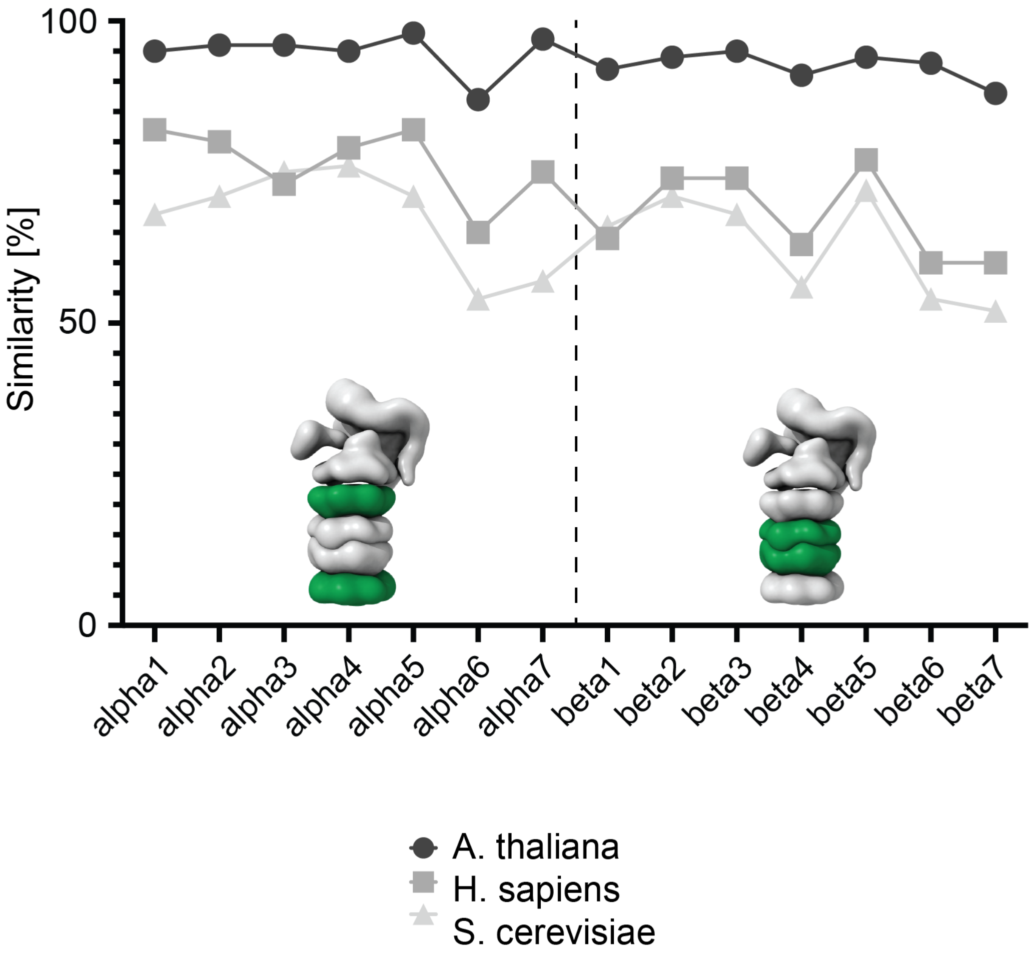

B

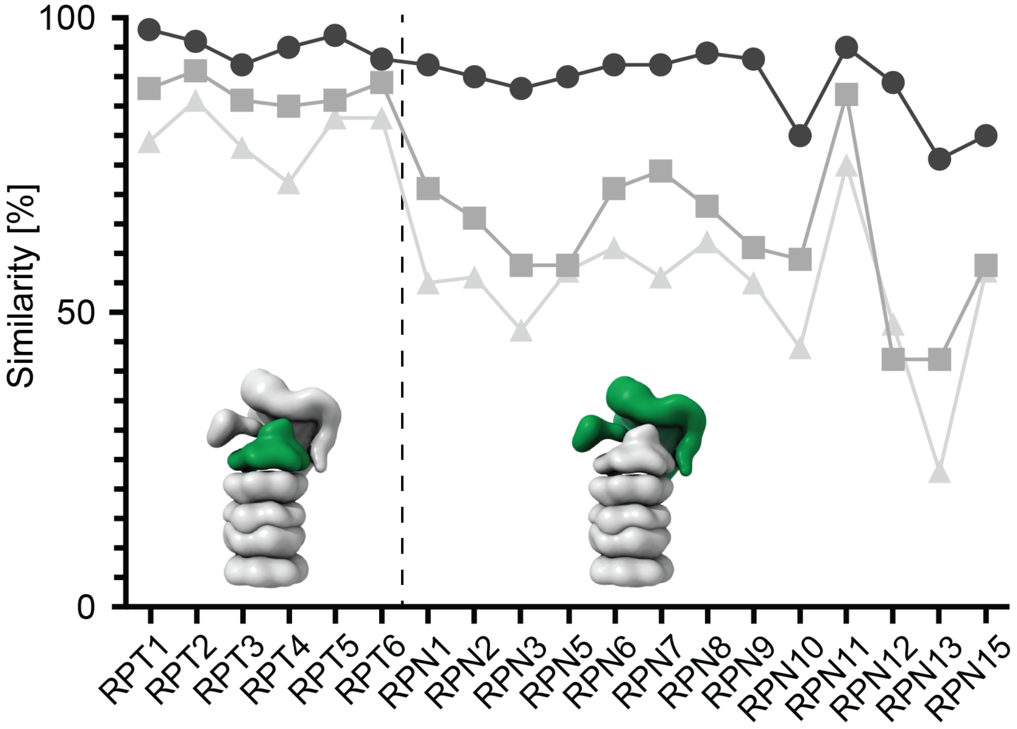

(A) Similarity of the ring subunits from spinach compared to the subunits of *A. thaliana*, mammalian, and budding yeast. As expected, the similarity of the 20S between spinach and *A. thaliana* lies above 90%, comparing spinach and human, it is roughly above 60%, and yeast is at 60%. **(B)** Lid and base subunit comparison of *A. thaliana, H. sapiens and S. cerevisiae*. The base of the proteasome seems quite conserved, while the lid differs significantly. Except the DUB RPN11, the similarity of the subunits of the lid lies at 80% for *A. thaliana*, at 60% for *H. sapiens* and at 50% for *S. cerevisiae*.

Figure S3. Derived amino acid sequence alignment - termini of HbYX motifs of the AAA-ATPase subunits compared to different organisms. Legend: *S.o. - Spinacia oleracea, A.t. - Arabidopsis thaliana, C.r. - Chlamydomonas reinhardtii, S.c. - Saccharomyces cerevisiae, D.m. - Drosophila melanogaster, H.s. - Homo sapiens.*

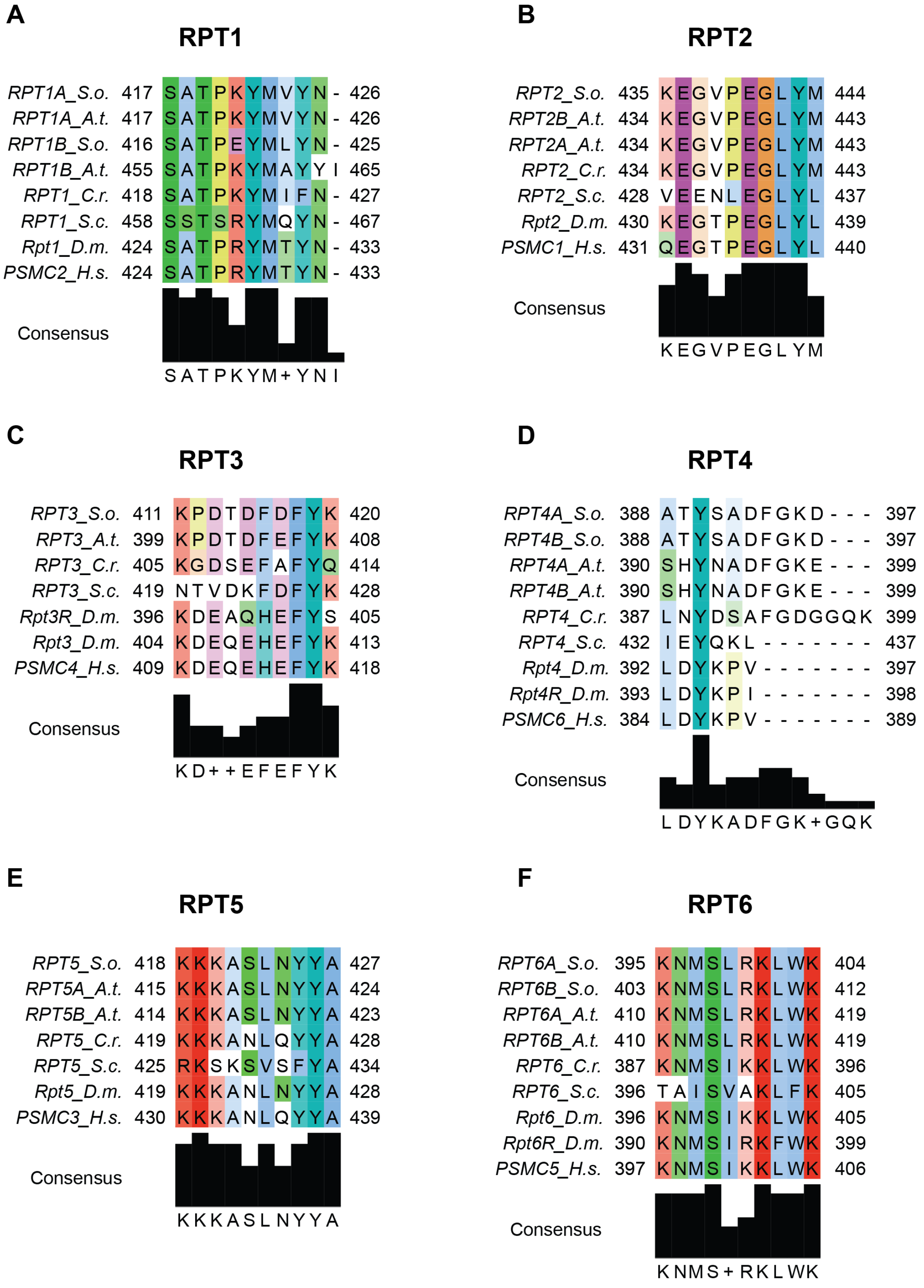

(A) – (F) Alignments of each RPT subunit C-terminus of the 26S proteasome. The sequence of subunits is relatively conserved, except for RPT1. Plants appear to have an additional HbYX motif - besides RPT2, 3 and 5 - compared to mammals.

Figure S4. Workflow of cryo-EM data processing.

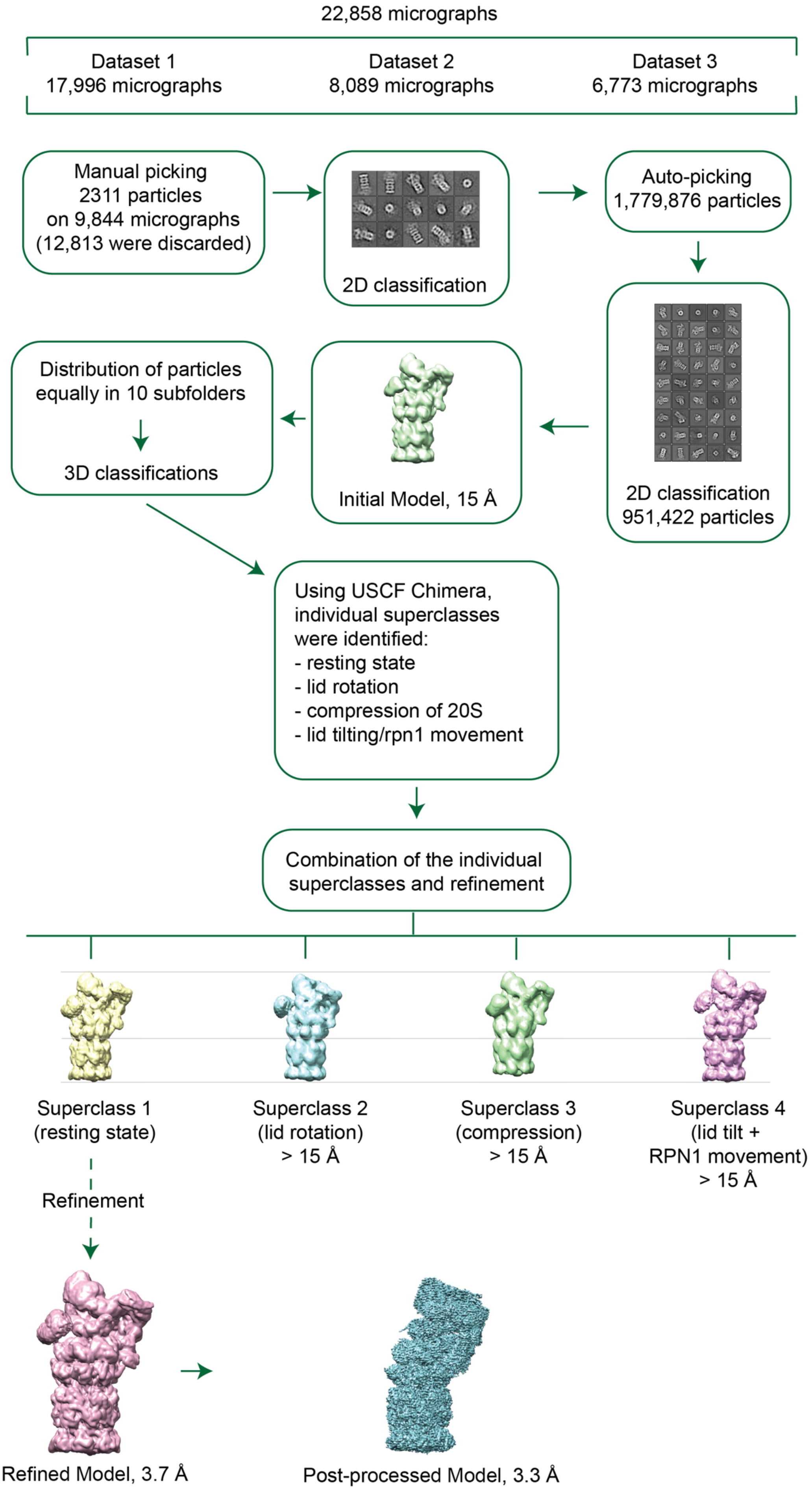

After collecting cryo-EM data, the micrographs were first checked for quality. After using the MQC (micrograph quality checker, CowSuite) to discard contaminated or bad micrographs, manual and auto-picking lead to a reasonable initial model. Due to 3D classification and the help of UCSF Chimera, it was possible to have a first look into the dynamics of the proteasome. Superclass 1 showed the resting state of the protein, Superclass 2 could be identified as the well-known lid rotation, Superclass 4 showed a series of lid tilting and RPN1 movements. Superclass 3 was a new conformation, that has not yet been described. Surprisingly, this was a motion of the 20S, rather than the RP. Together with a compression movement a partly gate opening of the non-occupied α-ring could be observed. The best 3D classes of each superclass were then combined and refined. While Superclass 1 led to a refined model with a resolution of 3.3 Å, the other superclasses reached a reasonable resolution to distinguish the different conformations, but not enough for further investigation.

Figure S5. Cryo-EM analysis.

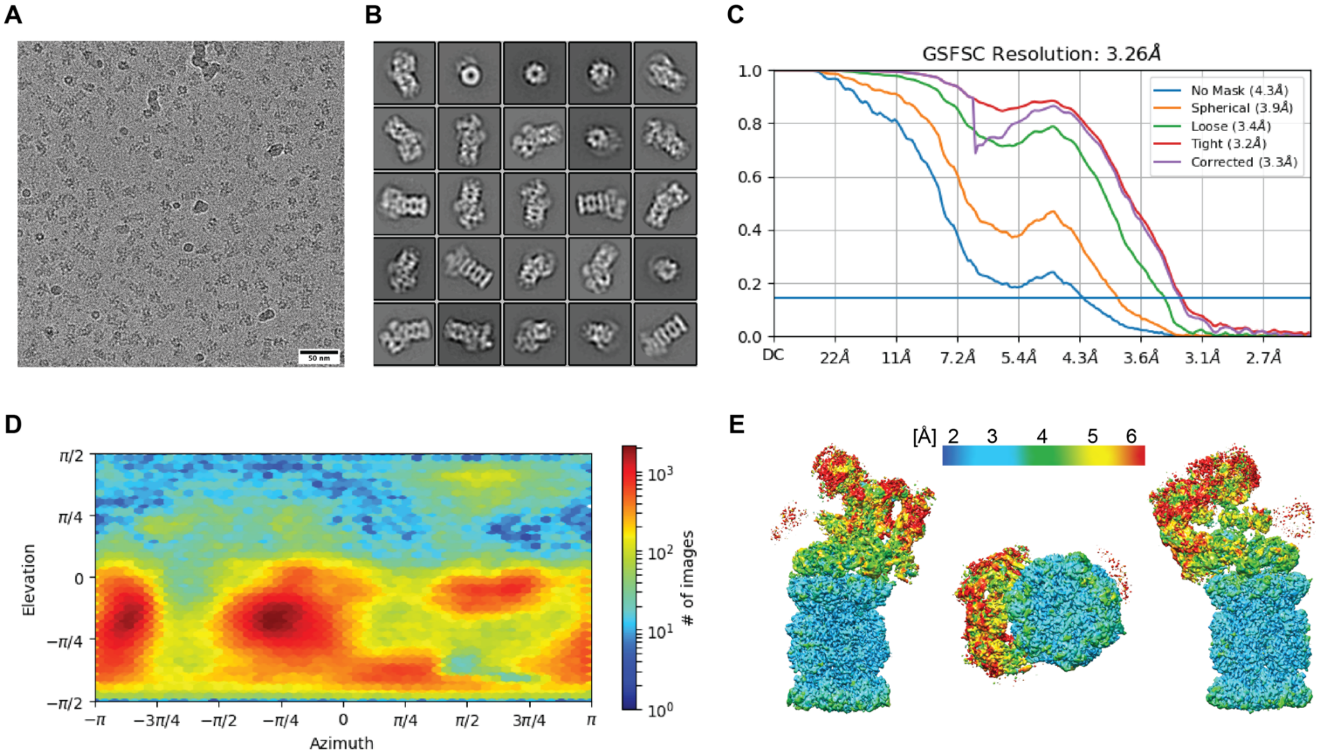

(A) A typical raw electron micrograph of the spinach 26S proteasomes embedded in vitreous ice. **(B)** Representative 2D class averages used for auto-picking. **(C)** Fourier shell correlation (FSC) curve. Average resolution of the reconstruction with C_1 symmetry, estimated to be 3.26 Å based on the gold-standard FSC criterion of 0.143. **(D)** Angle distribution of the refined map is shown. **(E)** In this local resolution map, each part of the density is colored according to the local resolution as specified in the color bar. The resolution ranges from 2 Å (blue) to 6 Å (red).

Figure S6. Pores of the spinach 26S proteasome.

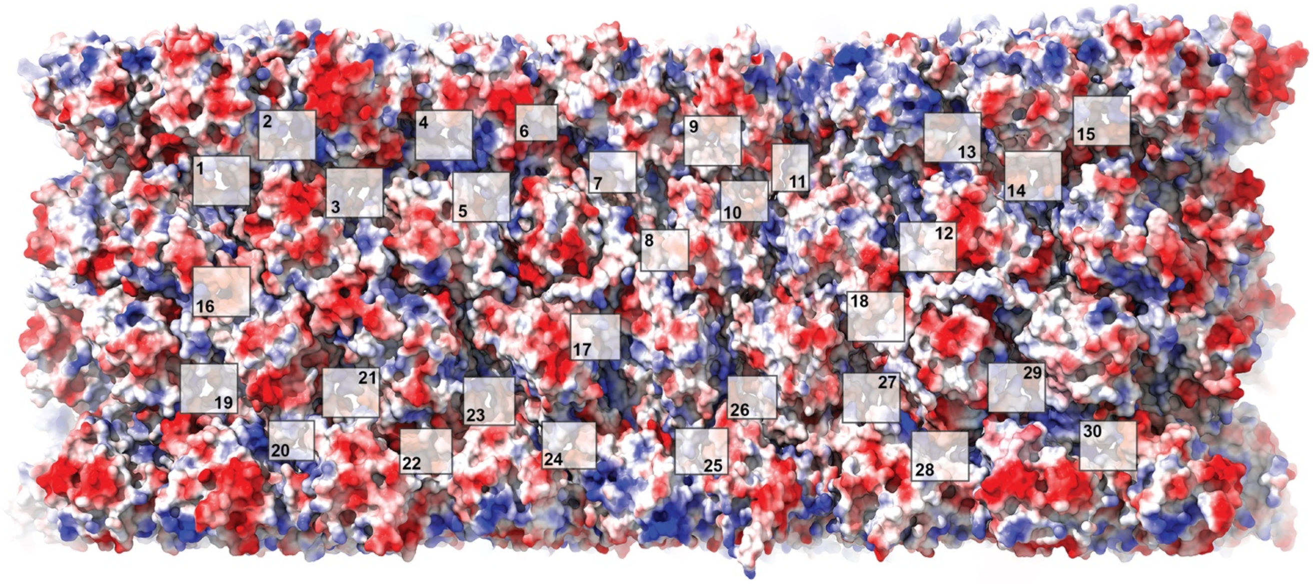

The panorama view shows the pores as an overview. The table contains the surrounding subunits, the longest and shortest axis, and a close-up snapshot of each pore. The numbering corresponds to the panorama view.

Pore Nr.	Surrounding subunits	Longest diameter [Å]	Shortest diameter [Å]	
1	$\alpha 7$ - $\beta 1$ - $\beta 7$	8.4	5.1	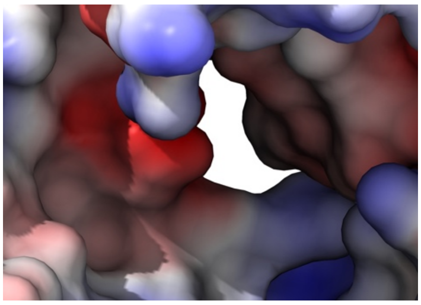
2	$\alpha 1$ - $\alpha 7$ - $\beta 1$	9.3	3.6	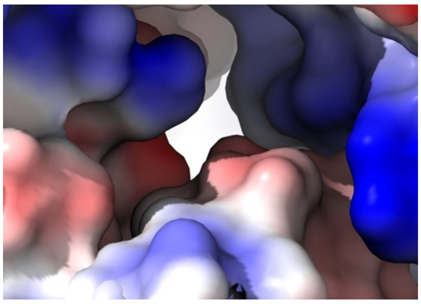

3	α1 - β1 - β2	16.1	8.4	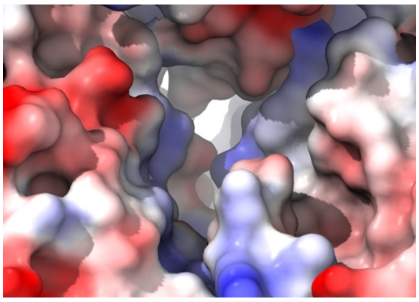
4	α1 - α2 - β2	16.9	7.7	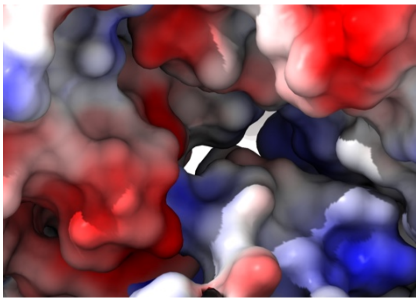
5	α2 - β2 - β3	13.3	3.3	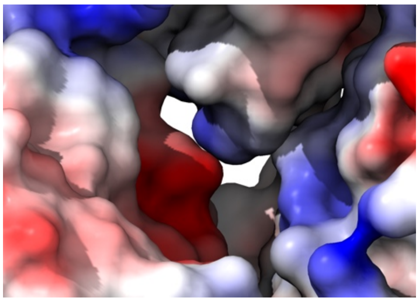
6	α2 - α3 - β3	11.8	6.7	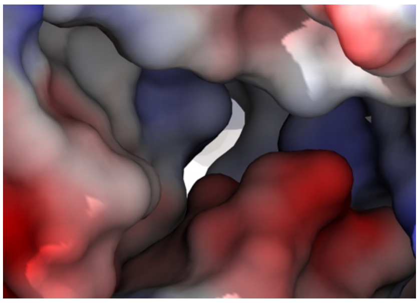
7	α3 - β3 - β4	8.2	5.7	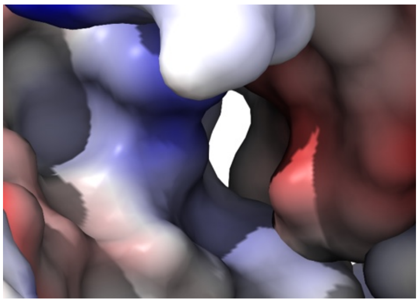

8	β3 - β4 - β5	8.9	8.2	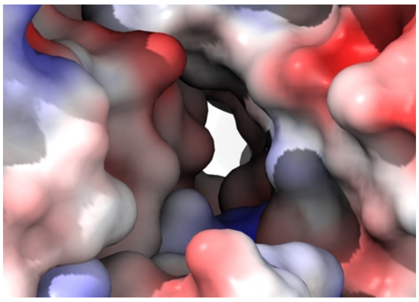
9	α3 - α4 - β4	11.1	5.1	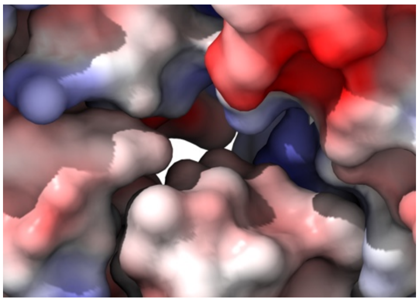
10	α4 - β4 - β5	14.0	3.4	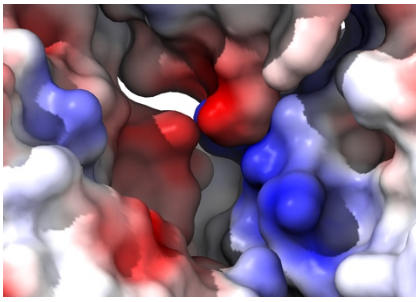
11	α4 - α5 - β5	13.4	5.4	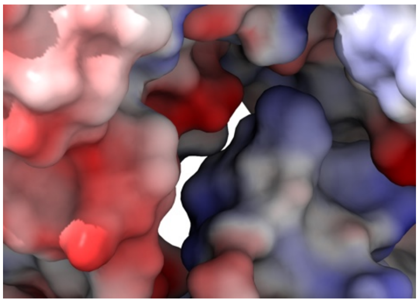
12	β5 - β6	10.0	6.5	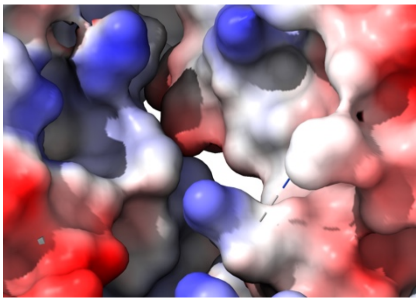

13	α6 - α5 - β6	8.3	7.7	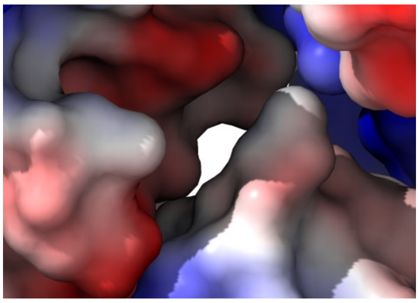
14	α6 - β6 - β7	8.9	5.9	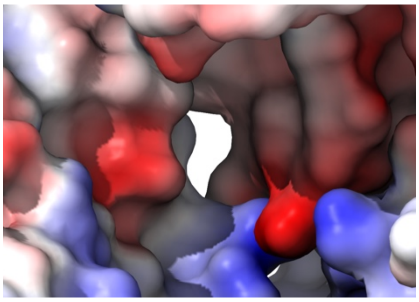
15	α6 - α7 - β7	9.8	9.2	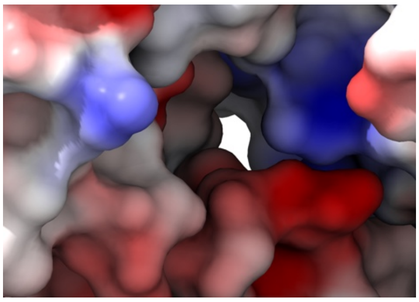
16	β1 - β7	4.2	4.1	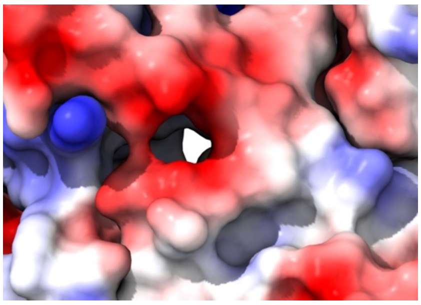
17	β5 - β6	9.6	6.0	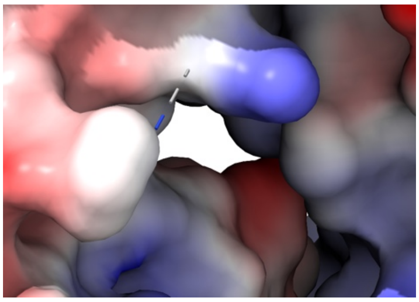

18	β3 - β4 - β5	6.4	6.1	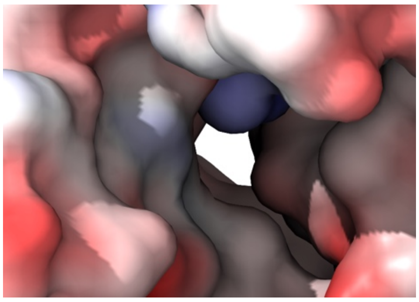
19	α1 - β1 - β2	6.5	5.4	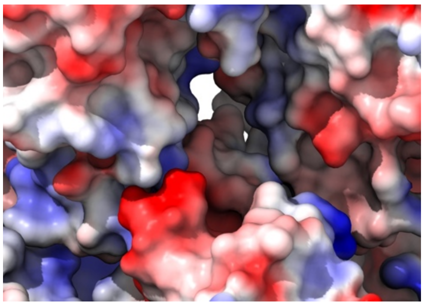
20	α1 - α7 - β1	8.6	7.7	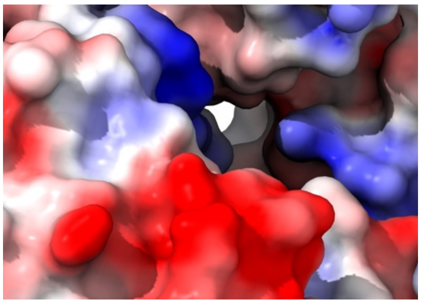
21	α7 - β1 - β7	11.7	14.7	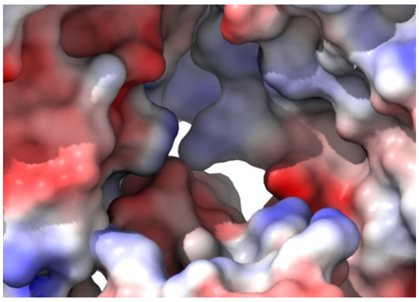
22	α6 - α7 - β7	11.7	11.1	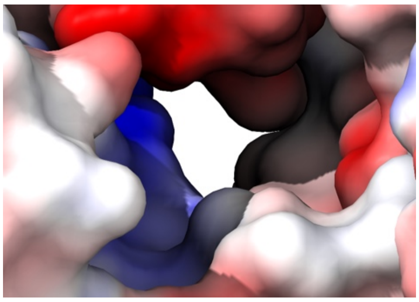

23	α6 - β6	8.4	5.6	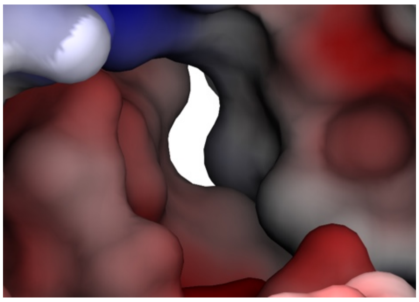
24	α5 - α6 - β6	6.9	5.0	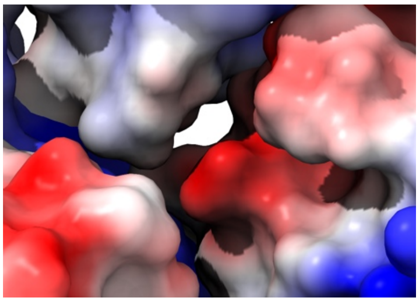
25	α4 - α5 - β5	16.9	4.8	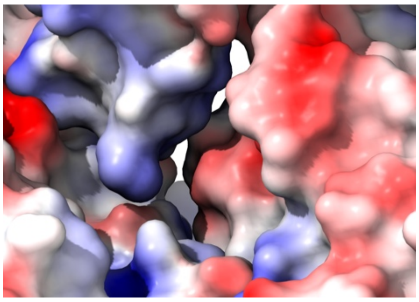
26	α4 - β4 - β5	14.0	6.1	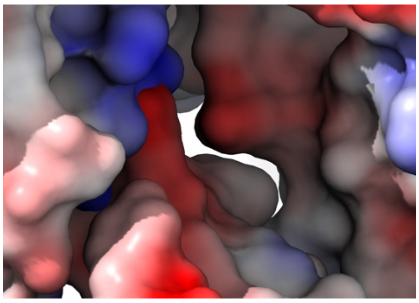
27	α3 - β3 - β4	8.4	7.3	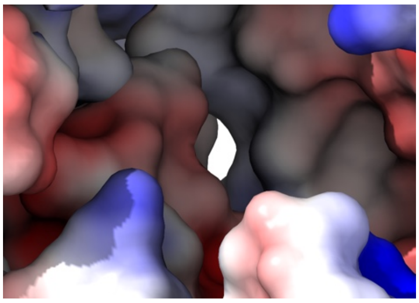

28	α2 - α3 - β3	10.9	10.8	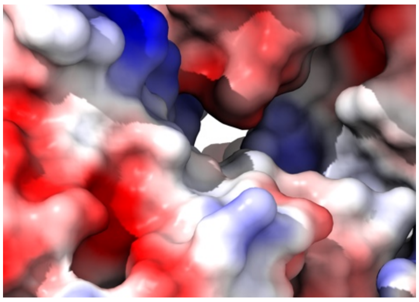
29	α2 - β2 - β3	17.5	4.5	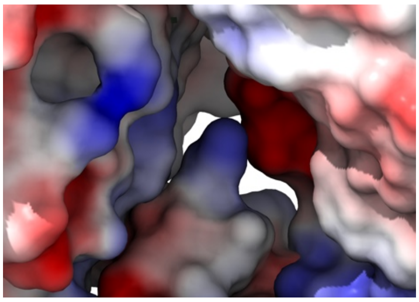
30	α1 - α2 - β2	10.4	4.1	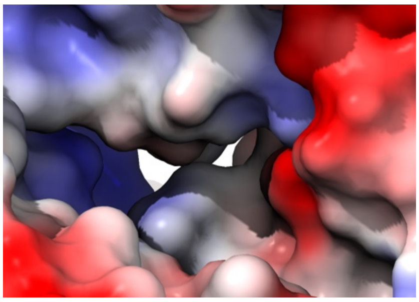

Figure S7. Sequence alignments of the spinach 26S proteasome isoforms.
The alignments show the different isoforms of the 26S proteasome subunits that were found in our preparation of *Spinacia oleracea*, including the calculated identity and similarity of their sequence.

<u>Isoforms of the CP:</u>

```
alpha3A MSRRYDSRTTIFSPEGRLYQVEYAMEAIGNAGSAIGILAKDGVVLIGEKKVTSKLLQTST 60
alpha3B MSRRYDSRTTIFSPEGRLYQVEYAMEAIGNAGSAIGILANDGVVLVGEKKVTSKLLQTST 60
        ***************************************:*****:**************

alpha3A STEKMYKIDDHVACAVAGIMSDANILINTARVQAQRYTFSYQEPMPVEQLVQSLCDTKQG 120
alpha3B SNEKMYKIDDHVACAVAGIMSDANILINTARVQAQRYTFSYQESMPVEQLVQSLCDTKQG 120
        *.***************************************** ****************

alpha3A YTQFGGLRPFGVSFLFAGWDKNYGFQLYMSDPSGNYGGWKATAIGANNQAAQSMLKQDYK 180
alpha3B YTQFGGLRPFGVSFLFAGWDKNYGFQLYMSDPSGNYGGWKAMAIGANNQAAQSMLKQDYK 180
        ***************************************** ******************

alpha3A DDVTREDAVKLALKVLSKTMDSTSLTSEKLELAEVYLLPSGKVKYQVHSPESLNRLLTES 240
alpha3B DDINREGAVQLALKVLGKTMDSTSLTSEKLELAEVYLLPSGKVKYQVHSPESLNELLAIA 240
        **:.**.**:******.*************************************.**: :

alpha3A GLTQPAAETS 250
alpha3B GLTQPAAEAS 250
        ********:*
```

Identity 94% - Similarity 95.6%

```
alpha3A MSRRYDSRTTIFSPEGRLYQVEYAMEAIGNAGSAIGILAKDGVVLIGEKKVTSKLLQTST 60
alpha3C MSRRYDSRTTIFSPEGRLYQVEYAMEAIGNAGSAIGILAKDGVVLIGEKKVTSKLLQTST 60
        ************************************************************

alpha3A STEKMYKIDDHVACAVAGIMSDANILINTARVQAQRYTFSYQEPMPVEQLVQSLCDTKQG 120
alpha3C STEKMYKIDDHVACAVAGIMSDANILINTARVQAQRYTFSYQEPMPVEQLVQSLCDTKQG 120
        ************************************************************

alpha3A YTQFGGLRPFGVSFLFAGWDKNYGFQLYMSDPSGNYGGWKATAIGANNQAAQSMLKQDYK 180
alpha3C YTQFGGLRPFGVSFLFAGWDKNYGFQLYMSDPSGNYGGWKATAIGANNQAAQSMLKQDYK 180
        ************************************************************

alpha3A DDVTREDAVKLALKVLSKTMDSTSLTSEKLELAEVYLLPSGKVKYQVHSPESLNRLLTES 240
alpha3C DDVTREDAVKLALKALSKTMDSTSLTSEKLELAEVYLLPSGKVKYQVHSPESLNRLLTES 240
        **************.*********************************************

alpha3A GLTQPAAETS 250
alpha3C GLTQPAAETS 250
        **********
```

Identity 99.6% - Similarity 99.6%

```
alpha3B MSRRYDSRTTIFSPEGRLYQVEYAMEAIGNAGSAIGILANDGVVLVGEKKVTSKLLQTST 60
alpha3C MSRRYDSRTTIFSPEGRLYQVEYAMEAIGNAGSAIGILAKDGVVLIGEKKVTSKLLQTST 60
        ***************************************:*****:**************

alpha3B SNEKMYKIDDHVACAVAGIMSDANILINTARVQAQRYTFSYQESMPVEQLVQSLCDTKQG 120
alpha3C STEKMYKIDDHVACAVAGIMSDANILINTARVQAQRYTFSYQEPMPVEQLVQSLCDTKQG 120
        *.***************************************** ****************

alpha3B YTQFGGLRPFGVSFLFAGWDKNYGFQLYMSDPSGNYGGWKAMAIGANNQAAQSMLKQDYK 180
alpha3C YTQFGGLRPFGVSFLFAGWDKNYGFQLYMSDPSGNYGGWKATAIGANNQAAQSMLKQDYK 180
        ***************************************** ******************

alpha3B DDINREGAVQLALKVLGKTMDSTSLTSEKLELAEVYLLPSGKVKYQVHSPESLNELLAIA 240
alpha3C DDVTREDAVKLALKALSKTMDSTSLTSEKLELAEVYLLPSGKVKYQVHSPESLNRLLTES 240
        **:.**.**:****.*.*************************************.**: :

alpha3B GLTQPAAEAS 250
alpha3C GLTQPAAETS 250
        ********:*
```

Identity 93.6% - Similarity 95.2%

```
alpha7A MSSIGTGYDLSVTTFSPDGRVFQIEYAAKAVDNSGTAVGIKCKDGIVLGVEKLIQSKMML 60
alpha7B MSSIGTGYDLSVTTFSPDGRVFQIEYASKAVDNSGTAVGIKCKDGIVLGVEKLIQSKMML 60
        ***************************:********************************

alpha7A PGSNRRIHSVHRHSGMAVAGLAADGRQIVARAKSEATNYESVYGEAVPVKELADRVASYV 120
alpha7B PGSNRRIHSVHRHSGMAVAGLAADGRQVVARAKSEATNYESVYGEAVPVKELADRVASYV 120
        ***************************:********************************

alpha7A HLCTLYWWLRPFGCGVILGGYDRDGPQLYMVEPSGISYRYFGAAIGKGKQAAKTEIEKLK 180
alpha7B HLCTLYWWLRPFGCGVILGGYDRDGPQLYMVEPSGISYRYFGAAIGKGKQAAKTEIEKLK 180
        ************************************************************

alpha7A LSEMTCREGIIEVAKIIYKVHDEAKDKAFELEMSWICDESKREHQKVPDNLLQEAKAAAT 240
alpha7B LSEMSCREGIIEVAKIIYKVHDEAKDKAFELEMSWICDESKREHQKVPDNLVEEAKAAAT 240
        ****:**********************************************::*******

alpha7A AALEEMDAD 249
alpha7B AALEEMDAD 249
        *********
```

Identity 98% - Similarity 100%

Isoforms of the AAA-ATPase:

```
RPT1A MAIEHEDDLKDEKNPRPLDEDDIALLKTYGLGPYSASIKKVEKEIKDMSKKVNDLIGIKE 60
RPT1B MGTEHED--DEDKKVTPLDEFDIEFLKTYNSSAYSTSIDNLEKEIREIVQRVADLRGIEE 58
      *. ****  .::*:  **** ** :****. . **:**.::****::: ::* ** **:*

RPT1A SDTGLAAPSQWDLVSDKQMMQEEQPLQVARCTKIINPNTEDAKYVINVKQIAKFVVGLGD 120
RPT1B SDTGLATPDKWDLKSDRQMWERS---------------------------LMRKMIVKLDG 92
      ******:*.:*** **:** :..                           : *::* *..

RPT1A KVSPTDIEEGMRVGVDRNKYQIQIPLPPKIDPSVTMMTVEEKPDVTYNDVGGCKEQIEKM 180
RPT1B RVSPTDIEEGMRVGVDRITWQIRMPLPPRHHRDASKSIIEEKPDITYNDIGGCKEQIQQI 152
      :**************** .:**::****: . ..:   :*****:****:*******:::

RPT1A REVVELPMLHPEKFVKLGIDPPKGVLCYGPPGTGKTLLARAVANRTDACFIRVIGSELVQ 240
RPT1B REIVELPMVAPEKFVRLGIEPPKGVLLYGPPGTGKTLVAKAVANRTDACFIRVNGTELVR 212
      **:*****: *****:***:****** **********:*:************* *:***:

RPT1A KYVGEGARMVRELFQMARSKKACIVFFDEVDAIGGARFDDGVGGDNEVQRTMLEIVNQLD 300
RPT1B KSPGQGAVMVRQLF------KGCIIFFDEVDAIGGARYFDGAGGDYEVQRTMLEIVNQLD 266
      *  *:** ***:**      *.**:************: **.*** **************

RPT1A GFDARGNIKVLMATNRPDTLDPALLRPGRLDRKVEFGLPDLEGRTQIFKIHTRTMNCERD 360
RPT1B GFDARGNVKVLMATNRPDTLDPALVRPGRMDRKVEFGLPDLEGRTQIFRIHTKNMSIKRG 326
      *******:****************:****:******************:***:.*. :*.

RPT1A IRFELLARLCPNSTGADIRSVCTEAGMYAIRARRKTVTEKDFLDAVNKVIKGYQKFSATP 420
RPT1B IRFELLARLCPNCTGADLRSVCNEAGMFAIRARRKIVTEKDFLDAVHKVISGYHKFSATP 386
      ************.****:****.****:******* **********:***.**:******

RPT1A KYMVYN 426
RPT1B EYMLYN 392
      :**:**
```

Identity 66.7% - Similarity 78.4% (RPT1B was not found in the mass spectrometry data)

```
RPT2A MGQNSSGLNRQGGPPGDRKDGEKKEKKYEPAAPPARVGRKQRKQKGSESAARIPTVTPLT 60
RPT2B MGQGPSGLNRPPNSGDKNQNSDKKDKKFEPAAPPARVGRKQRKQRGPDAAARVPVVTPHT 60
      ***. *****  .  ...::.:**:**:****************:* ::***:*.*** *

RPT2A KCKLRLLKLERIKDYLLMEEEFVANQERLKPQEEKTEEDRSKVDDIRGSPMSVGSLEELI 120
RPT2B KCKLRLLKLERVKDYLLMEEEFVANQERLKPQEEKTEEDRSKVDDIRGSPMSVGNLEELI 120
      ***********:******************************************.*****

RPT2A DENHAIVSSSVGPEYYVSIMSFVDKDQLEPGCSILMHNKVLSVVGLLQDEVDPMVSVMKV 180
RPT2B DENHAIVSSSVGPEYYVGIMSFVDKDQLEPGCSILMHNKVLSVVGILQDEVDPMVSVMKV 180
      *****************.***************************:**************

RPT2A EKAPLESYADIGGLDPQIQEIKEAVELPLTHPELYEDIGIKPPKGVILYGEPGTGKTLLA 240
RPT2B EKAPLESYADIGGLDPQIQEIKEAVELPLTHPELYEDIGIKPPKGVILYGEPGTGKTLLA 240
      ************************************************************

RPT2A KAVANSTSATFLRVVGSELIQKYLGDGPKLVRELFRVADDLSPSIVFIDEIDAVGTKRYD 300
RPT2B KAVANSTSATFLRVVGSELIQKYLGDGPKLVRELFRVADELSPSIVFIDEIDAVGTKRYD 300
      ***************************************:********************

RPT2A AHSGGEREIQRTMLELLNQLDGFDSRGDVKVILATNKIESLDPALLRPGRIDRKIEFPLP 360
RPT2B AHSGGEREIQRTMLELLNQLDGFDSRGDVKVILATNKIESLDPALLRPGRIDRKIEFPLP 360
      ************************************************************

RPT2A DIKTRRRIFTIHTSKMTLSDDVNLEEFVMTKDEFSGADIKAICTEAGLLALRERRMKVTH 420
RPT2B DIKTRRRIFTIHTSKMTLAEDVNLEEFVMTKDEFSGADIKAICTEAGLLALRERRMKVTH 420
      ******************::****************************************

RPT2A TDFKKAKEKVMFKKKEGVPEGLYM 444
RPT2B VDFKKAKEKVMYKKKEGVPEGLYM 444
      .**********:************
```

Identity 93.1% - Similarity 96.9%

```
RPT4A MATEEDVKRRAATSEYNKKLLQHRELESRSRKVKEELRSAKKDYTKTEDDLKSLQSVGQI 60
RPT4B MSTEEEVKRKAAVSEYNKKLLQHRELESRSRKVKEELRSAKKDYTKTEDDLKSLQSVGQI 60
      *:***:***:**.***********************************************

RPT4A IGEVLRPLDDERLIVKASSGPRYVVGCRSKVDKEKLTSGTRVVLDMTTLTIMRALPREVD 120
RPT4B IGEVLRPLDNERLIVKASSGPRYVVGCRSKVDKEKLTSGTRVVLDMTTLTIMRALPREVD 120
      ********:***************************************************

RPT4A PVVYNMLHEDPGNISYSAVGGLSDQIRELRESIELPLMNPELFIRVGIKPPKGVLLYGPP 180
RPT4B PVVYNMLHEDPGNISYSAVGGLSDQIRELRESIELPLMNPELFIRVGIKPPKGVLLYGPP 180
      ************************************************************

RPT4A GTGKTLLARAIASNIDANFLKVVSSAIIDKYIGESARLIREMFNYAREHQPCIIFMDEID 240
RPT4B GTGKTLLARAIASNIDANFLKVVSSAIIDKYIGESARLIREMFNYAREHQPCIIFMDEID 240
      ************************************************************

RPT4A AIGGRRFSEGTSADREIQRTLMELLNQLDGFDQLGKVKMIMATNRPDVLDPALLRPGRLD 300
RPT4B AIGGRRFSEGTSADREIQRTLMELLNQLDGFDQLGKVKMIMATNRPDVLDPALLRPGRLD 300
      ************************************************************

RPT4A RKIEIPLPNEQSRMEILKIHGAGIAKHGEIDYEAVVKLAEGFNGADLRNICTEAGMSAIR 360
RPT4B RKIEIPLPNEQSRMEILKIHAAGIAKHGEIDYEAVVKLAEGFNGADLRNICTEAGMSAIR 360
      *******************.****************************************

RPT4A AERDYVIHEDFMKAVRKLNEAKKLESSATYSADFGKD 397
RPT4B AERDYVIHEDFMKAVRKLNEAKKLESSATYSADFGKD 397
      *************************************
```

Identity 98.5% - Similarity 99.5%

```
RPT6A --------MATMEADQKTVKQGEGLRQYYLQHIHELQLRVRNKNHNLQRLEAQRNDLNSH 52
RPT6B MATALTERSSPATMGGTATTHGDGLRQYYLKRINELEHLHRQKTLDLSRLEAHRNELNSQ 60
               :    . .:..:*:*******::*:**:   *:*. :*.****:**:***:

RPT6A VRALKEELQLLQEPGSYVGEVVKVMGKSKVLVKVHPEGKYVVDIDKNIDITKLTPTTRVA 112
RPT6B VRMLKEELMLLQEPGSYVGEVVKVMGKSKVLVKVHPEGKYVVDVDKSIDITKLTPSTRVA 120
      ** ***** **********************************:**.********:****

RPT6A LRNDSYVLHLVLPSKVDPLVNLMKVEKVPDSTYDMIGGLDQQIKEIKEVIELPIKHPELF 172
RPT6B LRNDSYVLHLVLPSKVDPLVNLMKVEKVPDSTYDMIGGLDQQIKEIKEVIELPIKHPELF 180
      ************************************************************

RPT6A ESLGIAQPKGVLLYGPPGTGKTLLARAVAHHTDCTFIRVSGSELVQKYIGEGSRMVRELF 232
RPT6B ESLGIAQPKGVLLYGPPGTGKTLLARAVAHHTDCTFIRVSGSELVQKYIGEGSRMVRELF 240
      ************************************************************

RPT6A VMAREHAPSIIFMDEIDSIGSTRMESGSGNGDSEVQRTMLELLNQLDGFEASNKIKVLMA 292
RPT6B VMAREHAPSIIFMDEIDSIGSARMESGSGNGDSEVQRTMLELLNQLDGFEASKKIKVLMA 300
      *********************:******************************:*******

RPT6A TNRIDILDPALLRPGRIDRKIEFPNPTEESRFDILKIHSRRMNLMRGIDLKKIGDKMNGA 352
RPT6B TNRIDILDPALLRPGRIDRKIEFPNPNEDSRGDILKIHSRKMNLMRGIDLKKIAEKMNGA 360
      **************************.*:** ********:************.:*****

RPT6A SGAELKSVCTEAGMFALRERRIHVTQEDFEMAVAKVMKKDTDKNMSLRKLWK 404
RPT6B SGAELKAVCTEAGMFALRERRIHVTQEDFEMAVAKVMKKDNEKNMSLRKLWK 412
      ******:*********************************.:**********
```

Identity 87.7% - Similarity 91.1%

Isoforms of the RP:

```
RPN5A --------------------MDNGGNLEAQIDALLNVEKQMRLAGDVAGTRKAACDILDL 40
RPN5B FSRNPLISDDSFLSLQLAMADQKGGNLEAQIDALLNVGKQMRLFGDVDGTRKASCDILDL 60
                           ::************** ***** *** *****:******

RPN5A CFQSKAWKTLNDQIVVLSKRRGQLKQAVTAMVQQAMGYIDQTPDLDIRVELIKTLNSVSA 100
RPN5B CFQSKAWETLNDQIVALSKR---FEQAVTSMVQQAMGYIDQTPDLHTRVDLIKTLNRVSA 117
      *******:*******.****   ::****:***************. **:****** ***

RPN5A GKIYVELERARLIKILAKIKEQQGLIDEAAELMQEIAVETFGAMAKTEKIAFILEQVRLC 160
RPN5B GKIYVEVERARLIKILAKIKEQQGLIDEAAELMQEIAVETFGAMAKTEKIAFILEQVRLC 177
      ******:*****************************************************

RPN5A LDRKDYIRAQILSRKISPRVFDIDPSKEKKKPKEGDNIVEEAPADIPSLPELKRIYYELM 220
RPN5B LDRKDYIRAQILSRKISPRVFDIDPSKEKEKPKEGDNIVEEAPADIPSLTELKRIYYELM 237
      *****************************:******************* **********

RPN5A IRYYKHHNDYLEICRCYKSIYEISSVKEDPEQWTPILRKICWYLALAPHDPMQSSLLNST 280
RPN5B IRYYKHHNDYLEICRCYKAIYEISSVKEDPEQWTPILRKICWYLALAPHDPMQSSLMNST 297
      ******************:*************************************:***

RPN5A LEDKNLFEIPKFKSLLKQLVTMEVILWTVLWNEFESEFDNEKNLLGGPLGEKAGEDLKQR 340
RPN5B LEDKNLYEIPKFKSLLKQLVTIKVILWTDLWNEFEGEFDNEKNLLGGSLGEKAAEDLKQR 357
      ******:**************::***** ******.*********** *****.******

RPN5A VIEHNILVISKYYSRITLKRLSDLLCLSLQEAEKHLSDMVVSKALIAKIDRPMGIVCFQV 400
RPN5B VIEHNILVISKYNSKITLKRLSDLLCLSIQETEKHLSDMVVSKAIVAKIDRPMGIVCFQV 417
      ************ *:*************:**:************::**************

RPN5A VKDSNDILNSWSMNLEKLLDLVEKSCHQIHKETMVHKASLEV 442
RPN5B VKDSNDILNSWSINLEKLLGLVEKSCHQIHKETMIHKAALKV 459
      ************:******.**************:***:*:*
```

Identity 86.6% - Similarity 90.9%

```
RPN6A MATFLPATTDSLAQALEASSTADSILILQRLLADPSSSPDALRIKEQAITKLTDYLRQEN 60
RPN6B MATFLPATTDSLAQALEANSTADSILILQRLLADPSSSPDALRIKEQAITKLTDCLRQEN 60
      ******************.*********************************** *****

RPN6A KAEDLRILLTQLRSYFSLIPKAKTAKIVRVIIDTVAKIPNSTELQISLCKDMIQWTRDEK 120
RPN6B KAEDLRILLTQLRSYFSLIPKAKTAKIVRGIIDTVAKIPNSTELQISLCKDMVQWTRDEK 120
      ***************************** **********************:*******

RPN6A RTFLRQRVEARLAALLMETKEFPEALSLLSGLIKEVRRLDDKLLLVDIELLESKLHFSLR 180
RPN6B RTFLRQRVEARLAALLMETKEYPEALSLLSGLIKEVRRLDDKLLLVDIELLESKLHFSLR 180
      *********************:**************************************

RPN6A NLPKAKAALTAARTAANAIYVPPAQQGTIDLQSGILHAEEKDYKTGYSYFYEAFEAFNAL 240
RPN6B NLPKAKAALTAARTAANAIYVPPAQQGTIDLQSGILHAEEKDYKTGYSYFYEAFEAFNAL 240
      ************************************************************

RPN6A EDPRAVYSLKYMLLCKVMVNQADDVAGIISSKAGLQYLGPDLDAMKAIADAYSKRSLKLF 300
RPN6B DDPRAVYSLKYMLLCKVMVNQADDVAGIISSKAGLKYLGPELDAMKAIADAYSKRSLKLF 300
      :**********************************:****:*******************

RPN6A EASLVNFKAQLQEDPIIHRHLSSLYDTLLEQNLCRLIEPFSKVEISHIAELIELPVDHVE 360
RPN6B EASLVNFKAQLQEDPIIHRHLSSLYDTLLEQNLCRLIEPFSRVEISHIAELIELPVDHVE 360
      *****************************************:******************

RPN6A RKLSQMILDKKFAGTLDQGAGCLIIFDDHKTEDIYEATLDTISNVAKVVDSLFVRSAKIM 420
RPN6B RKLSQMILDKKFAGTLDQGAGCLIIFDDPKTEAIYEATLDTISNVAKVVDSLFVRSAKIM 420
      **************************** *** ***************************

RPN6A A 421
RPN6B A 421
      *
```

Identity 97.4% - Similarity 99%

```
RPN8A MDVIKTQQIFSSKSIEKVVVHPLVLLSIVDHYNRVARDTKKRVIGVLLGSTFKGTVDVTN 60
RPN8B MDVVKIQQISSSKAIEKVVVHPLVLLSIVDHYNRVARDTKKRVIGVLLGSTFKGTVDVTN 60
      ***:* *** ***:**********************************************

RPN8A SYAVPFEEDDKDSSIWFLDHNYHESMFSMFRRINAKEHVVGWYSTGPKLRENDLDVHRLF 120
RPN8B SYAVPFEEDDKDPSIWFLDHNYHESMFSMFRRINAKEHVVGWYSTGPKLRENDLDVHRLF 120
      ************ ***********************************************

RPN8A SDYVPNPVLVIIDVQPEELGIPTKAYYAVEEVKENATQKSQKVFVHVPSEIAAHEVEEIG 180
RPN8B SDYVPNPVMVIIDVQPEELGIPTKAYYAVEEVKENATQKSQKVFAHVPSEIAAHEVEEIG 180
      ********:***********************************.***************

RPN8A VEHLLRDVKDTTISTLATEVTGKLGALKGLDARLREIRSYLELVIQEKLPLNHEILYHLQ 240
RPN8B VEHLLRDVKDTTISTLATEVTGKLAALKGLDARLREIRGYLELVIEGKLPLNHEILYHLQ 240
      ************************.*************.******: *************

RPN8A DVFNLLPNLSVLELVKAFAVKTNDMMLVIYLSSLIRSVIALHNLINNKMLNKEHEKAEDS 300
RPN8B DVFNLLPNLSVLELVKAFAVKTNDMMLVIYLSSLIRSVIALHNLINNKMLNKEHEKSEDS 300
      ********************************************************:***

RPN8A KSLAITSVAGS 311
RPN8B KSIPVVAAAGG 311
      **: :.:.**.
```

Identity 93.9% - Similarity 96.5% (RPN8B was not found in the mass spectrometry data)

```
RPN9A MSALQYLDTLRSAHPELGEWYNTLADLYQKKLWHQLTLELEKFVALAVFQAGDALIQLYH 60
RPN9B MSAIEYLEAVNSSNPELGDWYSSFAHLYTKELWHQLTVELEKFIALPVFQTGDALIPFYH 60
      ***::**:::.*::****:**.::*.** *:******:*****:** ***:***** :**

RPN9A NFITDFETKINLLKLAHFAVIVSRQYAEKEAAIGYLEGVTEKLHATKENRIEEPVLYIKM 120
RPN9B NFITDFETKINLLKLAHFTVIVSRQYAEKEAAIGYLEGVIEKLHATKENRIEEPVLYVKM 120
      ******************:******************** *****************:**

RPN9A QIALFKLEQGEQKECKKLLDNGKTTLDSMTDIDPSVYATFYWVSSQYHKARQEFAEFYKN 180
RPN9B QIALLKLEQGEHKACKKLLEDGKTTLDSMTDIDPSVYASFYWVSSQYHKARQEFAEFYKS 180
      ****:******:* *****::*****************:********************.

RPN9A ALLYLAYTSVESLSESFKLDLAFDLSLSALLGENIYNFGELLAHPILKSLLGTKVEWLYY 240
RPN9B ALLYLAYTSVESLSESFKLDLAFDLSLSALLGENIYNFGELLAHPILKSLLGTKAEWLYY 240
      ******************************************************.*****

RPN9A ILQAFNTGDLIRYQELCNVHKDALNAQPALVANERKLLEKINILCLMEIIFNRPAEDRTI 300
RPN9B ILQAFNSGNLIRYQELCNVHKDALNAQPALVANERKLLEKINILCLMEIIFNRPAEDRTI 300
      ******:*:***************************************************

RPN9A PLKVIAERTRLSIEDVEYLLMKSLSVHLIEGIIDQMEGTVHVSWVQPRVLGIPQITSLRD 360
RPN9B PLKVIADRTRLSIEDVEYLLMKSLSVHLIEGIIDQVEGTVHVSWVQPRVLGIPQITALRA 360
      ******:****************************:********************:**

RPN9A RLDSWLGKVNTALLSVEAETPDLVAS 386
RPN9B RLDNWLGKVHTTLLSVEAETPDLIAS 386
      ***.*****:*:***********:**
```

Identity 89.1% - Similarity 95.6%

```
RPN11A MDRLTRMMAGAGGALGHPPPDSPTLDTSEQVYISSLALLKMLKHGRAGVPMEVMGLMLGE 60
RPN11B MDRLTRMFAGAGGALGHPPPDSPTLDTSEQVYISSLALLKMLKHGRAGVPMEVMGLMLGE 60
       *******:****************************************************

RPN11A FVDEYTVKVVDVFAMPQSGTGVSVEAVDPVFQTNMLDMLKQIGRPEMVVGWYHSHPGFGC 120
RPN11B FVDEYTVRVVDVFAMPQSGTGVSVEAVDHVFQTNMLDMLKQTGRPEMVVGWYHSHPGFGC 120
       *******:********************* *********** ******************

RPN11A WLSGVDINTQQSFEALNQRAVAVVVDPIQSVKGKVVMDAFRLINPQTMMLGQEPRQTTSN 180
RPN11B WLSGVDINTQQSFEALNQRAVAVVVDPIQSVKGKVVIDAFRLINPQTMMLGQEPRQTTSN 180
       ************************************:***********************

RPN11A LGHLNKPSIQALIHGLNRHYYSIAINYRKNELEEKMLLNLHKKKWNDGLTLKKFDVHSKT 240
RPN11B LGHLNKPSIQALIHGLNRHYYSIAINYRKNELEEKMLLNLHKKKWTNGLTLKRFDDHSKT 240
       *********************************************.:*****:** ****

RPN11A NEQTVQEMLGLAIKYNKAVQEEDELTPEKLVIAKVGRQDAKKHLEEHVSNLMSSNIIQTL 300
RPN11B NEQTVQEMLNLAIKYNKAVQEEDELTPEKLAIANVGRQDAKKHLEEHVSNLMSSNIVQTL 300
       *********.********************.**:**********************:***

RPN11A GTMLDTVIF 309
RPN11B GTMLDTVVF 309
       *******:*
```

Identity 95.5% - Similarity 97.4%

Table S1. 26S proteasome subunits nomenclature.

List of all subunits of the spinach 26S proteasome identified by mass spectrometry. The different isoforms are marked with (A) – representing the most abundant isoform – (B) or (C). The isoforms with an asterisk (*) couldn't be found in the mass spectrometry data.

Unified Nomenclature	Plant Gene Name	Spinach Gene Name (Uniprot)	Known Human Gene Name (Uniprot)	Known Yeast Gene Name (Uniprot)
**20S proteasome**				
α1	PAA	SOVF_093600	PSMA6 / PROS27	SCL1 / PRC2 / PRS2 / YC7
α2	PAB	SOVF_201960	PSMA2 / HC3 / PSC3	PRE8 / PRS4 / YC7
α3	PAC (A)<br>PAC (B)<br>PAC (C)	SOVF_067770<br>SOVF_188980<br>PAC1	PSMA4 / HC9 / PSC9	PRE9 / PRS5 / Y13
α4	PAD	SOVF_013850	PSMA7 / HSPC	PRE6
α5	PAE	SOVF_076310	PSMA5	PUP2 / DOA5
α6	PAF	SOVF_075310	PSMA1 / HC2 / NU / PROS30 / PSC2	PRE5
α7	PAG (A)<br>PAG (B)	PAG1<br>SOVF_007100	PSMA3 / HC8 / PSC8	PRE10 / PRC1 / PRS1 / YC1
β1	PBA	SOVF_007620	PSMB6 / LYMPY / Y	PRE3
β2	PBB	SOVF_003170	PSMB7 / Z	PUP1
β3	PBC	SOVF_015190	PSMB3	PUP3
β4	PBD	SOVF_145550	PSMB2	PRE1
β5	PBE	SOVF_078180	PSMB5 / LMPX / MB1 / X	PRE2 / DOA3 / PRG1
β6	PBF	SOVF_035230	PSMB1 / PSC5	PRE7 / PRS3 / PTS1
β7	PBG	SOVF_076470	PSMB4 / PROS26	PRE4
**19S regulator ATPase subunits**				
RPT1	RPT1 (A)<br>RPT1 (B)*	RPT1<br>SOVF_004100	PSMC2 / MSS1	YTA3 / CIM5
RPT2	RPT2 (A)<br>RPT2 (B)	SOVF_167680<br>SOVF_147780 / _210740	PSMC1	YTA5/mts2 / YHS4
RPT3	RPT3	SOVF_151020	PSMC4 / MIP224 / TBP7	YNT1 / YTA2
RPT4	RPT4 (A)<br>RPT4 (B)	SOVF_027030<br>SOVF_027040	PSMC6 / SUG2	SUG2 / PCS1 / CRL13
RPT5	RPT5	SOVF_156090	PSMC3 / TBP1	YTA1
RPT6	RPT6 (A)<br>RPT6 (B)	SOVF_183190<br>SOVF_175230	PSMC5 / SUG1	SUG1
**19S regulator non-ATPase subunits**				
RPN1	RPN1	SOVF_094190	PSMD2	HRD2 / NAS1/mts4
RPN2	RPN2	SOVF_045290	PSMD1	SEN3
RPN3	RPN3	SOVF_099120	PSMD3	SUN2
RPN5	RPN5 (A)<br>RPN5 (B)	SOVF_139310<br>SOVF_160100	PSMD12	NAS5
RPN6	RPN6 (A)<br>RPN6 (B)	SOVF_197890<br>SOVF_054100	PSMD11	NAS4
RPN7	RPN7	SOVF_135550	PSMD6	RPN7

RPN8	RPN8 (A)<br>RPN8 (B)*	SOVF_020490	PSMD7	NAS3
RPN9	RPN9 (A)<br>RPN9 (B)	SOVF_035360<br>SOVF_035380	PSMD13	NAS7/mts1
RPN10	RPN10	SOVF_154600	PSMD4	SUN1 / MCB1/pus1
RPN11	RPN11 (A)<br>RPN11 (B)	SOVF_184120<br>SOVF_184110	PSMD14	MPR1/pad1 / mts5
RPN12	RPN12 (A)	SOVF_037910	PSMD8	NON1/mts3
RPN13	RPN13	SOVF_136340	PSMD16 / ADRM1	DAQ1
RPN15	DSS1	SOVF_131370	PSMD15 / SEM1	SEM1

Table S2. Software and Algorithms.

	Source	
CHAINSAW	Stein, 2008	
ChimeraX	Goddard et al., 2018	https://www.cgl.ucsf.edu/chimerax/
Coot	Emsley and Cowtan, 2004	https://www2.mrc-lmb.cam.ac.uk/personal/pemsley/coot
Cow		http://www.cow-em.de
Gautomatch		https://www.mrc-lmb.cam.ac.uk/kzhang
MolProbity	Chen et al., 2010	http:/molprobity.biochem.duke.edu
MotionCor2		https://emcore.ucsf.edu/ucsf-software
Phenix	Adams et al., 2010	https://www.phenix-online.org
Relion	Zivanov et al., 2019	http://www2.mrc-lmb.cam.ac.uk/relion
ResMap	Kucukelbir et al., 2014	http://resmap.sourceforge.net
SerialEM	Mastronarde, 2005	https://bio3d.colorado.edu/SerialEM/
UCSF-Chimera	Pettersen et al., 2004	https://www.cgl.ucsf.edu/chimera

Table S3. Electron cryo microscopy data collection, refinement, and validation statistics.

	Data 1 / 2 / 3	
Data collection		
EM equipment	FEI Titan Krios	
Magnification	81,000	
Voltage (kV)	300	
Detector	Falcon 3 EC	
Pixel size (Å)	1.058	
Electron dose / Electron exposure (e^-/Å^2)	80 / 50 / 80	
Defocus range (μm)	2 - 4.5	
Symmetry imposed	C1	
Initial particle images (no.)	7996 / 8089 / 6773	
Final particle images (no.)	4718 / 4602 / 3987	
Map resolution (Å)	3.3	
FSC threshold	0.143	
Map resolution range (Å)	2.8 - 7	
Reconstruction		
Software	Relion, cryoSPARC	
Number of used particles	951,422	
Symmetry	C1	
Final resolution (Å)	3.3	
Map-sharpening *B* factor (Å^2)	109.9	
Model building		
Software	Chainsaw, Coot, Phenix	
Refinement		
Software	Phenix	
Average FSC	1.9	
	20S	19S
Model composition		
Protein residues	6256	6993
R.m.s. deviations		
Bond lengths (Å)	0.009	0.006
Bond angles (°)	1.190	1.188
Validation		
MolProbity score	1.95	1.77

Clash score	10.31	7.64
Poor rotamers (%)	0.70	0.00
Ramachandran plot		
Favored (%)	93.81	94.94
Allowed (%)	6.06	4.83
Outliers (%)	0.13	0.23

Movie S1. Movies of all mentioned states.

(A) Different modes from all used tools (Relion, CowSuite, cryoSPARC, PCA, cryoDRGN). **(B)** 2D classes of the bovine 26S proteasome combined in a video to show the compression movement.
