# Supplementary material for: Cryo-EM structure of the plant 26S proteasome
Source: Plant Commun. 2022 Mar 11;3(3):100310. doi: 10.1016/j.xplc.2022.100310 (PMC9251434; doi:10.1016/j.xplc.2022.100310)
Supplement: Document S2. Article plus supplemental information [file mmc4.pdf]

Plant Communications
Research Article

CellPress
Partner Journal

Cryo-EM structure of the plant 26S proteasome

Susanne Kandolf[1], Irina Grishkovskaya[1], Katarina Belačić[1,5], Derek L. Bolhuis[3], Sascha Amann[1,5], Brent Foster[2], Richard Imre[1], Karl Mechtler[1], Alexander Schleiffer[1], Hemant D. Tagare[2], Ellen D. Zhong[4], Anton Meinhart[1], Nicholas G. Brown[3] and David Haselbach[1,6,7,*]

[1]Research Institute of Molecular Pathology (IMP), Vienna BioCenter (VBC), Campus-Vienna-BioCenter 1, 1030 Vienna, Austria

[2]Department of Radiology and Biomedical Imaging, Yale University, New Haven, CT 06510, USA

[3]Department of Pharmacology and Lineberger Comprehensive Cancer Center, University of North Carolina School of Medicine, Chapel Hill, NC 27599, USA

[4]Computer Science and Artificial Intelligence Laboratory, Massachusetts Institute of Technology, Cambridge, MA 02139, USA

[5]Vienna BioCenter PhD Program, Doctoral School of the University at Vienna and Medical University of Vienna, Vienna BioCenter (VBC), Vienna, Austria

[6]Institute of Physical Chemistry, University of Freiburg, Albertstraße 21, Freiburg 79104, Germany

[7]CIBSS Centre for Integrative Biological Signalling Studies, University of Freiburg, Freiburg, Germany

***Correspondence: David Haselbach (david.haselbach@imp.ac.at)**

https://doi.org/10.1016/j.xplc.2022.100310

ABSTRACT

Targeted proteolysis is a hallmark of life. It is especially important in long-lived cells that can be found in higher eukaryotes, like plants. This task is mainly fulfilled by the ubiquitin–proteasome system. Thus, proteolysis by the 26S proteasome is vital to development, immunity, and cell division. Although the yeast and animal proteasomes are well characterized, there is only limited information on the plant proteasome. We determined the first plant 26S proteasome structure from *Spinacia oleracea* by single-particle electron cryogenic microscopy at an overall resolution of 3.3 Å. We found an almost identical overall architecture of the spinach proteasome compared with the known structures from mammals and yeast. Nevertheless, we noticed a structural difference in the proteolytic active β1 subunit. Furthermore, we uncovered an unseen compression state by characterizing the proteasome's conformational landscape. We suspect that this new conformation of the 20S core protease, in correlation with a partial opening of the unoccupied gate, may contribute to peptide release after proteolysis. Our data provide a structural basis for the plant proteasome, which is crucial for further studies.

Key words: 26S proteasome, spinach, UPS, cryo-EM, conformational landscape

Kandolf S., Grishkovskaya I., Belačić K., Bolhuis D.L., Amann S., Foster B., Imre R., Mechtler K., Schleiffer A., Tagare H.D., Zhong E.D., Meinhart A., Brown N.G., and Haselbach D. (2022). Cryo-EM structure of the plant 26S proteasome. Plant Comm. **3**, 100310.

INTRODUCTION

The ubiquitin–proteasome system (UPS) is the most critical pathway for non-lysosomal protein degradation in all eukaryotes. With the help of a three-step conjugation cascade involving E1, E2, and E3 enzymes, protein substrates are labeled with a ubiquitin chain, which serves as a reusable recognition signal for selective protein turnover by the 26S proteasome (Ozaki et al., 1992; Vierstra, 1993). Most components of this system are well conserved in all branches of eukaryotes (Fujinami et al., 1994), with ubiquitin itself varying by only two or three amino acids from plants to humans. Interestingly, ~5% of the *Arabidopsis* genome encodes components of this pathway, more than 1300 of which are E3s (Vierstra, 2003), whereas there are "only" about 600 E3 genes known in humans (Li et al., 2008). The increased complexity of plants may stem from their sessile nature, which needs to cope with a considerable number of environmental stresses, such as temperature, weather, radiation, and chemical substances (Xu and Xue, 2019). In addition, the long plant cell cycle strongly requires targeted protein degradation, whereas fast-growing organisms like yeast can downregulate their protein levels via dilution through cell division.

Apart from cell homeostasis, the UPS is also involved in several systemic processes, especially plant immunity. Plants rely on a highly developed innate immune system to recognize pathogens and defend against pathogenic attacks. Therefore, the organism

Published by the Plant Communications Shanghai Editorial Office in association with Cell Press, an imprint of Elsevier Inc., on behalf of CSPB and CEMPS, CAS.

This is an open access article under the CC BY license (http://creativecommons.org/licenses/by/4.0/).

must be capable of mounting a strong and effective defense response while avoiding autoimmunity. The UPS is critical for the regulation of these processes through its involvement in oxidative bursts, hormone signaling, gene induction, and apoptosis (Trujillo and Shirasu, 2010).

Arguably, the most important component of the UPS is the 26S proteasome, which makes the final decision on protein fate and catalyzes protein degradation. It is a 1.7 MDa protein complex that acts as a multicatalytic ATP-dependent protease. The 20S core particle (CP) is given the proteolytic function and consists of four stacked heptameric rings. Thus, it has a barrel-like shape with a central cavity. The two inner rings are made up of seven different β subunits (termed PBA–PBG; see also Supplemental Table 1) (Fu et al., 1999) with three different protease active sites (PBA/β1, PBB/β2, PBE/β5) hidden within the inner chamber. The two outer rings are each composed of seven diverse α subunits (PAA–PAG) and control substrate entry via a gate. The gate opening is most commonly regulated by the 19S regulatory particle (RP) (Köhler et al., 2001), which can be bound to either one (26S proteasome) or both ends (30S proteasome) of the CP complex (Lander et al., 2012). It provides functionality for recognizing ubiquitinated proteins, unfolding and deubiquitination of the substrate, and threading of the unfolded polypeptides into the center of the CP for degradation. The RP can be further subdivided into a hexameric ring of RP AAA-ATPase subunits (AAA stands for ATPase associated with various cellular activities) (RPT1–6) and 13 non-ATPase subunits (RPN1–3, RPN5–13, and RPN15) (Finley et al., 1998). RPN1, RPN10, and RPN13 recognize polyubiquitin chains (Shi et al., 2016). RPN11 has a deubiquitination activity that can remove ubiquitin moieties bound to target proteins during their breakdown. Unfolding and threading of the substrate are catalyzed by the AAA-ATPase (Bard et al., 2018).

To fulfill all these functions, the RP samples a complex conformational landscape. Two conformations were initially identified, a substrate-free (S_A; resting state) and a substrate-processing (S_C) state, including a lid rotation of 30° relative to the base (Bard et al., 2018). Other intermediate states (S_B and S_D) have been identified that differ mainly in the degree of lid rotation (Dong et al., 2019). With progress in cryoelectron microscopy (cryo-EM) technology, further subclassifications of proteasome states have been identified that provide the work cycle of the ATPase (Raab et al., 2009; Eisele et al., 2018; Ding et al., 2019).

RESULTS

Although we have acquired substantial knowledge of the yeast and human proteasomes (Bard et al., 2018), we have only a limited understanding of the plant enzyme. Given the known differences in proteasome regulation between yeast and human, we also expected to find diversity in the proteasome of plants. We thus set out to investigate the structure of the plant proteasome. We adapted an affinity purification protocol for the human proteasome (Besche and Goldberg, 2012) that uses the ubiquitin-like (Ubl) domain of Rad23b as a bait to obtain comparable results. In the plant system, the Ubl domain of Rad23a led to better binding and similar yields. Our preparations contained all 33 integral subunits (Figure 1A) and at least two proteasomal interacting proteins (ECM29 and PSMD5) (Table 1; see also the complete mass spectrometry data).

In the next step, we assessed the activity of spinach 26S proteasomes alongside the activity of human 26S proteasomes using polyubiquitinated human securin as a model substrate. Both spinach and human proteases efficiently deubiquitinated and degraded the model substrate, despite sequence differences in ubiquitin and securin (Supplemental Figure 1). Estimating from the band intensity, we obtained an overall turnover of ub_n-securin* of about 1 min at 25°C, which is in line with published results (Peth et al., 2013; Bard et al., 2019).

After these quality-evaluation steps, we subjected our preparation to single-particle cryo-EM for structural investigation. We determined a 3.3 Å resolution map that enabled us to model 80% of the fragmented density (Figure 1B and 1C). Comparing their overall architectures, we found high similarity to the mammalian and budding yeast proteasomes (Unverdorben et al., 2014). By contrast, we found a significant difference in the functionally relevant β1 subunit (Figure 1E and 1F). Unlike previously solved proteasome structures, the C terminus of the β1 subunit has a 20 amino acid long tail that forms a new contact to the surface of β7. A similar C-terminal extension of β1 evolved independently in diverse taxonomic divisions, such as Euglenozoa, Nematoda, Chlorophyta, and Streptophyta. In higher plants, we even found a highly conserved tail (Figure 1), which may indicate a motif for an additional binding partner or act as a signal for modification.

Structure analysis

Despite considerable sequence differences in a few functionally important subunits, we cannot detect further significant changes in the folds and conformations of the RP. Interestingly, the enzyme seems to resemble the human proteasome more than the yeast complex. For instance, RPN13 appears to be fully flexible and invisible in our structure while being present in our sample, similar to human preparations. The α-ring and the AAA-ATPase are well conserved, as shown in Supplemental Figure 2. Specifically, the ATPase is almost identical except for one significant difference in the C-terminal region of the RPT subunits, which contain the CP-interacting hydrophobic-tyrosine-X (HbYX) motifs that control the gate. Although all proteasomes in eukaryotes have clear HbYX motifs on the RPT2, RPT3, and RPT5 subunits, an additional HbYX motif is conserved in plants. In higher plants, the three C-terminal residues of RPT1 are consistent with an HbYX motif (VYN in spinach), whereas in other organisms the degree of hydrophobicity in the third-to-last amino acid varies. In humans, this position is occupied by threonine, which is at least partially hydrophobic; in yeast, this position is taken by the hydrophilic glutamine (Supplemental Figure 3). This may indicate different activation mechanisms for the proteasomes of diverse organisms, as the interaction with this motif is important for 20S gate regulation (Opoku-Nsiah et al., 2021). Our refined map shows clear density inside the α1–α2 and α5–α6 pockets, suggesting the insertion of the C-termini of RPT3 and RPT5 (Figure 2). It has been observed that, while in the resting state, the HbYX tails of RPT3 and RPT5 are inserted into their

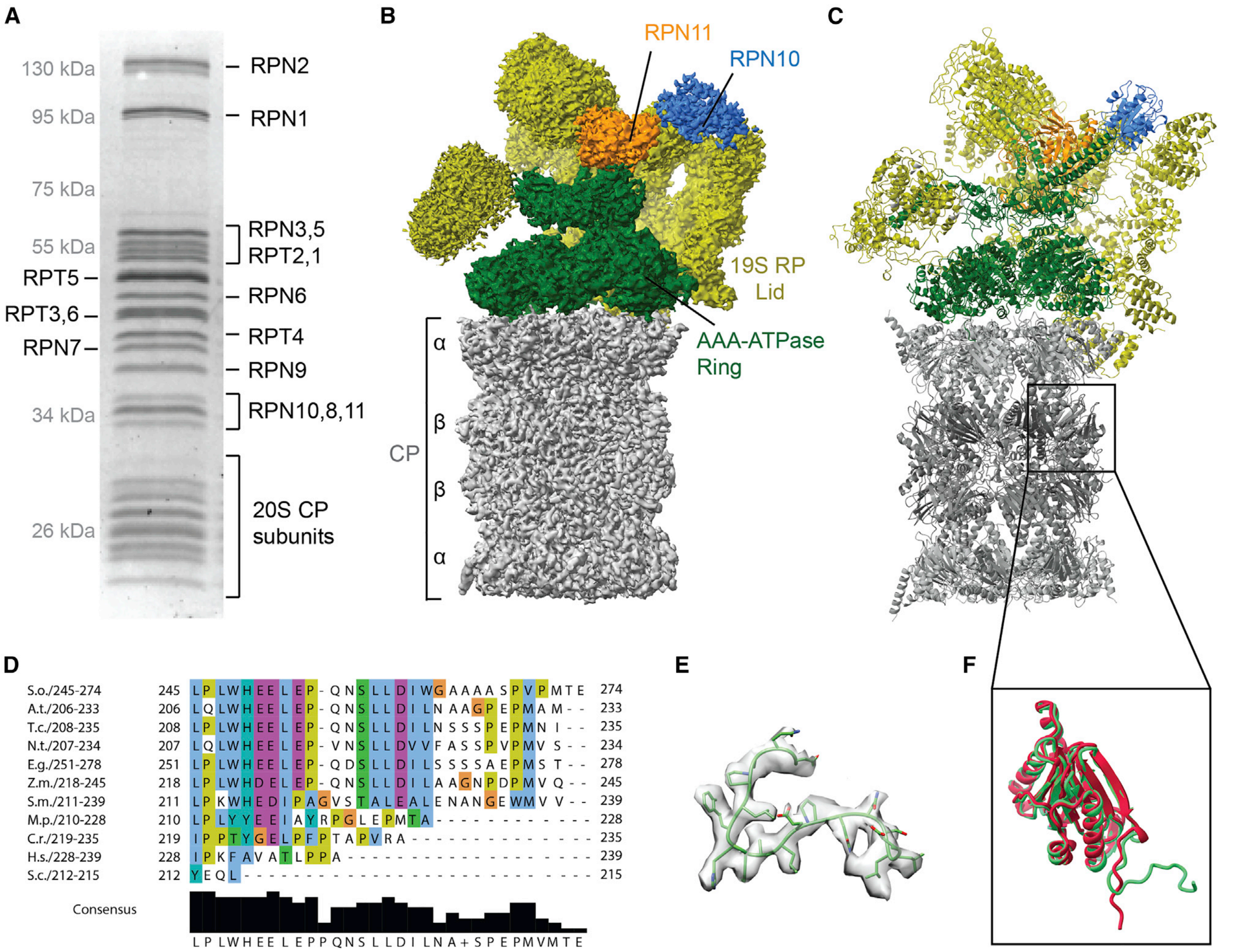

Figure 1. SDS–PAGE, cryo-EM map, and structure of the 26S proteasome from *Spinacia oleracea*.

(A) SDS–PAGE analysis of the spinach 26S proteasome. All subunits from the CP and the RP are visible as bands.

(B) The cryo-EM density of the 26S spinach proteasome is shown with its 19S RP lid subcomplex displayed in yellow, the RP base subcomplex in green, and the 20S CP in gray. The ubiquitin receptor RPN10 is colored blue, and the deubiquitinating enzyme subunit RPN11 is colored orange.

(C) For the atomic model, the same color code as in **(B)** was used.

(D) Alignment of the β1 C-termini of different organisms showing the divergence of the tail in higher plants, budding yeast, and human. S.o., *S. oleracea*; A.t., *Arabidopsis thaliana*; T.c., *Theobroma cacao*; N.t., *Nicotiana tabacum*; E.g., *Elais guineensis*; Z.m., *Zea mays*; S.m., *Selaginella moellendorffii*; M.p., *Marchantia polymorpha*; C.r., *Chlamydomonas reinhardtii*; H.s., *Homo sapiens*; S.c., *Saccharomyces cerevisiae*.

(E) β1 extension of the spinach proteasome from residue 205 overlaid with the density of the cryo-EM map.

(F) Overlay of the spinach (light green) and human (cherry red) β1 subunits. The black box points to the position of the subunit in the structure in **(C)**.

cognate pockets in the CP α-ring. The insertion of the C-terminus of RPT2 in the α3–α4 pocket follows during the lid rotation and deubiquitination procedure. During the ATPase cycle, additional insertion of the C-termini of the pseudo HbYX RPT1 and RPT6 into the lysine pockets at the interface of α2–α3 and α4–α5 completes gate opening and facilitates translocation. RPT3 does not promote gate opening but is instead more important for assembly of the 26S proteasome (Dong et al., 2019).

Conformational variability

To further compare the plant proteasome architecture to known structures, we analyzed the dataset for conformational variability of the 26S proteasome (Supplemental Figures 4 and 5). The main conformation of the protein is a presumably inactive conformational state, which is characterized by a misalignment of the AAA-subcomplex pores and the 20S gate (Bard et al., 2018). This so-called S_A state is also found in our high-resolution structure. We also found all previously described non-S_A states. These have varying degrees of rotation between the lid and the base of the RP and different tilt angles between the two subcomplexes. Because of the continuous nature of these non-S_A states, we could not classify them into discrete states and instead described them as a continuum and classified the different conformational modes.

Our analysis found 58% of the particles in the resting state and 42% in non-S_A states. As in all other species, the most significant movement is the clockwise rotation of the lid in relation to the ATPase with a pivot point close to RPN11. Consequently, at a

Accession no. (UniProt)	Protein	Peptides	Function
A0A0K9QNC1	CDC48/p97/VCP	19	chaperone
A0A0K9RJU4	ECM29	35	adaptor and scaffolding protein
A0A0K9QH09	PI31/PSMF1	5	proteasome inhibitor
A0A0K9R7D3	PSMD10	5	chaperone
A0A0K9RBE5	PSMD5	22	chaperone
A0A0K9R5W5	ubiquitin	3	–
A0A0K9RZ84	UBP6/USP14/TGT	12	deubiquitinating enzyme (DUB)
A0A0K9RL07	UCH2/UCH37/UCHL5	9	deubiquitinating enzyme (DUB)
A0A0K9RY40	UPL1-like/HUWE1/TOM1	26	E3 ligase
A0A0K9QUV5	UPL2-like/HUWE1	93	E3 ligase

Table 1. List of identified proteasome-associated proteins.

full rotation of 35°, RPN1 touches the coiled-coil formed by RPT4 and RPT5, and the entire ATPase is shifted to align the CP gate and the ATPase central channel. Second, we found a tilting (range 4°–10°) of the entire lid toward the ATPase with a pivot point in the free space between the ATPase and the lid, bringing the catalytic center of the deubiquitinating subunit RPN11 close to the entry point of the ATPase. Independent of the significant movements, we observed the free movement of RPN1, which can swing by 25 Å and rotate (Figure 3A–3D), similar to previously described movements of this subunit (Ding et al., 2019).

To our surprise, apart from the well-described conformations, we found an additional state that changes the CP rather than the RP (Supplemental Video 1A). A small yet significant subset of particles (7%) showed a 20S particle compressed by 12 Å that has less density on the non-RP-occupied gate (Figure 3). This flexibility of the free α-ring is reflected even in our high-resolution structure, where this α-ring is less resolved than the other parts of the structure (Supplemental Figure 5). We then constructed a conformational landscape using our previously published method (Haselbach et al., 2017, 2018). Analyzing the established landscape (Chen et al., 2016), we found the compression movement part of a pronounced continuous movement in which only the final fully compressed state is populated (Figure 3E). Surprisingly, in contrast to reports on the human and yeast conformational landscapes (Wu et al., 2020), the lid rotation is a less pronounced and sampled movement, showing only a few intermediate states. We found a considerable energy barrier of at least 5.6 k_BT between the extreme states in both cases.

To confirm that this new movement is not an artifact of our analysis method, we turned to two additional tools to validate our observation. These tools use very distinct approaches, either a common-line-based principal-component analysis (PCA) or a variational autoencoder-based method. All procedures found the sparsely populated states that showed an extra opening of the unoccupied side but with a less pronounced compression. This finding is unexpected, as the gate is thought to be closed at all times (Groll et al., 2000; Smith et al., 2007) to protect from unwanted degradation. One possible explanation could be that this is a quick burst movement to open the gate, leading to the release of the peptide product.

To understand the significance of this movement, we analyzed the proteasomes of other organisms. A similar movement was described previously for the archaeal proteasome of *Thermoplasma acidophilum* (T20S) (Punjani and Fleet, 2021). Examining published datasets of the human proteasome, we could not identify similar movements, which may reflect the low occupancy of this state. Conversely, analyzing bovine proteasomes with negative stain, we found a low population of class averages showing compressed 20S particles (Supplemental Video 1B), indicating that this movement may also exist in mammalian proteasomes. We speculate that this motion may be required for the release of peptide products. This function has been loosely attributed to the pores at the surface of the 20S. However, no study has performed further analysis to support this claim.

Proteasomal pores

We measured the diameters of potential pores of the plant, human, and yeast proteasomes (Figure 4). We found a total of 30 pores in spinach, 25 in human, and 12 in yeast, ranging in diameter from 4 to 17 Å (Supplemental Figure 6). By contrast, the opened gate in these species had a diameter of 14 Å. The largest transverse diameter of a peptide is given by the bulky amino acids (arginine, tryptophan, and tyrosine), suggesting that pores smaller than this will not be sufficient to release the products of a substrate. Only larger pores (>7 Å) that can let the bulky amino acids through can be considered for peptide release. Judging from the size of the pores alone, in principle, there are adequately sized pores in all proteasomes that would allow for the release of products. However, the question remains whether this is sufficient for the kinetics of the proteasome. The protease would need to release its products at least as fast as it translocates the substrate into the chamber. As these pores are only twice as big as the product diameter in the largest case, it is not clear that they would be sufficient for a fast release of peptides.

To estimate this, we followed a model calculation for diffusion through narrow pores (Sung and Park, 1996). Assuming that the pores are inert, peptide diffusion occurs as described by the formula $\sim \frac{L^2}{2D}$, where L is the length of the peptide and D is its diffusivity. This analysis demonstrated that short peptides

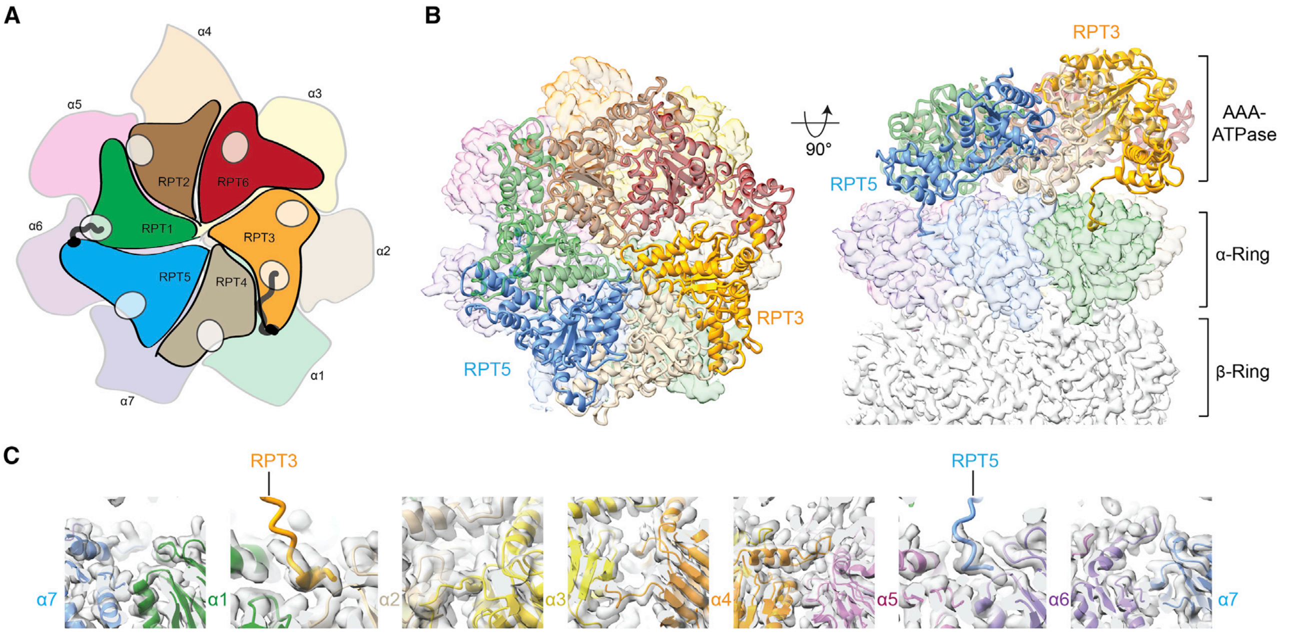

Figure 2. Interaction of two RPT N termini and their cognate α pockets in the resting state.
(A) Cartoon depicting the interaction of two subunits of the ATPase ring (solid colors) and the α ring (opaque colors). The pockets of the α ring are shown as opaque ovals. The N terminus of RPT5 (solid blue) is associated with the α5–α6 pocket, and the HbYX motif of RPT3 (solid orange) is interacting with the α1–α2 pocket.
(B) Tail–pocket interactions as determined by cryo-EM. UCSF Chimera software was used to visualize the interaction between the RPT3 and the RPT5 tails docked into their corresponding α pockets. For better orientation, the left shows the top view of the ATPase model and the α-ring density. The same color code was used as in **(A)**. The side view (right) shows the density of one β and α ring and the model of the AAA-ATPase, displaying the interaction of the N termini of RPT3 (solid orange) and RPT5 (solid blue) with the α ring.
(C) Closer views of the α pockets. Although there is a clear density for the N terminus of RPT5 (light blue) in the α5–α6 pocket and the N terminus of RPT3 (dark orange) in the α1–α2 pocket, there is no density visible in the other α pockets.

(L = 2) would diffuse through the pore four times slower, whereas long ones (L = 10) would move 100 times slower than their free diffusion in solution. The diffusion constant of a typical proteasome product has been estimated to be about 3 $\mu m^2/s$ (Reits et al., 2003), meaning that it would diffuse 1 nm (thickness of the pore) in 160 ns in solution and in 16 µs through the pore. To put this in context, the proteasome degrades a protein at a speed of 40 amino acids per second (Luciani et al., 2005; Sha et al., 2018). This means that the translocation speed is still four orders of magnitude slower than the possible release through the pores; thus, it is possible that these pores suffice. However, if the pore is not inert, meaning that the peptide is attracted to the inside of the pore, a massive slowdown would occur. Given the distribution of charge and hydrophobicity in the identified pores, we must assume that there is at least some interaction potential, which, however, we cannot quantify. In summary, the stochastic opening of the gate may facilitate peptide release but may not be strictly required.

DISCUSSION

The plant proteasome has been intensely characterized biochemically; however, structural insights have been lacking (Ozaki et al., 1992; Fujinami et al., 1994; Yang et al., 2004; Book et al., 2010).

Here, we present the first 3D structure of the plant 26S proteasome. Despite billions of years of evolutionary divergence, we could find only two significant differences between the proteasomes of higher plants and higher animals, highlighting the importance of this machine for all life. The main difference between plants and animals occurs at the C-terminus of the β1 subunit. Its high conservation in higher plants, as well as its evolutionary reinvention in several other organism groups, indicates its significance. As we could not find a change in the basal activity of the proteasome, we can only hypothesize that this well-structured extension may serve as a new binding or modification site.

Although we could resolve the 20S CP well, the resolution was limited in the 19S region. We speculate that this limitation is due to isoforms present in the preparation. Whereas yeast and human assemble a single 26S proteasome complex from a unigene set of RP and CP genes, most subunits of the plant protein are encoded by two genes. Mass spectrometry data (Supplemental Figure 7 and Supplemental Table 1) suggest that the proteasome is not a single particle, but a heterogeneous collection assembled using both paralogs of duplicated CP and RP subunits. Some of these pairs are nearly identical; others display enough divergence (e.g., RPN12) to suggest that different activities are possible (Yang et al., 2004; Book et al., 2010; Gemperline et al., 2019).

Our investigation of the complex dynamics showed the already known human and yeast conformations, such as the rotation and tilting of the lid and the movements of RPN1. We found these to be highly conserved; however, despite similar preparations, we

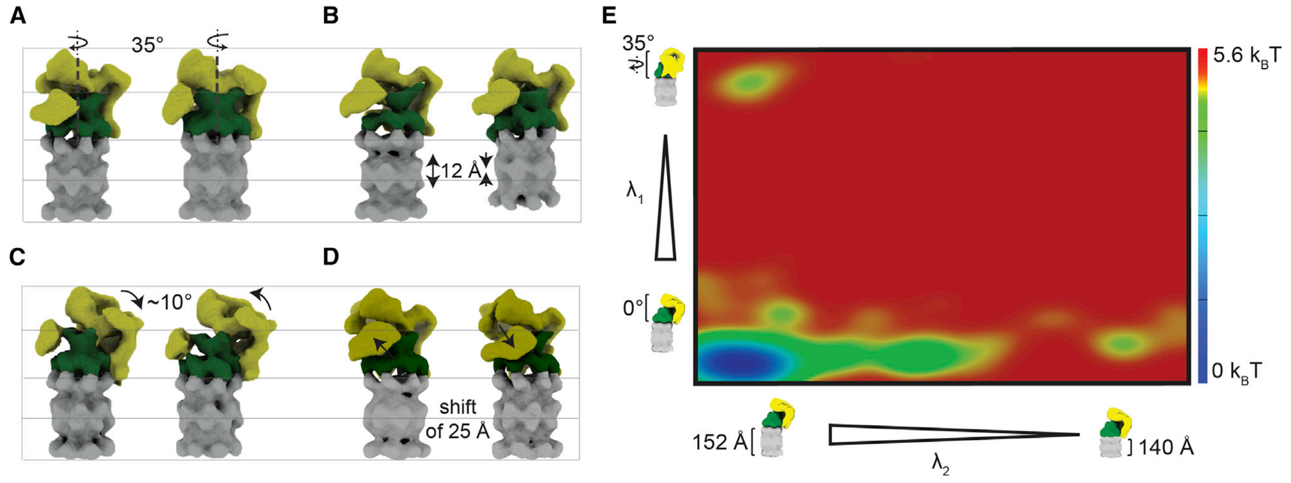

F

Modes		Pivot points	Amplitudes
Lid rotation	a	Interphase on RPN11	35°
Compression of 20S	b	Center of 20S	12 Å
Lid tilting	c	Between base and lid	4 - 10°
Moving of RPN1	d	Center of RPN1	range of 25 Å
Gate opening	g	Center of non-occupied α-ring	11 Å

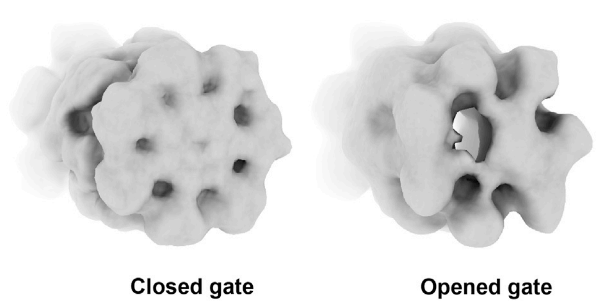

Figure 3. Most abundant trajectories of the spinach proteasome and energy landscape of the two most common conformations.
(A–D) Trajectories of the 26S proteasome generated via PCA (CowSuite). **(A)** shows the well-characterized lid rotation, **(B)** the newly observed compression movement. Tilting of the lid can be observed in **(C)**, and **(D)** shows the movement of RPN1, which can range between 1 and 25 Å.
(E) Conformational landscape of the spinach proteasome. The two most significant conformational modes are plotted as an energy landscape. The *y* axis denotes the rotation of the lid against the CP. The *xy* axis shows the newly described compression movement of the CP. Particle populations have been converted to energies by the Boltzman equation.
(F) List of modes found in the dataset, their pivot points, and amplitudes.
(G) Display of the opened gate on the unoccupied α-ring.

did not successfully define individual sets in our dataset but rather a continuum of movements, indicating different energetics.

In addition, we could identify a compression movement of the CP, in combination with a partially opened gate on the RP-distal α-ring. The gate opening and the overall more flexible RP may be consequences of a complex mechanism that plants require to cope with extreme environmental conditions, like temperature fluctuations.

We speculate that the observed stochastic gate opening may serve as an additional peptide release mechanism. How degradation products are released by the proteasome is still a mystery that has barely been investigated. A general suggestion in the field is the existence of pores in the 20S CP that suffice for peptide exit. Our findings suggest that pores do exist and are theoretically capable of releasing peptides. Assuming that this mechanism is the only way for peptides to exit the holoenzyme, the chemical nature of the pores suggests that this release step may be rate limiting, at least for some peptides. Therefore, the stochastic gate opening would be an elegant way to solve the issue and overcome the rate-limiting factor of the pores. Future studies are needed to characterize the phenomenon of peptide release from proteasome pores, which could be a detrimental part of the proteolytic capacities of the proteasome in general.

METHODS

Purification of 26S proteasomes from *Spinacia oleracea*

The original protocol described by Besche and Goldberg (2012) and Marshall et al. (2017) was slightly modified for the purification of 26S proteasomes from *S. oleracea*. Fresh spinach leaves ("Simply good" young spinach from the supermarket "Billa" in Austria) were frozen in liquid nitrogen and ground using a freezer/mill (SPEX SamplePrep). The following steps for the purification procedure were performed at 4°C on ice or in a cold room. The powder was resuspended at 2 g (fresh weight)/ml extraction buffer (EB; 25 mM Bis–Tris [pH 6.5], 50 mM KCl, 5 mM $MgCl_2$) freshly supplemented with 10% (w/v) glycerol, 20 mM ATP, 5 mM DTT, 5% (w/v) polyvinylpolypyrrolidone, and 2 mM phenylmethylsulfonyl fluoride. The debris was filtered through a stack of four layers of cheesecloth (Regency Naturals, Dallas, TX, USA) and two layers of Miracloth (Merck Millipore) and clarified by two ultracentrifugation steps, initially for 10 min at 30 000 *g* followed by 30 min at 100 000 *g* (Optima XE-90 ultracentrifuge, rotor 45TI, Beckman Coulter). The 26S proteasome was further purified by fractionated precipitation. Contaminating proteins were removed by gentle addition of polyethylene glycol 8000 (50% [w/v] stock [in EB] supplemented with 10 mM ATP) to a final concentration of

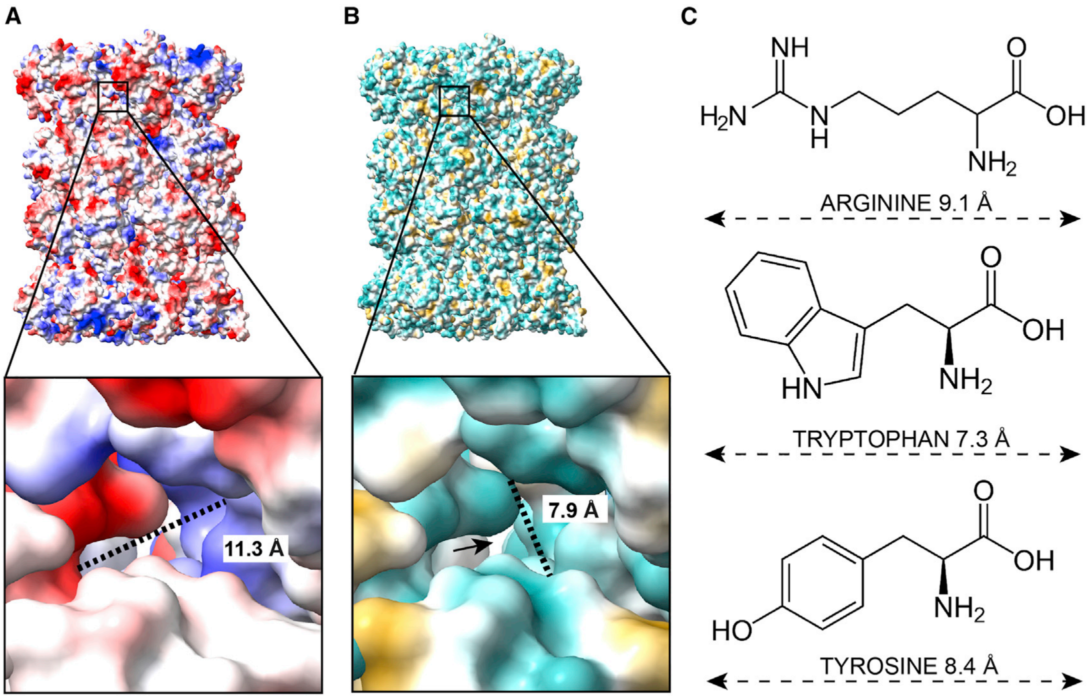

Figure 4. 20S proteasome pores.
(A) Electrostatic surface view of the 20S proteasome from *S. oleracea*. The pore shown measures 11.3 Å at its longest axis. Blue represents the positive and red the negative potential.
(B) Hydrophobic surface view of the same position as in **(A)**. Pore size in the smallest axis was measured to 7.9 Å. The arrow points to another pore on the opposite side of the holoenzyme. Here, the pore shown is a hydrophilic (green) rather than a hydrophobic (yellow) pore.
(C) Structural formula and size of the amino acids arginine, tryptophan, and tyrosine.

5% (v/v) under constant stirring and incubation for 15 min. Precipitated proteins were removed by centrifugation at 16 000 *g* for 15 min (Sorvall Lynx 6000 centrifuge, Thermo Scientific), and the desired protein was precipitated by increasing the polyethylene glycol concentration to 20% (v/v). Precipitated 26S proteasome was pelleted by centrifugation at 12 000 *g* for 15 min and dissolved in EB supplemented with 5% (w/v) glycerol, 10 mM ATP, and 5 mM DTT.

Approximately 3 mg of Ubl domain from Rad23a fused to GST (GST-Ubl, see below) was immobilized on 1 ml magnetic GST beads (MagneGST particles, Promega) equilibrated with EB. After overnight incubation at 4°C with dissolved proteasomes on a rotary wheel, the proteasome–Rad23a–Ubl–GST complex was eluted from the beads by the addition of 25 mM reduced glutathione (Roth). Finally, GST-Ubl was removed from the pure 26S proteasomes by sucrose gradient ultracentrifugation (10%–30% [w/v] sucrose in EB supplemented with 10 mM ATP and 5 mM DTT) in SW60 tubes (Seton) using a swinging bucket rotor (SW 60 Ti, Beckman Coulter). After centrifugation at 88 000 *g* (28 000 rpm) for 16 h, the gradient was manually fractionated to 200-μl aliquots, and final protein quality was monitored using SDS–PAGE and negative staining EM.

If not otherwise specified, the chemicals were provided by Sigma Aldrich.

Purification of HBTH-tagged Rpn11 26S proteasomes from HEK293 GP cells

Frozen cells in resuspension buffer (25 mM Bis–Tris [pH 6.5], 50 mM KCl, 5 mM $MgCl_2$, 0.1% NP-40, 10% glycerol) were thawed in a water bath at 37°C after addition of 4 mM ATP, 1 mM DTT, 0.1 mM phenylmethylsulfonyl fluoride, and a 2500× dilution of benzonase. After the cells were lysed with a Dounce homogenizer, they were centrifuged at 19 000 rpm for 45 min at 4°C (Sorvall Lynx 6000 centrifuge, Thermo Scientific). The supernatant was then ultracentrifuged for 1 h at 100 000 *g* and 4°C (Optima XE-90 ultracentrifuge, Beckman Coulter). After filtration through a double layer of Miracloth (Merck Millipore) followed by a 0.45-μm filter, a HiTrap streptavidin 1-ml column (GE Healthcare) and a GST Trap FF 1-ml column (GE Healthcare) were used for purification. The proteasome complexes were bound to the streptavidin column equilibrated with buffer A (EB with 10% [w/v] glycerol, 1 mM DTT, and 4 mM ATP). The bound proteins were first washed with buffer A supplemented with 150 mM NaCl and cleaved off from the column by application of GST-TEV protease and incubation overnight at 4°C. The proteasomes were then eluted with 1× EB with freshly added 10% (w/v) glycerol, 0.5 mM Tris(2-carboxyl)phosphine (TCEP), and 4 mM ATP. The fractions containing purified 26S proteasomes were identified via SDS–PAGE.

If not otherwise specified, the chemicals were provided by Sigma Aldrich.

Cloning and purification of Rad23a-Ubl-GST

Cloning and purification of GST-Ubl were performed according to standard procedures. The codon region spanning the Ubl domain of Rad23a (residues 1–88) was amplified from a synthetic gene (Thermo Fisher GeneArt) and cloned into the pGEX vector by Gibson assembly. The plasmid encoding GST-Ubl was transformed into *Escherichia coli* strain BL21(DE3). Overnight cultures were inoculated with single colonies, cells were grown in autoinduction medium (ZY medium [tryptone, yeast extract, and ddH_2O] with 20× P [100 mM PO_4, 25 mM $(NH_4)_2SO_4$], 50× 5052 [0.5% glycerol, 0.05% glucose, 0.2% α-lactose], 0.001 mM $MgSO_2$, and antibiotics) at 37°C for 5.5 h and subsequently cooled to 18°C, and growth resumed overnight. Bacteria were harvested by centrifugation, and the pellet was resuspended in 1× phosphate-buffered saline (PBS; KCl, KH_2PO_4, NaCl, Na_2HPO_4, 10 mM $MgCl_2$, 2 mM DTT, and 1 μl benzonase).

Cell walls were broken with a cell disrupter (Constant Systems, UK), and cell debris was cleared by centrifugation. The supernatant was loaded onto a 5-ml GST trap column (GE Healthcare) equilibrated with 1× PBS supplemented with 2 mM DTT. Unbound proteins were removed by intensive washing with 1× PBS, and GST-Ubl was eluted with 1× PBS supplemented with 2 mM DTT and 10 mM reduced glutathione. Finally, the protein was polished by size exclusion using HiLoad 16/600 Superdex 75 pg (Sigma Aldrich) equilibrated with 1× PBS. All steps were monitored by SDS–PAGE, and the protein was concentrated to 5 mg/ml, frozen in liquid nitrogen, and stored at −80°C.

If not otherwise specified, the chemicals were provided by Sigma Aldrich.

Substrate production and purification

Full length securin* was expressed in BL21(DE3) Codon Plus (RIL) cells, purified, and fluorescently labeled (denoted by an asterisk) as previously described (Jarvis et al., 2016). Polyubiquitinated securin was generated enzymatically by mixing 10 μM substrate, 0.1 μM APC/C, 1 μM CDH1, 5 μM UBCH10, 1 μM E1 (Uba1), 100 μM ubiquitin, and 10 mM Mg-ATP. After 1.5 h at room temperature, the reactions were quenched with 50 mM EDTA (pH 8.0) and flash frozen.

To purify the substrate from the ubiquitin ligase machinery for the activity assay, 1.6 μl NaAc (pH 4.0) was added and incubated on ice for 30 min. After centrifugation, the supernatant was carefully taken without disrupting the pellet, and the pellet was then dissolved in 1× PBS and dialyzed in 1× EB with 10% glycerol.

In vitro degradation assay

Proteasomal activity was characterized using ub_n-securin*. Freshly purified plant proteasomes (30 nM) were incubated with 10-fold (300 nM) substrate protein in 200 μl buffer (25 mM Tris [pH 7.5], 5 mM $MgCl_2$, 5% glycerol [w/v]) freshly supplemented with 5 mM ATP and 1 mM DTT at 25°C under constant agitation for 20 h. Aliquots were withdrawn after 0, 10, 30, 60, 90, 120, and 180 min and 20 h of incubation. The reaction was quenched by denaturation using SDS sample buffer (final 1×), separated by stain-free SDS–PAGE (Criterion TGX stain-free precast gel 4%–20%, 26 wells, Bio Rad; running conditions 180 V for 40 min) and imaged using a ChemiDoc MP imaging system (Bio-Rad). Because of the fluorescently labeled substrate securin, deubiquitination and degradation by the proteasome could be visualized using the fluorescein channel of the ChemiDoc system. As a control, 2 mM MG132 (a proteasome inhibitor that blocks the proteolytic activity of the proteasome; MedChemExpress) was used to show the inhibited proteasome. The same reactions were also performed with human proteasomes (purified from HEK293 cells) at 37°C.

Figures showing the degradation assay (Supplemental Figure 1) were edited to remove scratches from the scanner surface in the background using the Spot Healing Brush Tool in Photoshop (2022).

Mass spectrometry

Nano-LC–MS analysis

The nano-high performance liquid chromatography system used was an UltiMate 3000 RSLCnano system (Thermo Fisher Scientific, Amsterdam, the Netherlands) coupled to a Q Exactive HF mass spectrometer (Thermo Fisher Scientific, Bremen, Germany) equipped with a Proxeon nanospray source (Thermo Fisher Scientific, Odense, Denmark). Peptides were loaded onto a trap column (Thermo Fisher Scientific, Amsterdam, the Netherlands; PepMap C18, 5 mm × 300 μm i.d., 5-μm particles, 100-Å pore size) at a flow rate of 25 μl/min using 0.1% Trifluoroacetic acid (TFA) as the mobile phase. After 10 min, the trap column was switched in line with the analytical column (Thermo Fisher Scientific, Amsterdam, the Netherlands; PepMap C18, 500 mm × 75 μm i.d., 2 μm, 100 Å). Peptides were eluted using a flow rate of 230 nl/min and a binary 4-h gradient, respectively 260 min.

The gradient began with the mobile phase 98% A (water/formic acid, 99.9/0.1, v/v) and 2% B (water/acetonitrile/formic acid, 19.92/80/0.08, v/v/v), increased to 35% B over the next 240 min, increased to 90% B over 5 min, remained there for 5 min, and decreased back to 98% A and 2% B in 5 min for equilibration at 30°C.

The Q Exactive HF mass spectrometer was operated in data-dependent mode, using a full scan (*m/z* range 380–1500, nominal resolution of 60 000, target value 1 × 10^6) followed by tandem mass spectrometry (MS/MS) scans of the 10 most abundant ions. MS/MS spectra were acquired using a normalized collision energy of 27%, isolation width of 1.4 *m/z*, and resolution of 30 000, and the target value was set to 1 × 10^5. Precursor ions selected for fragmentation (excluding charge states 1, 7, 8, >8) were put on a dynamic exclusion list for 60 s. In addition, the minimum AGC target was set to 5 × 10^3, and the intensity threshold was calculated to be 4.8 × 10^4. The peptide match feature was set to preferred, and the exclude isotopes feature was enabled.

Data processing protocol

For peptide identification, the RAW files were loaded into Proteome Discoverer (version 2.1.0.81; Thermo Scientific). All created MS/MS spectra were searched using MS Amanda v.2.0.0.9849, Engine v.2.0.0.9849 (Dorfer et al., 2014). The RAW files were searched against the *Arabidopsis* genome database TAIR (33 038 sequences; 14 616 625 residues) and the UniProt database using the taxonomy *S. oleracea* (23 985 sequences; 9 454 539 residues). The following search parameters were used: iodoacetamide derivative on cysteine was set as a fixed modification, and oxidation on methionine, deamidation on asparagine and glutamine, acetylation on lysine, phosphorylation on serine, and threonine and tyrosine were set as variable modifications. Monoisotopic masses were searched within unrestricted protein masses for tryptic enzymatic specificity. The peptide mass tolerance was set to ±5 ppm and the fragment mass tolerance to ±15 ppm. The maximum number of missed cleavages was set to 2. The result was filtered to 1% false discovery rate on protein level using the Percolator algorithm (Käll et al., 2007) as integrated in Proteome Discoverer. The localization of the post-translational modification sites within the peptides was performed with the ptmRS tool based on the phosphoRS tool (Taus et al., 2011). Peptide areas were quantified using the in-house-developed tool apQuant (Doblmann et al., 2018).

Bioinformatics: Sequence retrieval

To collect spinach orthologs, we performed NCBI blast searches using sequences of the *Arabidopsis thaliana*, *Saccharomyces cerevisiae*, and *Homo sapiens* proteasomes against a set of *S. oleracea* proteins that were downloaded from NCBI (58 427 entries, status 04/2018) (Altschul et al., 1997). Hits were selected for the lowest E value and aligned with MAFFT (Katoh and Toh, 2008). Paralogous gene families, such as the α or β core proteasome subunits or the ATPase regulatory subunits RPT1 to RPT6, were aligned, including all paralogs. The respective orthologs were assigned in an neighbour-joining (NJ) phylogenetic tree with SeaView (Gouy et al., 2010). To study the C-terminal conservation of RPT1 to RPT6, we extracted full-length orthologs from the NCBI or UniProt sequence databases, aligned them with MAFFT, and visualized the alignment with Jalview (Waterhouse et al., 2009). Putative sequence fragments were excluded. For a graphical representation of the alignments, residues were colored using the Clustal X coloring scheme.

Negative staining

Four microliters of the sample was applied to a carbon-coated grid and incubated for 30 s to 1 min, depending on the concentration of the sample. The grid was blotted, washed two times with ddH_2O, and stained with uranyl acetate for 1 min (Hoppert, 2003). Samples were imaged on an FEI Technai T20 microscope at a magnification of 60 000×, corresponding to a pixel size of 1.85 Å per pixel with an Eagle 4k HS camera.

Preparation of cryo grids

A GraFix gradient (Stark, 2010) was used to stabilize the complex for single-particle cryo-EM. A 10%–30% sucrose gradient was prepared as described previously with 0.05% (v/v) glutaraldehyde added to the 30% sucrose buffer and quenched with 5 mM aspartate (pH 7.3) during fractionation. The peak fractions containing the 26S proteasomes were identified by SDS–PAGE and negative staining EM. Sucrose was removed from the sample prior to vitrification by buffer exchange (Zeba Spin Desalting columns, Thermo Scientific). The particles were subsequently absorbed to a continuous carbon film attached to a Quantifoil (3.5/1) 200 mesh grid and plunge frozen with a Leica EM GP.

Electron cryo microscopy data acquisition

The cryo grid was imaged in a 300-kV FEI Titan Krios transmission electron microscope, and images were taken at a nominal magnification of 75 000, resulting in a pixel size of 1.058 Å per pixel on a Falcon 3D detector. Two datasets were collected with a total dose of 80 electrons/A^2, and the total dose of the third dataset was fractionated on 50 electrons/A^2. In total, 22 858 micrographs were collected.

Data processing

Image frames were aligned and weighted according to electron dose using the software MotionCor2 (Zheng, 2016), followed by contrast transfer function (CTF) determination using gCTF (Zhang, 2016). The micrographs were sorted using CowSuite's Quality Checker (unpublished results), and bad micrographs (blurred, contaminated, or empty) were discarded. Two thousand three hundred eleven particles were manually picked on the left 10 027 micrographs using Relion (Zivanov et al., 2019). With the class averages from Relion, Gautomatch (http://www.mrc-lmb.cam.ac.uk/kzhang/) was able to pick 1 779 876 particles. Through the 2D classification, the best classes could be chosen to generate an initial 3D model, using it as reference in a 3D classification in Relion. By alignment and comparison of 3D classifications in UCSF Chimera (Pettersen et al., 2004), it was possible to distinguish different conformations within the dataset. PCAs in CowSuite (https://www.cow-em.de), cryoDRGN (Zhong et al., 2021), and CryoSPARC2 (Punjani et al., 2017) were used as additional tools (Supplemental Tables 2 and 3).

Model building

Homology models for the individual proteasomal chains were generated by CHAINSAW (a program for mutating PDB files used as templates in molecular replacement) (Stein, 2008) using the human 26S proteasome (6msb) model and sequence alignments produced by Clustal X (Jeanmougin et al., 1998). Non-conserved residues were pruned to their Cβ atom. The model for the human 26S proteasome was placed into the EM density using Chimera, followed by real-space rigid-body refinement as a single entity. The individual chains from homology modeling were placed by superposition in Coot (Emsley and Cowtan, 2004) and further corrected by individual rigid-body refinement of single polypeptide chains. Pruned side chains were corrected and placed manually, and an initial model was made by real-space refinement in Coot. The chains were further fitted to the map by real-space refinement using PHENIX (Liebschner et al., 2019), except those for which the map was ambiguous and did not permit further fitting (subunits of the lid).

For those subunits, we generated α fold models (Jumper et al., 2021) truncated to poly(Ala) using the PDB tool in PHENIX and then rigid-body fitted them into the map. Model quality was assessed using MolProbity (Williams et al., 2018). The final model proved to have good stereochemistry, with 93.81% and 94.94% residues (20S and 19S) in the favored region of the Ramachandran plot and 0.13% and 0.23% outliers. Figures were produced using ChimeraX (Pettersen et al., 2021) (Supplemental Tables 2 and 3).

ACCESSION NUMBERS

The EM density map has been deposited in the EMDB with accession no. EMD-14175. RAW micrographs have been uploaded to the EMPIAR database (EMPIAR-10974). Modeled atomic coordinates have been deposited in the Protein Data Bank with accession nos. 7QVE (20S) and 7QVG (19S). The materials and data that support the findings of this study are available from the corresponding authors upon request.

SUPPLEMENTAL INFORMATION

Supplemental information can be found online at *Plant Communications Online*.

FUNDING

D.L.B. and N.G.B. are supported by NIH R35GM128855 and the University Cancer Research Fund (UCRF). H.D.T. and B.F. were supported by NIH grant R01GM125769. The IMP, the whole Haselbach lab, and especially S.K. are supported by Boehringer Ingelheim.

AUTHOR CONTRIBUTIONS

S.K. performed most of the experiments under the supervision of I.G. and guidance of D.H. I.G. executed the cloning and gave general biochemical support. K.B. helped to establish degradation assays for the plant system. S.A. provided help with the assays and substrates. A.M. supervised and supported model building. N.G.B. and D.L.B. performed ubiquitination assays and provided ubiquitinated substrates. A.S. implemented bioinformatic sequence analysis and annotated spinach subunits. K.M. and R.I. provided proteomic analysis. B.F., H.D.T., and E.D.Z. contributed to the analysis of conformational states. S.K. and D.H. wrote the manuscript with the support of all other authors.

ACKNOWLEDGMENT

The support for Nicholas G. Brown, Derek L. Bolhuis, Zuzana Hodakova, and Katarina Belačić in writing the manuscript is greatly appreciated. We want to thank Yasin Dagdas and Peter Schlögelhofer for their support, suggestions, and fruitful discussions. Thanks to Jutta Dammann and Ana Ramos Velázquez for support with experiments, Anneliese Auer for help regarding growing plants, and Juraj Ahel for computational assistance. We wish to show our appreciation to the Clausen and Matoushek Lab for providing reagents. We also want to thank Holger Stark for cryoelectron microscopy measurement time at MPI BPC Göttingen. Any other assistance with measurement and data collection was provided by the EM Facility of the Vienna BioCenter Core Facilities GmbH (VBCF), a member of the Vienna BioCenter (VBC), Austria. No conflict of interest is declared.

Received: January 7, 2022
Revised: February 1, 2022
Accepted: March 4, 2022
Published: May 9, 2022

REFERENCES

Altschul, S.F., Madden, T.L., Schäffer, A.A., Zhang, J., Zhang, Z., Miller, W., and Lipman, D.J. (1997). Gapped BLAST and PSI-BLAST: a new generation of protein database search programs. Nucleic Acids Res. **25**:3389–3402.

Bard, J.A.M., Goodall, E.A., Greene, E.R., Jonsson, E., Dong, K.C., and Martin, A. (2018). Structure and function of the 26S proteasome. Annu. Rev. Biochem. **87**:697–724.

Bard, J.A.M., Bashore, C., Dong, K.C., and Martin, A. (2019). The 26S proteasome utilizes a kinetic gateway to prioritize substrate degradation. Cell **177**:286–298.e15.

Besche, H.C., and Goldberg, A.L. (2012). Affinity purification of mammalian 26S proteasomes using an ubiquitin-like domain. In Ubiquitin Family Modifiers and the Proteasome: Reviews and Protocols, Methods in Molecular Biology (Springer), pp. 423–432.

Book, A.J., Gladman, N.P., Lee, S.-S., Scalf, M., Smith, L.M., and Vierstra, R.D. (2010). Affinity purification of the Arabidopsis 26 S proteasome reveals a diverse array of plant proteolytic complexes. J. Biol. Chem. **285**:25554–25569.

Chen, S., Wu, J., Lu, Y., Ma, Y.-B., Lee, B.-H., Yu, Z., Ouyang, Q., Finley, D.J., Kirschner, M.W., and Mao, Y. (2016). Structural basis for dynamic regulation of the human 26S proteasome. Proc. Natl. Acad. Sci. U S A **113**:12991–12996.

Ding, Z., Xu, C., Sahu, I., Wang, Y., Fu, Z., Huang, M., Wong, C.C.L., Glickman, M.H., and Cong, Y. (2019). Structural snapshots of 26S proteasome reveal tetraubiquitin-induced conformations. Mol. Cell **73**:1150–1161.e6.

Doblmann, J., Dusberger, F., Imre, R., Hudecz, O., Stanek, F., Mechtler, K., and Dürnberger, G. (2018). apQuant: accurate label-free quantification by quality filtering. J. Proteome Res. **18**:535–541. https://doi.org/10.1021/acs.jproteome.8b00113.

Dong, Y., Zhang, S., Wu, Z., Li, X., Wang, W.L., Zhu, Y., Stoilova-McPhie, S., Lu, Y., Finley, D., and Mao, Y. (2019). Cryo-EM structures and dynamics of substrate-engaged human 26S proteasome. Nature **565**:49–55.

Dorfer, V., Pichler, P., Stranzl, T., Stadlmann, J., Taus, T., Winkler, S., and Mechtler, K. (2014). MS amanda, a universal identification algorithm optimized for high accuracy tandem mass spectra. J. Proteome Res. **13**:3679–3684.

Eisele, M.R., Reed, R.G., Rudack, T., Schweitzer, A., Beck, F., Nagy, I., Pfeifer, G., Plitzko, J.M., Baumeister, W., Tomko, R.J., et al. (2018). Expanded coverage of the 26S proteasome conformational landscape reveals mechanisms of peptidase gating. Cell Rep. **24**:1301–1315.e5.

Emsley, P., and Cowtan, K. (2004). Coot : model-building tools for molecular graphics. Acta Crystallogr. Section D Biol. Crystallogr. **60**:2126–2132.

Finley, D., Tanaka, K., Mann, C., Feldmann, H., Hochstrasser, M., Vierstra, R., Johnston, S., Hampton, R., Haber, J., Mccusker, J., et al. (1998). Unified nomenclature for subunits of the Saccharomyces cerevisiae proteasome regulatory particle. Trends Biochem. Sci. **23**:244–245.

Fu, H., Girod, P.A., Doelling, J.H., Van Nocker, S., Hochstrasser, M., Finley, D., and Vierstra, R.D. (1999). Structure and functional analyses of the 26S proteasome subunits from plants Plants. Mol. Biol. Rep. **26**:137–146.

Fujinami, K., Tanahashi, N., Tanaka, K., Ichihara, A., Cejka, Z., Baumeister, W., Miyawaki, M., Sato, T., and Nakagawa, H. (1994). Purification and characterization of the 26S proteasome from spinach leaves. J. Biol. Chem. **269**:25905–25910.

Gemperline, D.C., Marshall, R.S., Lee, K.-H., Zhao, Q., Hu, W., McLoughlin, F., Scalf, M., Smith, L.M., and Vierstra, R.D. (2019). Proteomic analysis of affinity-purified 26S proteasomes identifies a suite of assembly chaperones in Arabidopsis. J. Biol. Chem. **294**:17570–17592.

Gouy, M., Guindon, S., and Gascuel, O. (2010). SeaView version 4: a multiplatform graphical user interface for sequence alignment and phylogenetic tree building. Mol. Biol. Evol. **27**:221–224.

Groll, M., Bajorek, M., Köhler, A., Moroder, L., Rubin, D.M., Huber, R., Glickman, M.H., and Finley, D. (2000). A gated channel into the proteasome core particle. Nat. Struct. Biol. **7**:1062–1067.

Haselbach, D., Schrader, J., Lambrecht, F., Henneberg, F., Chari, A., and Stark, H. (2017). Long-range allosteric regulation of the human 26S proteasome by 20S proteasome-targeting cancer drugs. Nat. Commun. **8**:1–8.

Haselbach, D., Komarov, I., Agafonov, D.E., Hartmuth, K., Graf, B., Dybkov, O., Urlaub, H., Kastner, B., Lührmann, R., and Stark, H. (2018). Structure and conformational dynamics of the human spliceosomal bact complex. Cell **172**:454–464.e11.

Hoppert, M. (2003). Microscopic Techniques in Biotechnology (Wiley).

Jarvis, M.A., Brown, N.G., Watson, E.R., VanderLinden, R., Schulman, B.A., and Peters, J.M. (2016). Measuring APC/C-dependent ubiquitylation in vitro. In Cell Cycle Oscillators (Springer), pp. 287–303.

Jeanmougin, F., Thompson, J.D., Gouy, M., Higgins, D.G., and Gibson, T.J. (1998). Multiple sequence alignment with Clustal X. Trends Biochem. Sci. **23**:403–405.

Jumper, J., Evans, R., Pritzel, A., Green, T., Figurnov, M., Ronneberger, O., Tunyasuvunakool, K., Bates, R., Žídek, A., Potapenko, A., et al. (2021). Highly accurate protein structure prediction with AlphaFold. Nature **596**:583–589.

Käll, L., Canterbury, J.D., Weston, J., Noble, W.S., and MacCoss, M.J. (2007). Semi-supervised learning for peptide identification from shotgun proteomics datasets. Nat. Methods **4**:923–925.

Katoh, K., and Toh, H. (2008). Recent developments in the MAFFT multiple sequence alignment program. Brief. Bioinform. **9**:286–298.

Köhler, A., Cascio, P., Leggett, D.S., Woo, K.M., Goldberg, A.L., and Finley, D. (2001). The axial channel of the proteasome core particle is gated by the Rpt2 ATPase and controls both substrate entry and product release. Mol. Cell **7**:1143–1152.

Lander, G.C., Estrin, E., Matyskiela, M.E., Bashore, C., Nogales, E., and Martin, A. (2012). Complete subunit architecture of the proteasome regulatory particle. Nature **482**:186–191.

Li, W., Bengtson, M.H., Ulbrich, A., Matsuda, A., Reddy, V.A., Orth, A., Chanda, S.K., Batalov, S., and Joazeiro, C.A.P. (2008). Genome-wide and functional annotation of human E3 ubiquitin ligases identifies MULAN, a mitochondrial E3 that regulates the organelle's dynamics and signaling. PLoS One **3**:e1487.

Liebschner, D., Afonine, P.V., Baker, M.L., Bunkóczi, G., Chen, V.B., Croll, T.I., Hintze, B., Hung, L.-W., Jain, S., McCoy, A.J., et al. (2019). Macromolecular structure determination using X-rays, neutrons and electrons: recent developments in Phenix. Acta Crystallogr. Section D Struct. Biol. **75**:861–877.

Luciani, F., Keşmir, C., Mishto, M., Or-Guil, M., and De Boer, R.J. (2005). A mathematical model of protein degradation by the proteasome. Biophys. J. **88**:2422–2432.

Marshall, R.S., Gemperline, D.C., and Vierstra, R.D. (2017). Purification of 26S proteasomes and their subcomplexes from plants. In Isolation of Plant Organelles and Structures: Methods and Protocols, Methods in Molecular Biology, N.L. Taylor and A.H. Millar, eds. (Springer New York), pp. 213–232.

Opoku-Nsiah, K.A., de la Pena, A.H., Williams, S.K., et al. (2022). The YΦ motif defines the structure-activity relationships of human 20S proteasome activators. Nat. Commun. **13**:1226. https://doi.org/10.1038/s41467-022-28864-x.

Ozaki, M., Fujinami, K., Tanaka, K., Amemiya, Y., Sato, T., Ogura, N., and Nakagawa, H. (1992). Purification and initial characterization of the proteasome from the higher plant Spinacia oleracea. J. Biol. Chem. **267**:21678–21684.

Peth, A., Nathan, J.A., and Goldberg, A.L. (2013). The ATP costs and time required to degrade ubiquitinated proteins by the 26 S proteasome. J. Biol. Chem. **288**:29215–29222.

Pettersen, E.F., Goddard, T.D., Huang, C.C., Couch, G.S., Greenblatt, D.M., Meng, E.C., and Ferrin, T.E. (2004). UCSF Chimera - a visualization system for exploratory research and analysis. J. Comput. Chem. **25**:1605–1612.

Pettersen, E.F., Goddard, T.D., Huang, C.C., Meng, E.C., Couch, G.S., Croll, T.I., Morris, J.H., and Ferrin, T.E. (2021). UCSF ChimeraX: structure visualization for researchers, educators, and developers. Protein Sci. **30**:70–82.

Punjani, A., and Fleet, D.J. (2021). 3D variability analysis: resolving continuous flexibility and discrete heterogeneity from single particle cryo-EM. J. Struct. Biol. **213**:107702.

Punjani, A., Rubinstein, J.L., Fleet, D.J., and Brubaker, M.A. (2017). cryoSPARC: algorithms for rapid unsupervised cryo-EM structure determination. Nat. Methods **14**:290–296.

Raab, M.S., Podar, K., Breitkreutz, I., Richardson, P.G., and Anderson, K.C. (2009). Multiple myeloma. Lancet **374**:324–339.

Reits, E., Griekspoor, A., Neijssen, J., Groothuis, T., Jalink, K., van Veelen, P., Janssen, H., Calafat, J., Drijfhout, J.W., and Neefjes, J. (2003). Peptide diffusion, protection, and degradation in nuclear and cytoplasmic compartments before antigen presentation by MHC class I. Immunity **18**:97–108.

Sha, Z., Zhao, J., and Goldberg, A.L. (2018). Measuring the overall rate of protein breakdown in cells and the contributions of the ubiquitin-proteasome and autophagy-lysosomal pathways. In The Ubiquitin Proteasome System: Methods and Protocols, T. Mayor and G. Kleiger, eds. (Springer New York), pp. 261–276.

Shi, Y., Chen, X., Elsasser, S., Stocks, B.B., Tian, G., Lee, B.-H., Shi, Y., Zhang, N., de Poot, S.A.H., Tuebing, F., et al. (2016). Rpn1 provides adjacent receptor sites for substrate binding and deubiquitination by the proteasome. Science **351**:aad9421.

Smith, D.M., Chang, S.-C., Park, S., Finley, D., Cheng, Y., and Goldberg, A.L. (2007). Docking of the proteasomal ATPases' carboxyl termini in the 20S proteasome's α ring opens the gate for substrate entry. Mol. Cell **27**:731–744.

Stark, H. (2010). GraFix: Stabilization of Fragile Macromolecular Complexes for Single Particle Cryo-EM (Elsevier Masson SAS).

Stein, N. (2008). CHAINSAW : a program for mutating pdb files used as templates in molecular replacement. J. Appl. Crystallogr. **41**:641–643.

Sung, W., and Park, P.J. (1996). Polymer translocation through a pore in a membrane. Phys. Rev. Lett. **77**:783–786.

Taus, T., Köcher, T., Pichler, P., Paschke, C., Schmidt, A., Henrich, C., and Mechtler, K. (2011). Universal and confident phosphorylation site localization using phosphoRS. J. Proteome Res. **10**:5354–5362.

Trujillo, M., and Shirasu, K. (2010). Ubiquitination in plant immunity. Curr. Opin. Plant Biol. **13**:402–408.

Unverdorben, P., Beck, F., Led, P., Schweitzer, A., Pfeifer, G., Plitzko, J.M., Baumeister, W., and Forster, F. (2014). Deep classification of a large cryo-EM dataset defines the conformational landscape of the 26S proteasome. Proc. Natl. Acad. Sci. U S A **111**:5544–5549.

Vierstra, R. (2003). The ubiquitin/26S proteasome pathway, the complex last chapter in the life of many plant proteins. Trends Plant Sci. **8**:135–142.

Vierstra, R.D. (1993). Protein degradation in plants. Annu. Rev. Plant Physiol. Plant Mol. Biol. **44**:385–410.

Waterhouse, A.M., Procter, J.B., Martin, D.M.A., Clamp, M., and Barton, G.J. (2009). Jalview Version 2--a multiple sequence alignment editor and analysis workbench. Bioinformatics **25**:1189–1191.

Williams, C.J., Headd, J.J., Moriarty, N.W., Prisant, M.G., Videau, L.L., Deis, L.N., Verma, V., Keedy, D.A., Hintze, B.J., Chen, V.B., et al. (2018). MolProbity: more and better reference data for improved all-atom structure validation. Protein Sci. **27**:293–315.

Wu, Z., Zhang, S., Wang, W.L., Ma, Y., Dong, Y., and Mao, Y. (2020). Deep manifold learning reveals hidden dynamics of proteasome autoregulation. Preprint at arXiv, 2012.12854. https://doi.org/10.48550/arXiv.2012.12854.

Xu, F., and Xue, H. (2019). The ubiquitin-proteasome system in plant responses to environments. Plant Cell Environ. **42**:2931–2944.

Yang, P., Fu, H., Walker, J., Papa, C.M., Smalle, J., Ju, Y.-M., and Vierstra, R.D. (2004). Purification of the Arabidopsis 26S proteasome. J. Biol. Chem. **279**:6401–6413.

Zhang, K. (2016). Gctf: real-time CTF determination and correction. J. Struct. Biol. **193**:1–12.

Zheng, S. (2016). MotionCor2 User Manual (Unviersity of California San Francisco).

Zhong, E.D., Bepler, T., Berger, B., and Davis, J.H. (2021). CryoDRGN: reconstruction of heterogeneous cryo-EM structures using neural networks. Nat. Methods **18**:176–185.

Zivanov, J., Nakane, T., and Scheres, S.H.W. (2019). A Bayesian approach to beam-induced motion correction in cryo-EM single-particle analysis. IUCrJ **6**:5–17.

Plant Communications, Volume *3*

Supplemental information

Cryo-EM structure of the plant 26S proteasome

Susanne Kandolf, Irina Grishkovskaya, Katarina Belačić, Derek L. Bolhuis, Sascha Amann, Brent Foster, Richard Imre, Karl Mechtler, Alexander Schleiffer, Hemant D. Tagare, Ellen D. Zhong, Anton Meinhart, Nicholas G. Brown, and David Haselbach

Supplemental information

Cryo-EM structure of the plant 26S proteasome

Susanne Kandolf, Irina Grishkovskaya, Katarina Belačić, Derek L. Bolhuis, Sascha Amann, Brent Foster, Richard Imre, Karl Mechtler, Alexander Schleiffer, Hemant Tagare, Ellen D. Zhong, Anton Meinhart, Nicholas G. Brown & David Haselbach

Supplemental Figures

Figure S1. SDS PAGE analysis of proteasomal degradation with polyubiquitinated human securin.

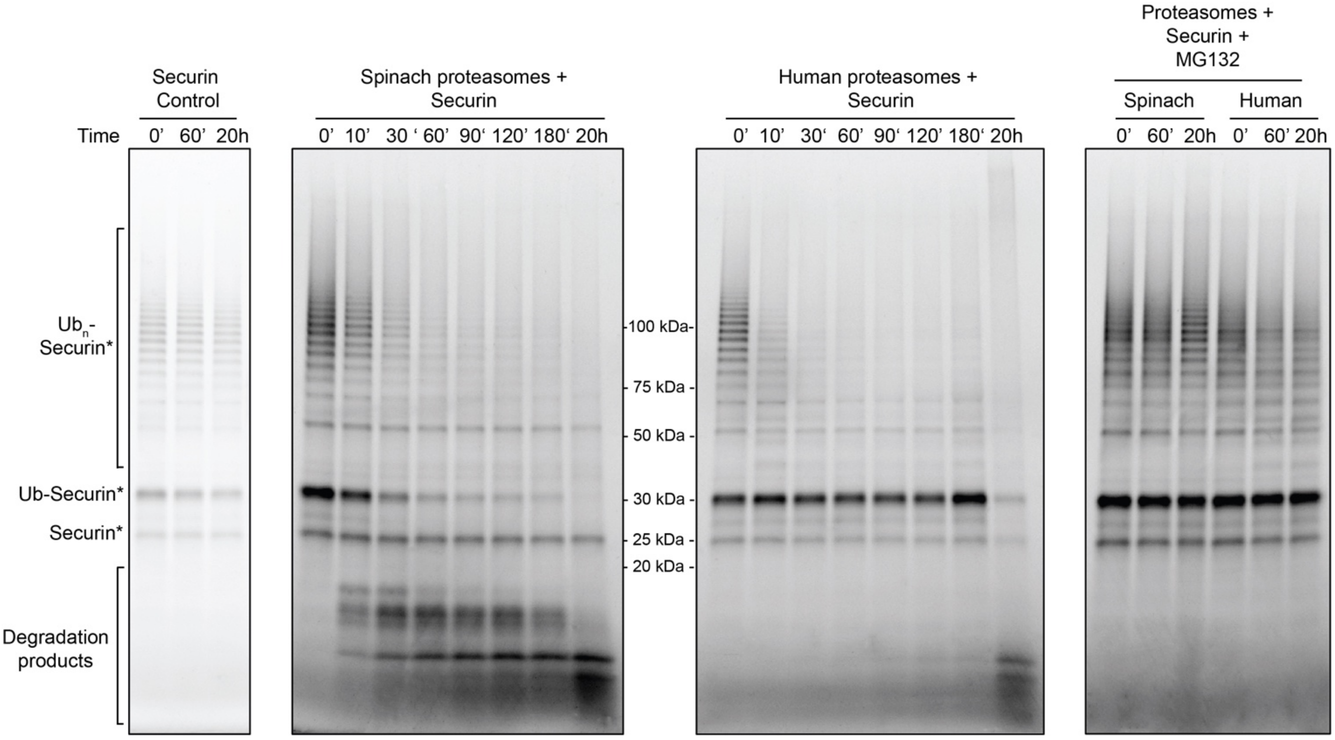

The bands ranging from 50-150 kDa represent securin with varying sizes of polyubiquitin chains (Ub_n-Securin*), the band corresponding to 30 kDa represents monoubiqutinated securin (Ub-Securin*) and the band corresponding to free securin is visualized at 25 kDa. The bands with sizes smaller than 25 kDa represent the degradation products. In reactions with spinach and human proteasomes, the disappearance of the Ub_n-Securin* bands points to the deubiquitination activities, while the appearance of degradation products confirms the degradation activity of the complexes. The presence of MG132 (proteasome inhibitor) abolishes degradation and coupled deubiquitination activities of both, spinach and human 26S proteasomes.

The image shows fluorescent signal derived from fluorescein maleimide labeled securin.

Figure S2. Overview of the subunit homology of *S. oleracea* compared to *A. thaliana*, *H. sapiens* und *S. cerevisiae.*

A

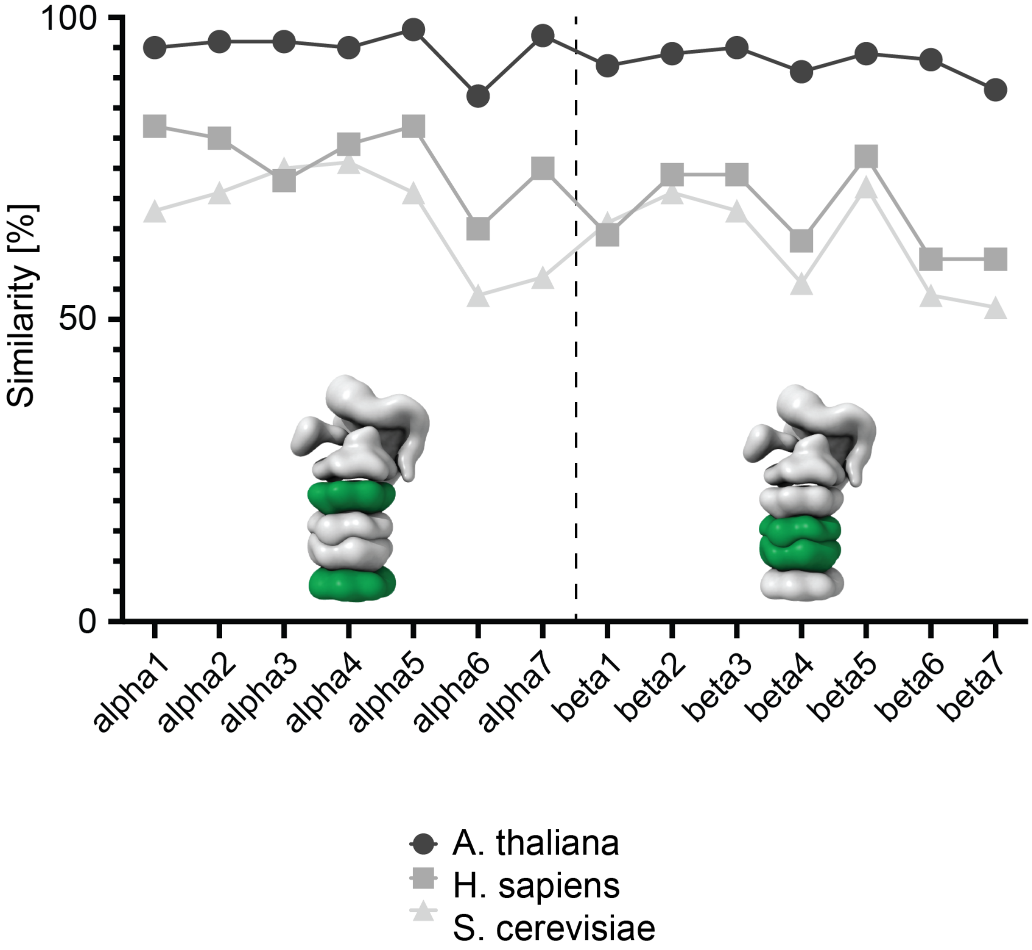

B

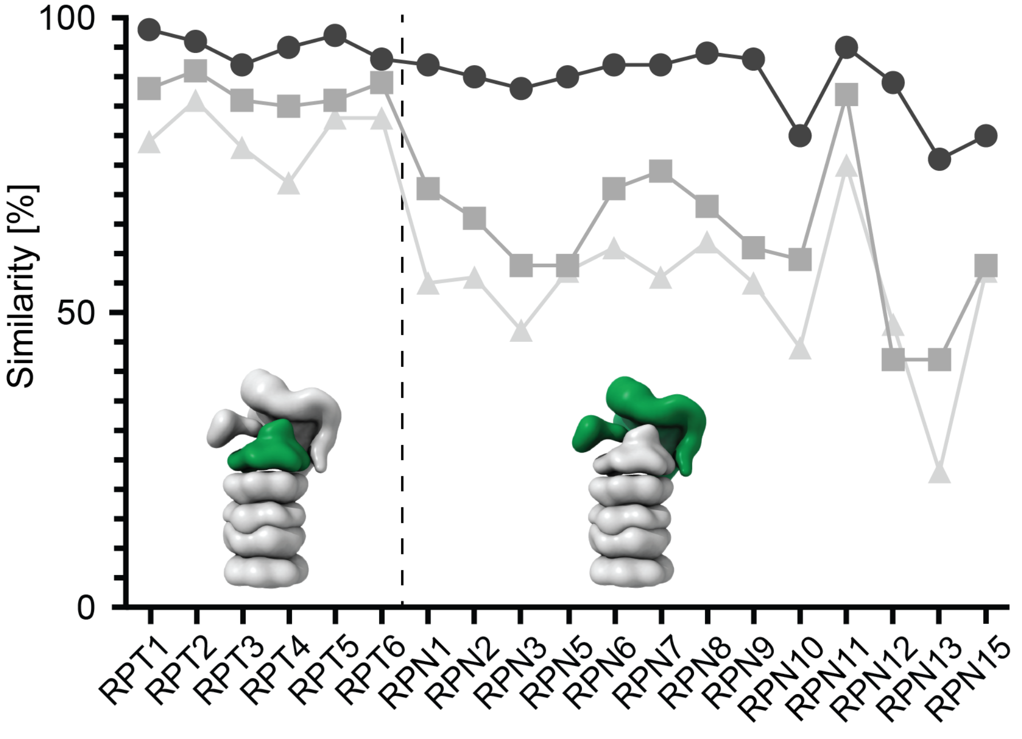

(A) Similarity of the ring subunits from spinach compared to the subunits of *A. thaliana*, mammalian, and budding yeast. As expected, the similarity of the 20S between spinach and *A. thaliana* lies above 90%, comparing spinach and human, it is roughly above 60%, and yeast is at 60%. **(B)** Lid and base subunit comparison of *A. thaliana, H. sapiens and S. cerevisiae*. The base of the proteasome seems quite conserved, while the lid differs significantly. Except the DUB RPN11, the similarity of the subunits of the lid lies at 80% for *A. thaliana*, at 60% for *H. sapiens* and at 50% for *S. cerevisiae*.

Figure S3. Derived amino acid sequence alignment - termini of HbYX motifs of the AAA-ATPase subunits compared to different organisms. Legend: *S.o. - Spinacia oleracea, A.t. - Arabidopsis thaliana, C.r. - Chlamydomonas reinhardtii, S.c. - Saccharomyces cerevisiae, D.m. - Drosophila melanogaster, H.s. - Homo sapiens.*

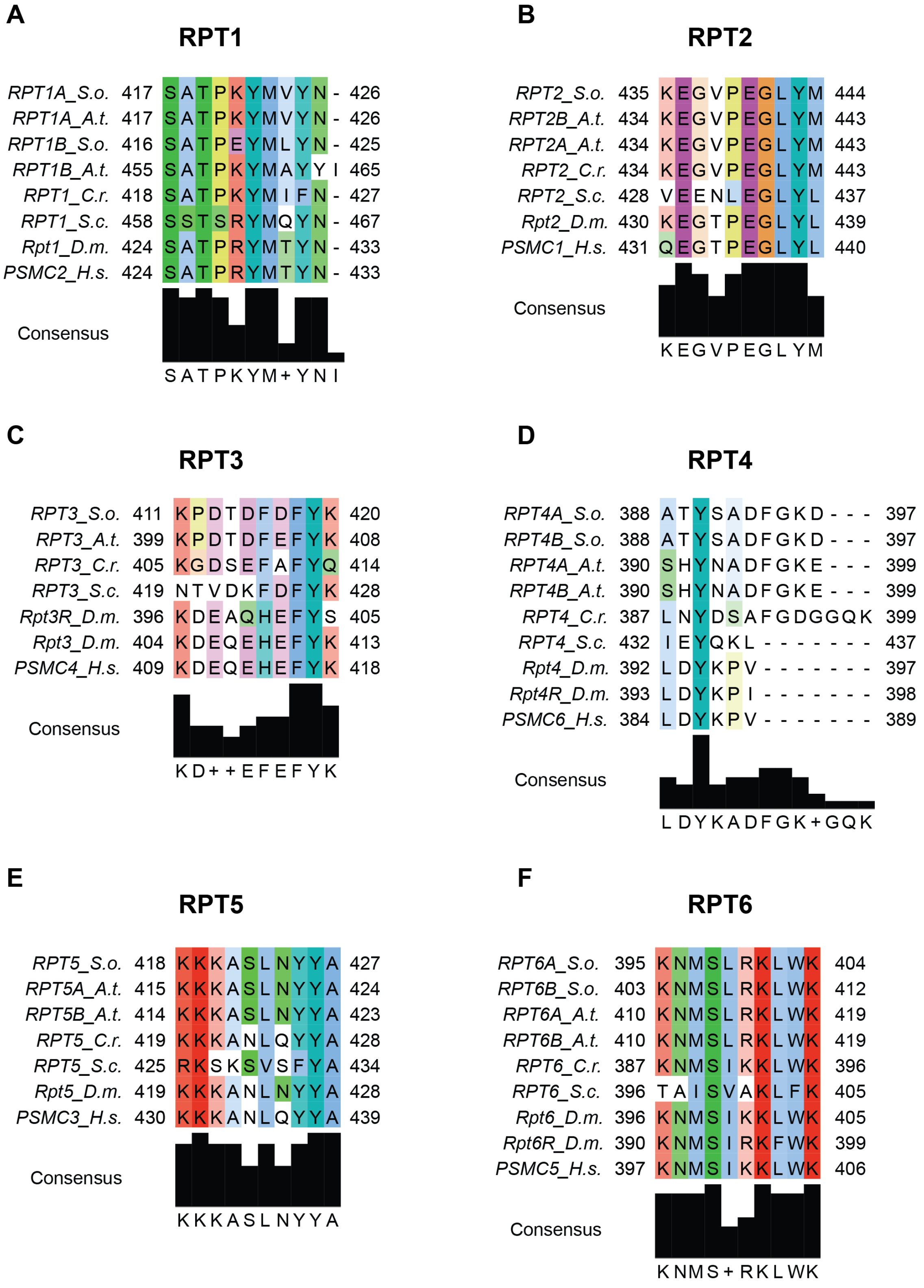

(A) – (F) Alignments of each RPT subunit C-terminus of the 26S proteasome. The sequence of subunits is relatively conserved, except for RPT1. Plants appear to have an additional HbYX motif - besides RPT2, 3 and 5 - compared to mammals.

Figure S4. Workflow of cryo-EM data processing.

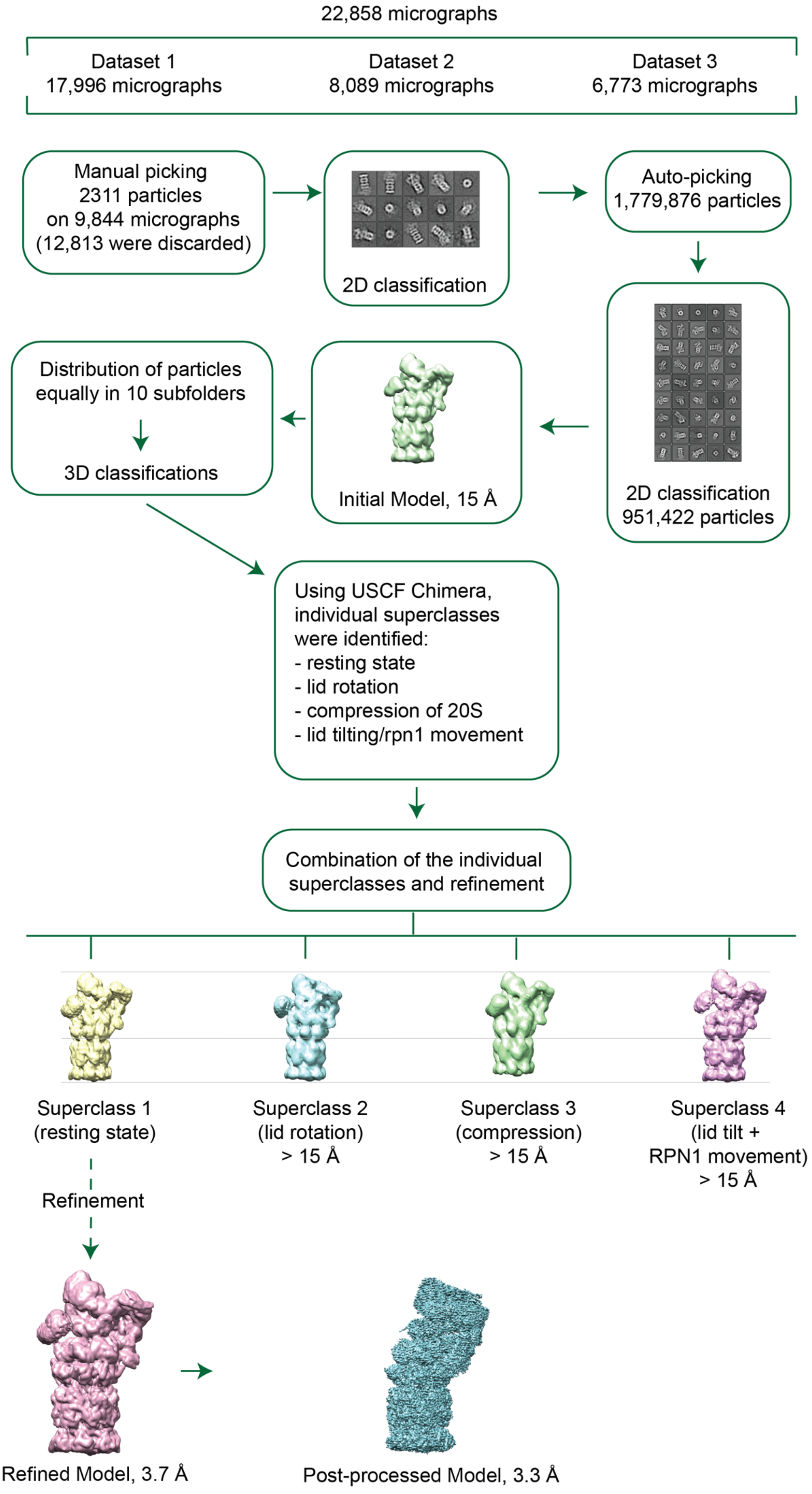

After collecting cryo-EM data, the micrographs were first checked for quality. After using the MQC (micrograph quality checker, CowSuite) to discard contaminated or bad micrographs, manual and auto-picking lead to a reasonable initial model. Due to 3D classification and the help of UCSF Chimera, it was possible to have a first look into the dynamics of the proteasome. Superclass 1 showed the resting state of the protein, Superclass 2 could be identified as the well-known lid rotation, Superclass 4 showed a series of lid tilting and RPN1 movements. Superclass 3 was a new conformation, that has not yet been described. Surprisingly, this was a motion of the 20S, rather than the RP. Together with a compression movement a partly gate opening of the non-occupied α-ring could be observed. The best 3D classes of each superclass were then combined and refined. While Superclass 1 led to a refined model with a resolution of 3.3 Å, the other superclasses reached a reasonable resolution to distinguish the different conformations, but not enough for further investigation.

Figure S5. Cryo-EM analysis.

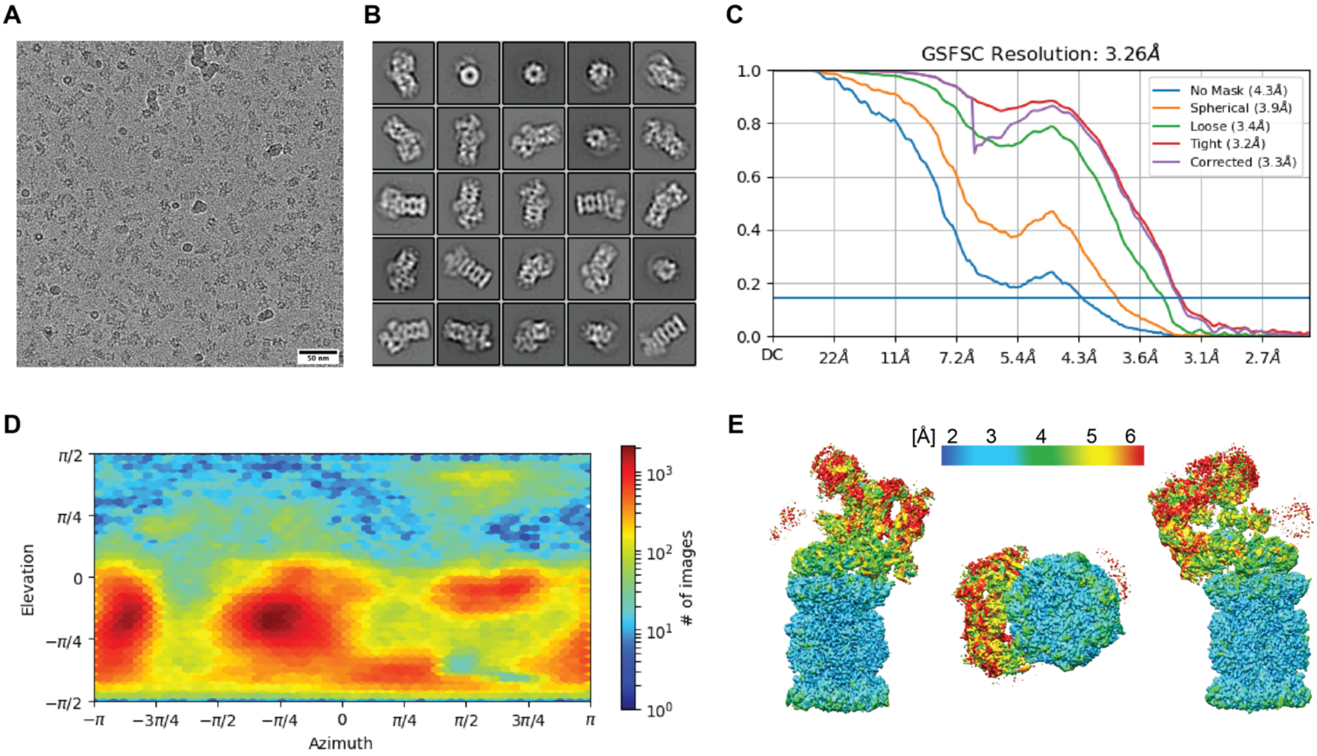

(A) A typical raw electron micrograph of the spinach 26S proteasomes embedded in vitreous ice. **(B)** Representative 2D class averages used for auto-picking. **(C)** Fourier shell correlation (FSC) curve. Average resolution of the reconstruction with C_1 symmetry, estimated to be 3.26 Å based on the gold-standard FSC criterion of 0.143. **(D)** Angle distribution of the refined map is shown. **(E)** In this local resolution map, each part of the density is colored according to the local resolution as specified in the color bar. The resolution ranges from 2 Å (blue) to 6 Å (red).

Figure S6. Pores of the spinach 26S proteasome.

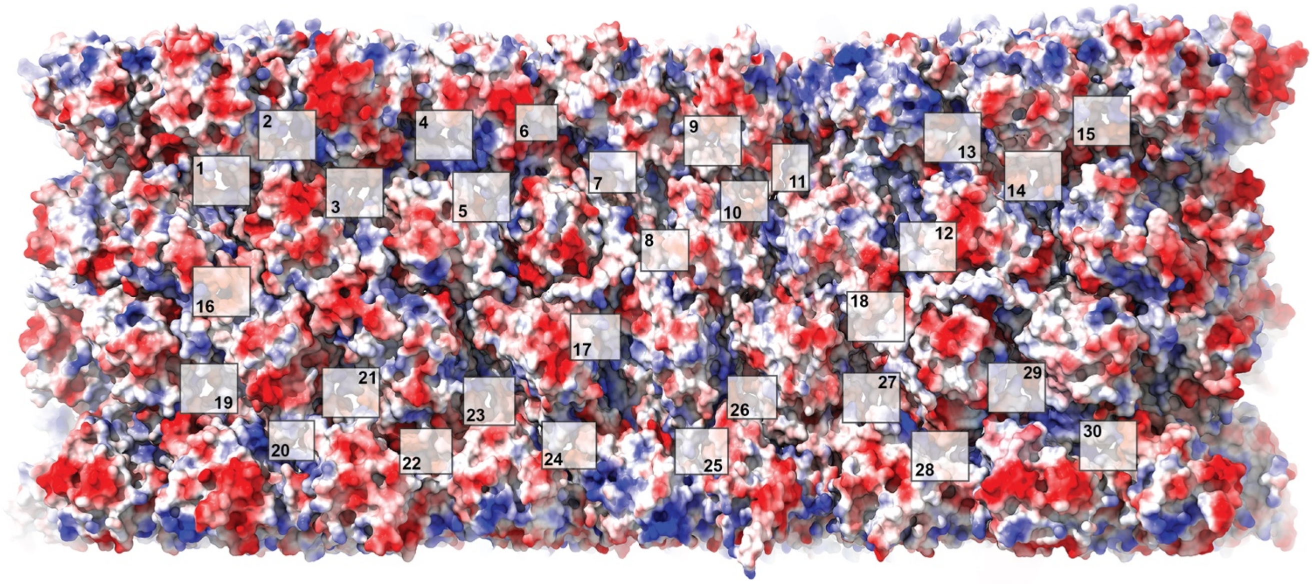

The panorama view shows the pores as an overview. The table contains the surrounding subunits, the longest and shortest axis, and a close-up snapshot of each pore. The numbering corresponds to the panorama view.

Pore Nr.	Surrounding subunits	Longest diameter [Å]	Shortest diameter [Å]	
1	α7 - β1 - β7	8.4	5.1	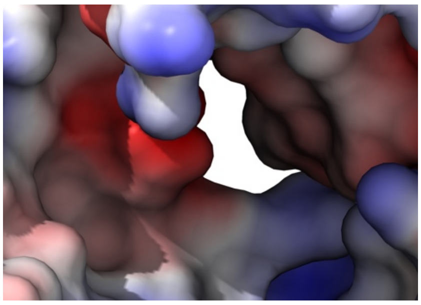
2	α1 - α7 - β1	9.3	3.6	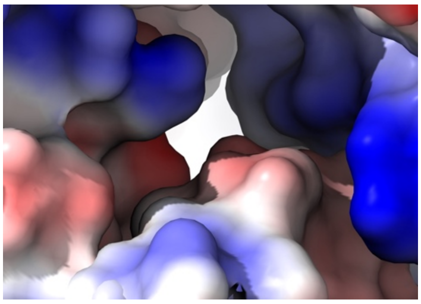

3	α1 - β1 - β2	16.1	8.4	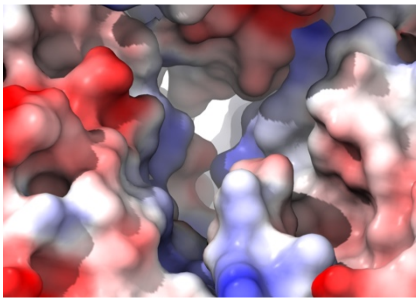
4	α1 - α2 - β2	16.9	7.7	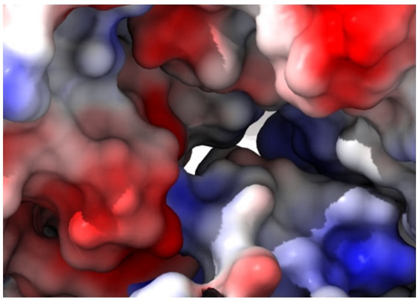
5	α2 - β2 - β3	13.3	3.3	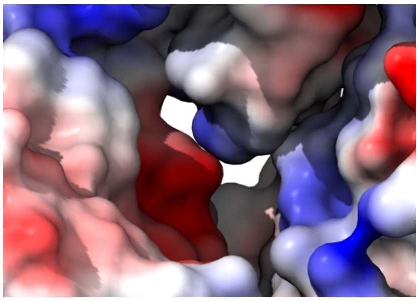
6	α2 - α3 - β3	11.8	6.7	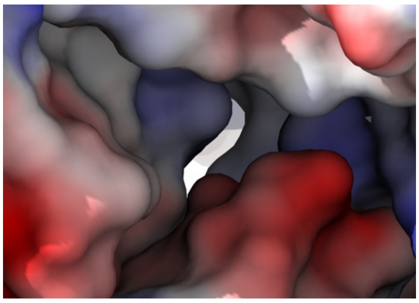
7	α3 - β3 - β4	8.2	5.7	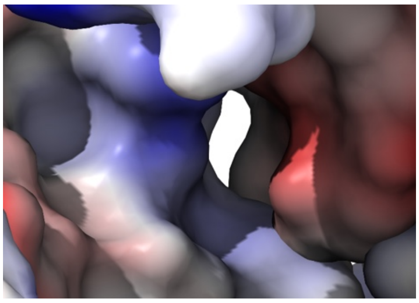

8	β3 - β4 - β5	8.9	8.2	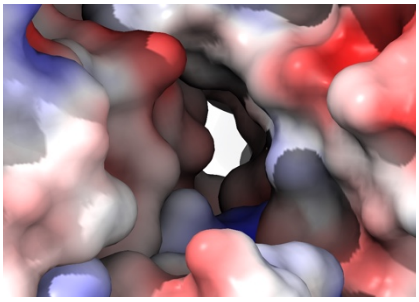
9	α3 - α4 - β4	11.1	5.1	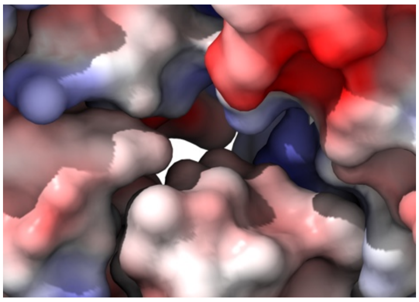
10	α4 - β4 - β5	14.0	3.4	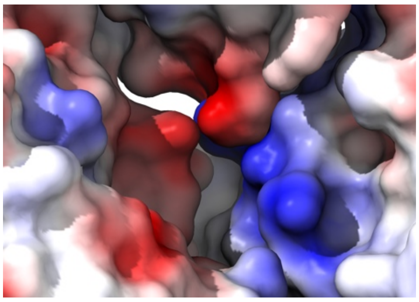
11	α4 - α5 - β5	13.4	5.4	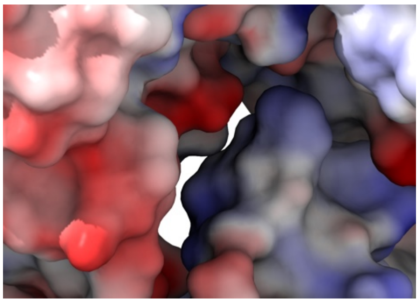
12	β5 - β6	10.0	6.5	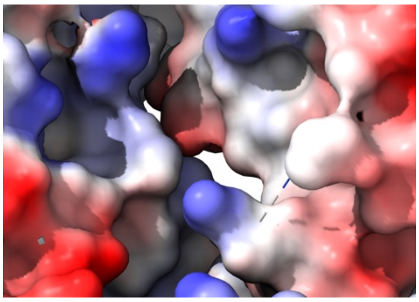

13	α6 - α5 - β6	8.3	7.7	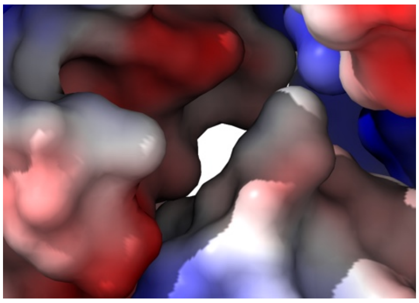
14	α6 - β6 - β7	8.9	5.9	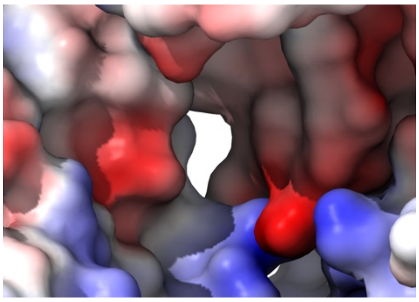
15	α6 - α7 - β7	9.8	9.2	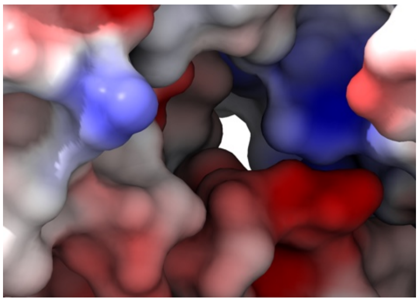
16	β1 - β7	4.2	4.1	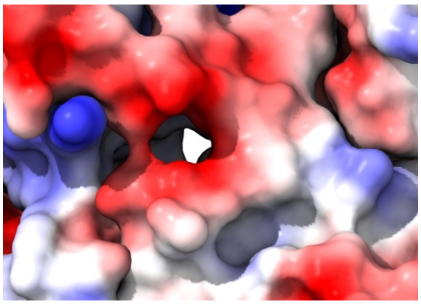
17	β5 - β6	9.6	6.0	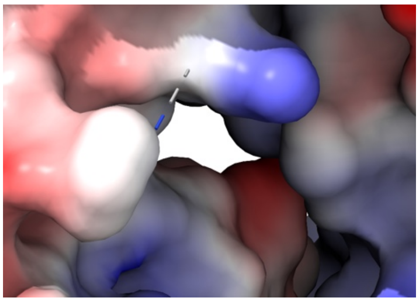

18	β3 - β4 - β5	6.4	6.1	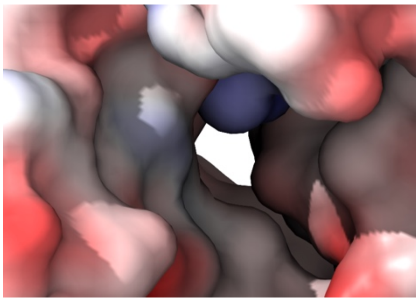
19	α1 - β1 - β2	6.5	5.4	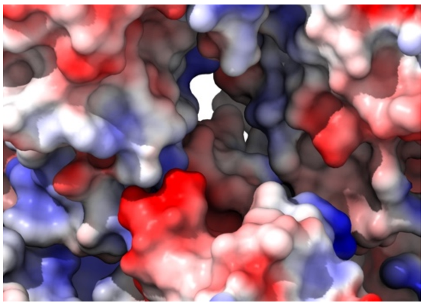
20	α1 - α7 - β1	8.6	7.7	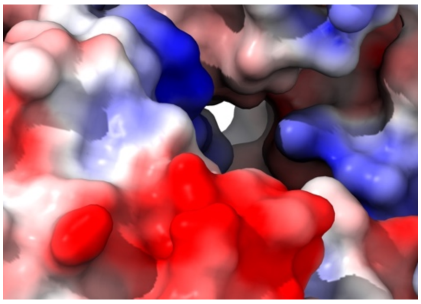
21	α7 - β1 - β7	11.7	14.7	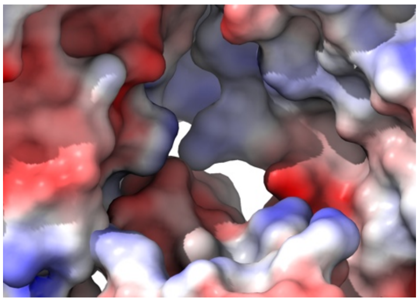
22	α6 - α7 - β7	11.7	11.1	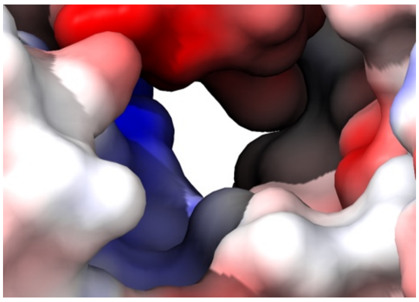

23	α6 - β6	8.4	5.6	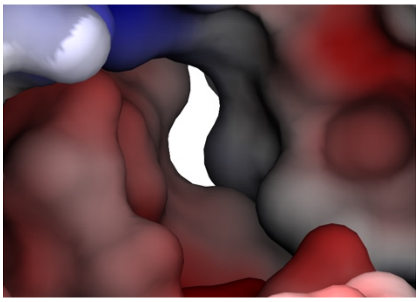
24	α5 - α6 - β6	6.9	5.0	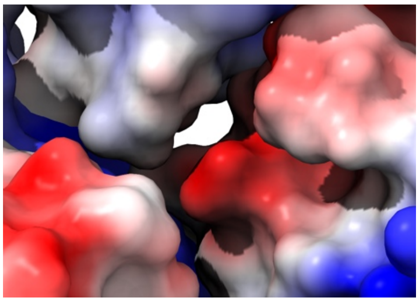
25	α4 - α5 - β5	16.9	4.8	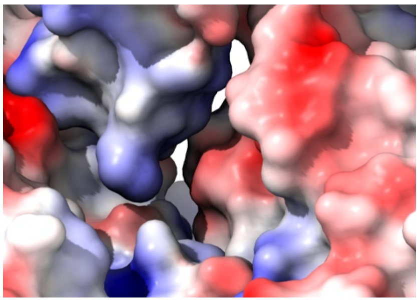
26	α4 - β4 - β5	14.0	6.1	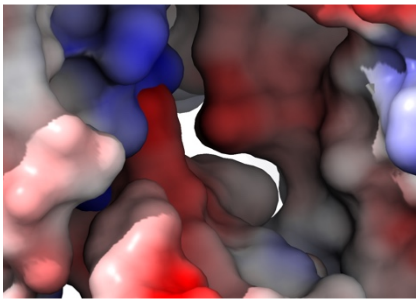
27	α3 - β3 - β4	8.4	7.3	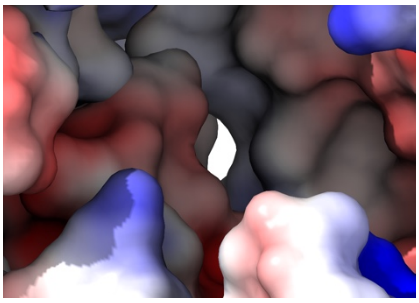

28	$\alpha 2$ - $\alpha 3$ - $\beta 3$	10.9	10.8	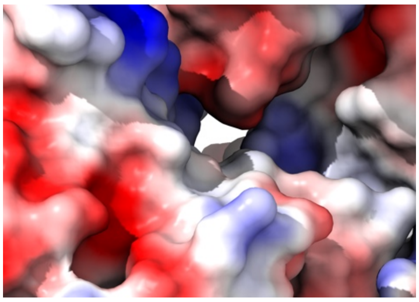
29	$\alpha 2$ - $\beta 2$ - $\beta 3$	17.5	4.5	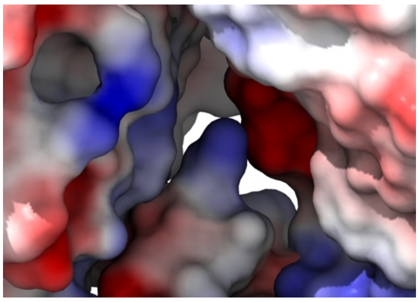
30	$\alpha 1$ - $\alpha 2$ - $\beta 2$	10.4	4.1	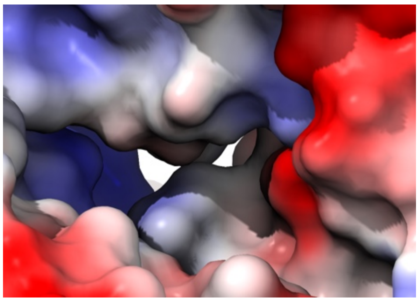

Figure S7. Sequence alignments of the spinach 26S proteasome isoforms.

The alignments show the different isoforms of the 26S proteasome subunits that were found in our preparation of *Spinacia oleracea*, including the calculated identity and similarity of their sequence.

<u>Isoforms of the CP:</u>

```
alpha3A MSRRYDSRTTIFSPEGRLYQVEYAMEAIGNAGSAIGILAKDGVVLIGEKKVTSKLLQTST 60
alpha3B MSRRYDSRTTIFSPEGRLYQVEYAMEAIGNAGSAIGILANDGVVLVGEKKVTSKLLQTST 60
        ***************************************:*****:**************

alpha3A STEKMYKIDDHVACAVAGIMSDANILINTARVQAQRYTFSYQEPMPVEQLVQSLCDTKQG 120
alpha3B SNEKMYKIDDHVACAVAGIMSDANILINTARVQAQRYTFSYQESMPVEQLVQSLCDTKQG 120
        *.***************************************** ****************

alpha3A YTQFGGLRPFGVSFLFAGWDKNYGFQLYMSDPSGNYGGWKATAIGANNQAAQSMLKQDYK 180
alpha3B YTQFGGLRPFGVSFLFAGWDKNYGFQLYMSDPSGNYGGWKAMAIGANNQAAQSMLKQDYK 180
        ***************************************** ******************

alpha3A DDVTREDAVKLALKVLSKTMDSTSLTSEKLELAEVYLLPSGKVKYQVHSPESLNRLLTES 240
alpha3B DDINREGAVQLALKVLGKTMDSTSLTSEKLELAEVYLLPSGKVKYQVHSPESLNELLAIA 240
        **:.**.**:******.*************************************.**: :

alpha3A GLTQPAAETS 250
alpha3B GLTQPAAEAS 250
        ********:*
```

Identity 94% - Similarity 95.6%

```
alpha3A MSRRYDSRTTIFSPEGRLYQVEYAMEAIGNAGSAIGILAKDGVVLIGEKKVTSKLLQTST 60
alpha3C MSRRYDSRTTIFSPEGRLYQVEYAMEAIGNAGSAIGILAKDGVVLIGEKKVTSKLLQTST 60
        ************************************************************

alpha3A STEKMYKIDDHVACAVAGIMSDANILINTARVQAQRYTFSYQEPMPVEQLVQSLCDTKQG 120
alpha3C STEKMYKIDDHVACAVAGIMSDANILINTARVQAQRYTFSYQEPMPVEQLVQSLCDTKQG 120
        ************************************************************

alpha3A YTQFGGLRPFGVSFLFAGWDKNYGFQLYMSDPSGNYGGWKATAIGANNQAAQSMLKQDYK 180
alpha3C YTQFGGLRPFGVSFLFAGWDKNYGFQLYMSDPSGNYGGWKATAIGANNQAAQSMLKQDYK 180
        ************************************************************

alpha3A DDVTREDAVKLALKVLSKTMDSTSLTSEKLELAEVYLLPSGKVKYQVHSPESLNRLLTES 240
alpha3C DDVTREDAVKLALKALSKTMDSTSLTSEKLELAEVYLLPSGKVKYQVHSPESLNRLLTES 240
        **************.*********************************************

alpha3A GLTQPAAETS 250
alpha3C GLTQPAAETS 250
        **********
```

Identity 99.6% - Similarity 99.6%

```
alpha3B MSRRYDSRTTIFSPEGRLYQVEYAMEAIGNAGSAIGILANDGVVLVGEKKVTSKLLQTST 60
alpha3C MSRRYDSRTTIFSPEGRLYQVEYAMEAIGNAGSAIGILAKDGVVLIGEKKVTSKLLQTST 60
        ***************************************:*****:**************

alpha3B SNEKMYKIDDHVACAVAGIMSDANILINTARVQAQRYTFSYQESMPVEQLVQSLCDTKQG 120
alpha3C STEKMYKIDDHVACAVAGIMSDANILINTARVQAQRYTFSYQEPMPVEQLVQSLCDTKQG 120
        *.***************************************** ****************

alpha3B YTQFGGLRPFGVSFLFAGWDKNYGFQLYMSDPSGNYGGWKAMAIGANNQAAQSMLKQDYK 180
alpha3C YTQFGGLRPFGVSFLFAGWDKNYGFQLYMSDPSGNYGGWKATAIGANNQAAQSMLKQDYK 180
        ***************************************** ******************

alpha3B DDINREGAVQLALKVLGKTMDSTSLTSEKLELAEVYLLPSGKVKYQVHSPESLNELLAIA 240
alpha3C DDVTREDAVKLALKALSKTMDSTSLTSEKLELAEVYLLPSGKVKYQVHSPESLNRLLTES 240
        **:.**.**:****.*.*************************************.**: :

alpha3B GLTQPAAEAS 250
alpha3C GLTQPAAETS 250
        ********:*
```

Identity 93.6% - Similarity 95.2%

```
alpha7A MSSIGTGYDLSVTTFSPDGRVFQIEYAAKAVDNSGTAVGIKCKDGIVLGVEKLIQSKMML 60
alpha7B MSSIGTGYDLSVTTFSPDGRVFQIEYASKAVDNSGTAVGIKCKDGIVLGVEKLIQSKMML 60
        ***************************:********************************

alpha7A PGSNRRIHSVHRHSGMAVAGLAADGRQIVARAKSEATNYESVYGEAVPVKELADRVASYV 120
alpha7B PGSNRRIHSVHRHSGMAVAGLAADGRQVVARAKSEATNYESVYGEAVPVKELADRVASYV 120
        ***************************:********************************

alpha7A HLCTLYWWLRPFGCGVILGGYDRDGPQLYMVEPSGISYRYFGAAIGKGKQAAKTEIEKLK 180
alpha7B HLCTLYWWLRPFGCGVILGGYDRDGPQLYMVEPSGISYRYFGAAIGKGKQAAKTEIEKLK 180
        ************************************************************

alpha7A LSEMTCREGIIEVAKIIYKVHDEAKDKAFELEMSWICDESKREHQKVPDNLLQEAKAAAT 240
alpha7B LSEMSCREGIIEVAKIIYKVHDEAKDKAFELEMSWICDESKREHQKVPDNLVEEAKAAAT 240
        ****:**********************************************::*******

alpha7A AALEEMDAD 249
alpha7B AALEEMDAD 249
        *********
```

Identity 98% - Similarity 100%

Isoforms of the AAA-ATPase:

```
RPT1A MAIEHEDDLKDEKNPRPLDEDDIALLKTYGLGPYSASIKKVEKEIKDMSKKVNDLIGIKE 60
RPT1B MGTEHED--DEDKKVTPLDEFDIEFLKTYNSSAYSTSIDNLEKEIREIVQRVADLRGIEE 58
      *. ****  .::*:  **** ** :****. . **:**.::****::: ::* ** **:*

RPT1A SDTGLAAPSQWDLVSDKQMMQEEQPLQVARCTKIINPNTEDAKYVINVKQIAKFVVGLGD 120
RPT1B SDTGLATPDKWDLKSDRQMWERS--------------------------LMRKMIVKLDG 92
      ******:*.:*** **:** :..                           : *::* *..

RPT1A KVSPTDIEEGMRVGVDRNKYQIQIPLPPKIDPSVTMMTVEEKPDVTYNDVGGCKEQIEKM 180
RPT1B RVSPTDIEEGMRVGVDRITWQIRMPLPPRHHRDASKSIIEEKPDITYNDIGGCKEQIQQI 152
      :**************** .:**::****: . ..:   :*****:****:*******:::

RPT1A REVVELPMLHPEKFVKLGIDPPKGVLCYGPPGTGKTLLARAVANRTDACFIRVIGSELVQ 240
RPT1B REIVELPMVAPEKFVRLGIEPPKGVLLYGPPGTGKTLVAKAVANRTDACFIRVNGTELVR 212
      **:*****: *****:***:****** **********:*:************* *:***:

RPT1A KYVGEGARMVRELFQMARSKKACIVFFDEVDAIGGARFDDGVGGDNEVQRTMLEIVNQLD 300
RPT1B KSPGQGAVMVRQLF------KGCIIFFDEVDAIGGARYFDGAGGDYEVQRTMLEIVNQLD 266
      *  *:** ***:**      *.**:************: **.*** **************

RPT1A GFDARGNIKVLMATNRPDTLDPALLRPGRLDRKVEFGLPDLEGRTQIFKIHTRTMNCERD 360
RPT1B GFDARGNVKVLMATNRPDTLDPALVRPGRMDRKVEFGLPDLEGRTQIFRIHTKNMSIKRG 326
      *******:****************:****:******************:***:.*. :*.

RPT1A IRFELLARLCPNSTGADIRSVCTEAGMYAIRARRKTVTEKDFLDAVNKVIKGYQKFSATP 420
RPT1B IRFELLARLCPNCTGADLRSVCNEAGMFAIRARRKIVTEKDFLDAVHKVISGYHKFSATP 386
      ************.****:****.****:******* **********:***.**:******

RPT1A KYMVYN 426
RPT1B EYMLYN 392
      :**:**
```

Identity 66.7% - Similarity 78.4% (RPT1B was not found in the mass spectrometry data)

```
RPT2A MGQNSSGLNRQGGPPGDRKDGEKKEKKYEPAAPPARVGRKQRKQKGSESAARIPTVTPLT 60
RPT2B MGQGPSGLNRPPNSGDKNQNSDKKDKKFEPAAPPARVGRKQRKQRGPDAAARVPVVTPHT 60
      ***. *****  .  ...::.:**:**:****************:* ::***:*.*** *

RPT2A KCKLRLLKLERIKDYLLMEEEFVANQERLKPQEEKTEEDRSKVDDIRGSPMSVGSLEELI 120
RPT2B KCKLRLLKLERVKDYLLMEEEFVANQERLKPQEEKTEEDRSKVDDIRGSPMSVGNLEELI 120
      ***********:******************************************.*****

RPT2A DENHAIVSSSVGPEYYVSIMSFVDKDQLEPGCSILMHNKVLSVVGLLQDEVDPMVSVMKV 180
RPT2B DENHAIVSSSVGPEYYVGIMSFVDKDQLEPGCSILMHNKVLSVVGILQDEVDPMVSVMKV 180
      *****************.***************************:**************

RPT2A EKAPLESYADIGGLDPQIQEIKEAVELPLTHPELYEDIGIKPPKGVILYGEPGTGKTLLA 240
RPT2B EKAPLESYADIGGLDPQIQEIKEAVELPLTHPELYEDIGIKPPKGVILYGEPGTGKTLLA 240
      ************************************************************

RPT2A KAVANSTSATFLRVVGSELIQKYLGDGPKLVRELFRVADDLSPSIVFIDEIDAVGTKRYD 300
RPT2B KAVANSTSATFLRVVGSELIQKYLGDGPKLVRELFRVADELSPSIVFIDEIDAVGTKRYD 300
      **************************************:*********************

RPT2A AHSGGEREIQRTMLELLNQLDGFDSRGDVKVILATNKIESLDPALLRPGRIDRKIEFPLP 360
RPT2B AHSGGEREIQRTMLELLNQLDGFDSRGDVKVILATNKIESLDPALLRPGRIDRKIEFPLP 360
      ************************************************************

RPT2A DIKTRRRIFTIHTSKMTLSDDVNLEEFVMTKDEFSGADIKAICTEAGLLALRERRMKVTH 420
RPT2B DIKTRRRIFTIHTSKMTLAEDVNLEEFVMTKDEFSGADIKAICTEAGLLALRERRMKVTH 420
      ******************::****************************************

RPT2A TDFKKAKEKVMFKKKEGVPEGLYM 444
RPT2B VDFKKAKEKVMYKKKEGVPEGLYM 444
      .**********:************
```

Identity 93.1% - Similarity 96.9%

```
RPT4A MATEEDVKRRAATSEYNKKLLQHRELESRSRKVKEELRSAKKDYTKTEDDLKSLQSVGQI 60
RPT4B MSTEEEVKRKAAVSEYNKKLLQHRELESRSRKVKEELRSAKKDYTKTEDDLKSLQSVGQI 60
      *:***:***:**.***********************************************

RPT4A IGEVLRPLDDERLIVKASSGPRYVVGCRSKVDKEKLTSGTRVVLDMTTLTIMRALPREVD 120
RPT4B IGEVLRPLDNERLIVKASSGPRYVVGCRSKVDKEKLTSGTRVVLDMTTLTIMRALPREVD 120
      *********:**************************************************

RPT4A PVVYNMLHEDPGNISYSAVGGLSDQIRELRESIELPLMNPELFIRVGIKPPKGVLLYGPP 180
RPT4B PVVYNMLHEDPGNISYSAVGGLSDQIRELRESIELPLMNPELFIRVGIKPPKGVLLYGPP 180
      ************************************************************

RPT4A GTGKTLLARAIASNIDANFLKVVSSAIIDKYIGESARLIREMFNYAREHQPCIIFMDEID 240
RPT4B GTGKTLLARAIASNIDANFLKVVSSAIIDKYIGESARLIREMFNYAREHQPCIIFMDEID 240
      ************************************************************

RPT4A AIGGRRFSEGTSADREIQRTLMELLNQLDGFDQLGKVKMIMATNRPDVLDPALLRPGRLD 300
RPT4B AIGGRRFSEGTSADREIQRTLMELLNQLDGFDQLGKVKMIMATNRPDVLDPALLRPGRLD 300
      ************************************************************

RPT4A RKIEIPLPNEQSRMEILKIHGAGIAKHGEIDYEAVVKLAEGFNGADLRNICTEAGMSAIR 360
RPT4B RKIEIPLPNEQSRMEILKIHAAGIAKHGEIDYEAVVKLAEGFNGADLRNICTEAGMSAIR 360
      ********************.***************************************

RPT4A AERDYVIHEDFMKAVRKLNEAKKLESSATYSADFGKD 397
RPT4B AERDYVIHEDFMKAVRKLNEAKKLESSATYSADFGKD 397
      *************************************
```

Identity 98.5% - Similarity 99.5%

```
RPT6A --------MATMEADQKTVKQGEGLRQYYLQHIHELQLRVRNKNHNLQRLEAQRNDLNSH 52
RPT6B MATALTERSSPATMGGTATTHGDGLRQYYLKRINELEHLHRQKTLDLSRLEAHRNELNSQ 60
               :    . .:..:*:*******::*:**:   *:*. :*.****:**:***:

RPT6A VRALKEELQLLQEPGSYVGEVVKVMGKSKVLVKVHPEGKYVVDIDKNIDITKLTPTTRVA 112
RPT6B VRMLKEELMLLQEPGSYVGEVVKVMGKSKVLVKVHPEGKYVVDVDKSIDITKLTPSTRVA 120
      ** ***** **********************************:**.********:****

RPT6A LRNDSYVLHLVLPSKVDPLVNLMKVEKVPDSTYDMIGGLDQQIKEIKEVIELPIKHPELF 172
RPT6B LRNDSYVLHLVLPSKVDPLVNLMKVEKVPDSTYDMIGGLDQQIKEIKEVIELPIKHPELF 180
      ************************************************************

RPT6A ESLGIAQPKGVLLYGPPGTGKTLLARAVAHHTDCTFIRVSGSELVQKYIGEGSRMVRELF 232
RPT6B ESLGIAQPKGVLLYGPPGTGKTLLARAVAHHTDCTFIRVSGSELVQKYIGEGSRMVRELF 240
      ************************************************************

RPT6A VMAREHAPSIIFMDEIDSIGSTRMESGSGNGDSEVQRTMLELLNQLDGFEASNKIKVLMA 292
RPT6B VMAREHAPSIIFMDEIDSIGSARMESGSGNGDSEVQRTMLELLNQLDGFEASKKIKVLMA 300
      *********************:******************************:*******

RPT6A TNRIDILDPALLRPGRIDRKIEFPNPTEESRFDILKIHSRRMNLMRGIDLKKIGDKMNGA 352
RPT6B TNRIDILDPALLRPGRIDRKIEFPNPNEDSRGDILKIHSRKMNLMRGIDLKKIAEKMNGA 360
      **************************.*:** ********:************.:*****

RPT6A SGAELKSVCTEAGMFALRERRIHVTQEDFEMAVAKVMKKDTDKNMSLRKLWK 404
RPT6B SGAELKAVCTEAGMFALRERRIHVTQEDFEMAVAKVMKKDNEKNMSLRKLWK 412
      ******:*********************************.:**********
```

Identity 87.7% - Similarity 91.1%

Isoforms of the RP:

```
RPN5A --------------------MDNGGNLEAQIDALLNVEKQMRLAGDVAGTRKAACDILDL 40
RPN5B FSRNPLISDDSFLSLQLAMADQKGGNLEAQIDALLNVGKQMRLFGDVDGTRKASCDILDL 60
                           ::************** ***** *** *****:******

RPN5A CFQSKAWKTLNDQIVVLSKRRGQLKQAVTAMVQQAMGYIDQTPDLDIRVELIKTLNSVSA 100
RPN5B CFQSKAWETLNDQIVALSKR---FEQAVTSMVQQAMGYIDQTPDLHTRVDLIKTLNRVSA 117
      *******:*******.****   ::****:***************. **:****** ***

RPN5A GKIYVELERARLIKILAKIKEQQGLIDEAAELMQEIAVETFGAMAKTEKIAFILEQVRLC 160
RPN5B GKIYVEVERARLIKILAKIKEQQGLIDEAAELMQEIAVETFGAMAKTEKIAFILEQVRLC 177
      ******:*****************************************************

RPN5A LDRKDYIRAQILSRKISPRVFDIDPSKEKKKPKEGDNIVEEAPADIPSLPELKRIYYELM 220
RPN5B LDRKDYIRAQILSRKISPRVFDIDPSKEKEKPKEGDNIVEEAPADIPSLTELKRIYYELM 237
      *****************************:******************* **********

RPN5A IRYYKHHNDYLEICRCYKSIYEISSVKEDPEQWTPILRKICWYLALAPHDPMQSSLLNST 280
RPN5B IRYYKHHNDYLEICRCYKAIYEISSVKEDPEQWTPILRKICWYLALAPHDPMQSSLMNST 297
      ******************:*************************************:***

RPN5A LEDKNLFEIPKFKSLLKQLVTMEVILWTVLWNEFESEFDNEKNLLGGPLGEKAGEDLKQR 340
RPN5B LEDKNLYEIPKFKSLLKQLVTIKVILWTDLWNEFEGEFDNEKNLLGGSLGEKAAEDLKQR 357
      ******:**************::***** ******.*********** *****.******

RPN5A VIEHNILVISKYYSRITLKRLSDLLCLSLQEAEKHLSDMVVSKALIAKIDRPMGIVCFQV 400
RPN5B VIEHNILVISKYNSKITLKRLSDLLCLSIQETEKHLSDMVVSKAIVAKIDRPMGIVCFQV 417
      ************ *:*************:**:************::**************

RPN5A VKDSNDILNSWSMNLEKLLDLVEKSCHQIHKETMVHKASLEV 442
RPN5B VKDSNDILNSWSINLEKLLGLVEKSCHQIHKETMIHKAALKV 459
      ************:******.**************:***:*:*
```

Identity 86.6% - Similarity 90.9%

```
RPN6A MATFLPATTDSLAQALEASSTADSILILQRLLADPSSSPDALRIKEQAITKLTDYLRQEN 60
RPN6B MATFLPATTDSLAQALEANSTADSILILQRLLADPSSSPDALRIKEQAITKLTDCLRQEN 60
      *****************.*********************************** ******

RPN6A KAEDLRILLTQLRSYFSLIPKAKTAKIVRVIIDTVAKIPNSTELQISLCKDMIQWTRDEK 120
RPN6B KAEDLRILLTQLRSYFSLIPKAKTAKIVRGIIDTVAKIPNSTELQISLCKDMVQWTRDEK 120
      ***************************** **********************:*******

RPN6A RTFLRQRVEARLAALLMETKEFPEALSLLSGLIKEVRRLDDKLLLVDIELLESKLHFSLR 180
RPN6B RTFLRQRVEARLAALLMETKEYPEALSLLSGLIKEVRRLDDKLLLVDIELLESKLHFSLR 180
      *********************:**************************************

RPN6A NLPKAKAALTAARTAANAIYVPPAQQGTIDLQSGILHAEEKDYKTGYSYFYEAFEAFNAL 240
RPN6B NLPKAKAALTAARTAANAIYVPPAQQGTIDLQSGILHAEEKDYKTGYSYFYEAFEAFNAL 240
      ************************************************************

RPN6A EDPRAVYSLKYMLLCKVMVNQADDVAGIISSKAGLQYLGPDLDAMKAIADAYSKRSLKLF 300
RPN6B DDPRAVYSLKYMLLCKVMVNQADDVAGIISSKAGLKYLGPELDAMKAIADAYSKRSLKLF 300
      :*********************************:****:*******************

RPN6A EASLVNFKAQLQEDPIIHRHLSSLYDTLLEQNLCRLIEPFSKVEISHIAELIELPVDHVE 360
RPN6B EASLVNFKAQLQEDPIIHRHLSSLYDTLLEQNLCRLIEPFSRVEISHIAELIELPVDHVE 360
      *****************************************:******************

RPN6A RKLSQMILDKKFAGTLDQGAGCLIIFDDHKTEDIYEATLDTISNVAKVVDSLFVRSAKIM 420
RPN6B RKLSQMILDKKFAGTLDQGAGCLIIFDDPKTEAIYEATLDTISNVAKVVDSLFVRSAKIM 420
      **************************** *** ***************************

RPN6A A 421
RPN6B A 421
      *
```

Identity 97.4% - Similarity 99%

```
RPN8A MDVIKTQQIFSSKSIEKVVVHPLVLLSIVDHYNRVARDTKKRVIGVLLGSTFKGTVDVTN 60
RPN8B MDVVKIQQISSSKAIEKVVVHPLVLLSIVDHYNRVARDTKKRVIGVLLGSTFKGTVDVTN 60
      ***:* *** ***:**********************************************

RPN8A SYAVPFEEDDKDSSIWFLDHNYHESMFSMFRRINAKEHVVGWYSTGPKLRENDLDVHRLF 120
RPN8B SYAVPFEEDDKDPSIWFLDHNYHESMFSMFRRINAKEHVVGWYSTGPKLRENDLDVHRLF 120
      ************ ***********************************************

RPN8A SDYVPNPVLVIIDVQPEELGIPTKAYYAVEEVKENATQKSQKVFVHVPSEIAAHEVEEIG 180
RPN8B SDYVPNPVMVIIDVQPEELGIPTKAYYAVEEVKENATQKSQKVFAHVPSEIAAHEVEEIG 180
      ********:***********************************.***************

RPN8A VEHLLRDVKDTTISTLATEVTGKLGALKGLDARLREIRSYLELVIQEKLPLNHEILYHLQ 240
RPN8B VEHLLRDVKDTTISTLATEVTGKLAALKGLDARLREIRGYLELVIEGKLPLNHEILYHLQ 240
      ************************.*************.******: *************

RPN8A DVFNLLPNLSVLELVKAFAVKTNDMMLVIYLSSLIRSVIALHNLINNKMLNKEHEKAEDS 300
RPN8B DVFNLLPNLSVLELVKAFAVKTNDMMLVIYLSSLIRSVIALHNLINNKMLNKEHEKSEDS 300
      ********************************************************:***

RPN8A KSLAITSVAGS 311
RPN8B KSIPVVAAAGG 311
      **: :.:.**.
```

Identity 93.9% - Similarity 96.5% (RPN8B was not found in the mass spectrometry data)

```
RPN9A MSALQYLDTLRSAHPELGEWYNTLADLYQKKLWHQLTLELEKFVALAVFQAGDALIQLYH 60
RPN9B MSAIEYLEAVNSSNPELGDWYSSFAHLYTKELWHQLTVELEKFIALPVFQTGDALIPFYH 60
      ***::**:::.*::****:**.::*.** *:******:*****:** ***:***** :**

RPN9A NFITDFETKINLLKLAHFAVIVSRQYAEKEAAIGYLEGVTEKLHATKENRIEEPVLYIKM 120
RPN9B NFITDFETKINLLKLAHFTVIVSRQYAEKEAAIGYLEGVIEKLHATKENRIEEPVLYVKM 120
      *****************:******************** *****************:**

RPN9A QIALFKLEQGEQKECKKLLDNGKTTLDSMTDIDPSVYATFYWVSSQYHKARQEFAEFYKN 180
RPN9B QIALLKLEQGEHKACKKLLEDGKTTLDSMTDIDPSVYASFYWVSSQYHKARQEFAEFYKS 180
      ****:******:* *****::*****************:********************.

RPN9A ALLYLAYTSVESLSESFKLDLAFDLSLSALLGENIYNFGELLAHPILKSLLGTKVEWLYY 240
RPN9B ALLYLAYTSVESLSESFKLDLAFDLSLSALLGENIYNFGELLAHPILKSLLGTKAEWLYY 240
      ******************************************************.*****

RPN9A ILQAFNTGDLIRYQELCNVHKDALNAQPALVANERKLLEKINILCLMEIIFNRPAEDRTI 300
RPN9B ILQAFNSGNLIRYQELCNVHKDALNAQPALVANERKLLEKINILCLMEIIFNRPAEDRTI 300
      ******:*:***************************************************

RPN9A PLKVIAERTRLSIEDVEYLLMKSLSVHLIEGIIDQMEGTVHVSWVQPRVLGIPQITSLRD 360
RPN9B PLKVIADRTRLSIEDVEYLLMKSLSVHLIEGIIDQVEGTVHVSWVQPRVLGIPQITALRA 360
      ******:****************************:********************:**

RPN9A RLDSWLGKVNTALLSVEAETPDLVAS 386
RPN9B RLDNWLGKVHTTLLSVEAETPDLIAS 386
      ***.*****:*:***********:**
```

Identity 89.1% - Similarity 95.6%

```
RPN11A MDRLTRMMAGAGGALGHPPPDSPTLDTSEQVYISSLALLKMLKHGRAGVPMEVMGLMLGE 60
RPN11B MDRLTRMFAGAGGALGHPPPDSPTLDTSEQVYISSLALLKMLKHGRAGVPMEVMGLMLGE 60
       *******:****************************************************

RPN11A FVDEYTVKVVDVFAMPQSGTGVSVEAVDPVFQTNMLDMLKQIGRPEMVVGWYHSHPGFGC 120
RPN11B FVDEYTVRVVDVFAMPQSGTGVSVEAVDHVFQTNMLDMLKQTGRPEMVVGWYHSHPGFGC 120
       *******:******************** ************ ******************

RPN11A WLSGVDINTQQSFEALNQRAVAVVVDPIQSVKGKVVMDAFRLINPQTMMLGQEPRQTTSN 180
RPN11B WLSGVDINTQQSFEALNQRAVAVVVDPIQSVKGKVVIDAFRLINPQTMMLGQEPRQTTSN 180
       ************************************:***********************

RPN11A LGHLNKPSIQALIHGLNRHYYSIAINYRKNELEEKMLLNLHKKKWNDGLTLKKFDVHSKT 240
RPN11B LGHLNKPSIQALIHGLNRHYYSIAINYRKNELEEKMLLNLHKKKWTNGLTLKRFDDHSKT 240
       *********************************************.:*****:** ****

RPN11A NEQTVQEMLGLAIKYNKAVQEEDELTPEKLVIAKVGRQDAKKHLEEHVSNLMSSNIIQTL 300
RPN11B NEQTVQEMLNLAIKYNKAVQEEDELTPEKLAIANVGRQDAKKHLEEHVSNLMSSNIVQTL 300
       *********.********************.**:**********************:***

RPN11A GTMLDTVIF 309
RPN11B GTMLDTVVF 309
       *******:*
```

Identity 95.5% - Similarity 97.4%

Table S1. 26S proteasome subunits nomenclature.

List of all subunits of the spinach 26S proteasome identified by mass spectrometry. The different isoforms are marked with (A) – representing the most abundant isoform – (B) or (C). The isoforms with an asterisk (*) couldn't be found in the mass spectrometry data.

Unified Nomenclature	Plant Gene Name	Spinach Gene Name (Uniprot)	Known Human Gene Name (Uniprot)	Known Yeast Gene Name (Uniprot)
**20S proteasome**				
α1	PAA	SOVF_093600	PSMA6 / PROS27	SCL1 / PRC2 / PRS2 / YC7
α2	PAB	SOVF_201960	PSMA2 / HC3 / PSC3	PRE8 / PRS4 / YC7
α3	PAC (A)<br>PAC (B)<br>PAC (C)	SOVF_067770<br>SOVF_188980<br>PAC1	PSMA4 / HC9 / PSC9	PRE9 / PRS5 / Y13
α4	PAD	SOVF_013850	PSMA7 / HSPC	PRE6
α5	PAE	SOVF_076310	PSMA5	PUP2 / DOA5
α6	PAF	SOVF_075310	PSMA1 / HC2 / NU / PROS30 / PSC2	PRE5
α7	PAG (A)<br>PAG (B)	PAG1<br>SOVF_007100	PSMA3 / HC8 / PSC8	PRE10 / PRC1 / PRS1 / YC1
β1	PBA	SOVF_007620	PSMB6 / LYMPY / Y	PRE3
β2	PBB	SOVF_003170	PSMB7 / Z	PUP1
β3	PBC	SOVF_015190	PSMB3	PUP3
β4	PBD	SOVF_145550	PSMB2	PRE1
β5	PBE	SOVF_078180	PSMB5 / LMPX / MB1 / X	PRE2 / DOA3 / PRG1
β6	PBF	SOVF_035230	PSMB1 / PSC5	PRE7 / PRS3 / PTS1
β7	PBG	SOVF_076470	PSMB4 / PROS26	PRE4
**19S regulator ATPase subunits**				
RPT1	RPT1 (A)<br>RPT1 (B)*	RPT1<br>SOVF_004100	PSMC2 / MSS1	YTA3 / CIM5
RPT2	RPT2 (A)<br>RPT2 (B)	SOVF_167680<br>SOVF_147780 / _210740	PSMC1	YTA5/mts2 / YHS4
RPT3	RPT3	SOVF_151020	PSMC4 / MIP224 / TBP7	YNT1 / YTA2
RPT4	RPT4 (A)<br>RPT4 (B)	SOVF_027030<br>SOVF_027040	PSMC6 / SUG2	SUG2 / PCS1 / CRL13
RPT5	RPT5	SOVF_156090	PSMC3 / TBP1	YTA1
RPT6	RPT6 (A)<br>RPT6 (B)	SOVF_183190<br>SOVF_175230	PSMC5 / SUG1	SUG1
**19S regulator non-ATPase subunits**				
RPN1	RPN1	SOVF_094190	PSMD2	HRD2 / NAS1/mts4
RPN2	RPN2	SOVF_045290	PSMD1	SEN3
RPN3	RPN3	SOVF_099120	PSMD3	SUN2
RPN5	RPN5 (A)<br>RPN5 (B)	SOVF_139310<br>SOVF_160100	PSMD12	NAS5
RPN6	RPN6 (A)<br>RPN6 (B)	SOVF_197890<br>SOVF_054100	PSMD11	NAS4
RPN7	RPN7	SOVF_135550	PSMD6	RPN7

RPN8	RPN8 (A)<br>RPN8 (B)*	SOVF_020490	PSMD7	NAS3
RPN9	RPN9 (A)<br>RPN9 (B)	SOVF_035360<br>SOVF_035380	PSMD13	NAS7/mts1
RPN10	RPN10	SOVF_154600	PSMD4	SUN1 / MCB1/pus1
RPN11	RPN11 (A)<br>RPN11 (B)	SOVF_184120<br>SOVF_184110	PSMD14	MPR1/pad1 / mts5
RPN12	RPN12 (A)	SOVF_037910	PSMD8	NON1/mts3
RPN13	RPN13	SOVF_136340	PSMD16 / ADRM1	DAQ1
RPN15	DSS1	SOVF_131370	PSMD15 / SEM1	SEM1

Table S2. Software and Algorithms.

	Source	
CHAINSAW	Stein, 2008	
ChimeraX	Goddard et al., 2018	https://www.cgl.ucsf.edu/chimerax/
Coot	Emsley and Cowtan, 2004	https://www2.mrc-lmb.cam.ac.uk/personal/pemsley/coot
Cow		http://www.cow-em.de
Gautomatch		https://www.mrc-lmb.cam.ac.uk/kzhang
MolProbity	Chen et al., 2010	http:/molprobity.biochem.duke.edu
MotionCor2		https://emcore.ucsf.edu/ucsf-software
Phenix	Adams et al., 2010	https://www.phenix-online.org
Relion	Zivanov et al., 2019	http://www2.mrc-lmb.cam.ac.uk/relion
ResMap	Kucukelbir et al., 2014	http://resmap.sourceforge.net
SerialEM	Mastronarde, 2005	https://bio3d.colorado.edu/SerialEM/
UCSF-Chimera	Pettersen et al., 2004	https://www.cgl.ucsf.edu/chimera

Table S3. Electron cryo microscopy data collection, refinement, and validation statistics.

	Data 1 / 2 / 3	
Data collection		
EM equipment	FEI Titan Krios	
Magnification	81,000	
Voltage (kV)	300	
Detector	Falcon 3 EC	
Pixel size (Å)	1.058	
Electron dose / Electron exposure ($e^-/Å^2$)	80 / 50 / 80	
Defocus range (μm)	2 - 4.5	
Symmetry imposed	C1	
Initial particle images (no.)	7996 / 8089 / 6773	
Final particle images (no.)	4718 / 4602 / 3987	
Map resolution (Å)	3.3	
FSC threshold	0.143	
Map resolution range (Å)	2.8 - 7	
Reconstruction		
Software	Relion, cryoSPARC	
Number of used particles	951,422	
Symmetry	C1	
Final resolution (Å)	3.3	
Map-sharpening *B* factor ($Å^2$)	109.9	
Model building		
Software	Chainsaw, Coot, Phenix	
Refinement		
Software	Phenix	
Average FSC	1.9	
	20S	19S
Model composition		
Protein residues	6256	6993
R.m.s. deviations		
Bond lengths (Å)	0.009	0.006
Bond angles (°)	1.190	1.188
Validation		
MolProbity score	1.95	1.77

Clash score	10.31	7.64
Poor rotamers (%)	0.70	0.00
Ramachandran plot		
Favored (%)	93.81	94.94
Allowed (%)	6.06	4.83
Outliers (%)	0.13	0.23

Movie S1. Movies of all mentioned states.

(A) Different modes from all used tools (Relion, CowSuite, cryoSPARC, PCA, cryoDRGN). **(B)** 2D classes of the bovine 26S proteasome combined in a video to show the compression movement.
